# Cyclohexanediamine Triazole (CHDT) Functionalization Enables Labeling of Target Molecules with Al^18^F/^68^Ga/^111^In

**DOI:** 10.1021/acs.bioconjchem.4c00313

**Published:** 2024-08-26

**Authors:** Wiebke Sihver, Martin Walther, Martin Ullrich, Anne-Kathrin Nitt-Weber, Jenny Böhme, Falco Reissig, Magdalena Saager, Kristof Zarschler, Christin Neuber, Jörg Steinbach, Klaus Kopka, Hans-Jürgen Pietzsch, Robert Wodtke, Jens Pietzsch

**Affiliations:** † Helmholtz-Zentrum Dresden-Rossendorf, Institute of Radiopharmaceutical Cancer Research, Bautzner Landstraße 400, 01328 Dresden, Germany; ‡ Technische Universität Dresden, School of Science, Faculty of Chemistry and Food Chemistry, Mommsenstraße 4, 01069 Dresden, Germany

## Abstract

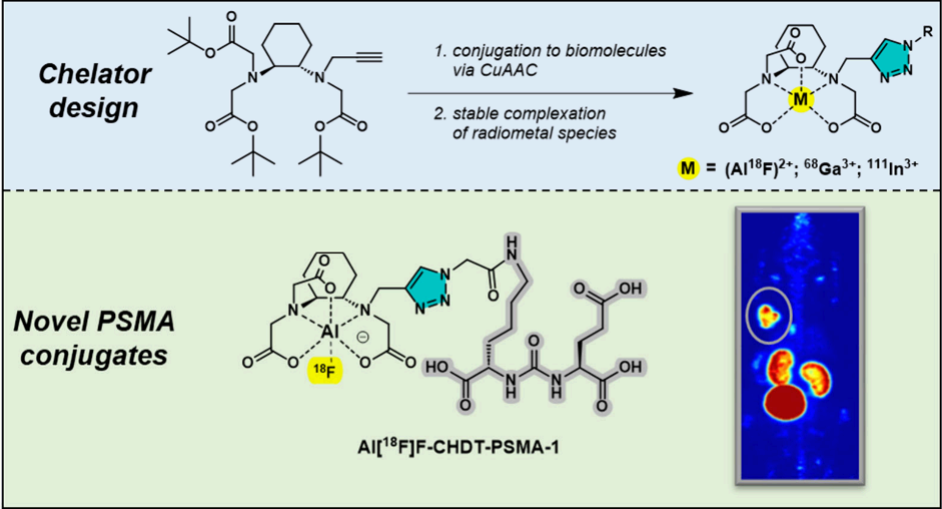

The Al^18^F-labeling approach offers a one-step access to radiofluorinated biomolecules by mimicking the labeling process for radiometals. Although these labeling conditions are considered to be mild compared to classic radiofluorinations, improvements of the chelating units have led to the discovery of **(±)-H**_**3**_**RESCA**, which allows Al^18^F-labeling already at ambient temperature. While the suitability of **(±)-H**_**3**_**RESCA** for functionalization and radiofluorination of proteins is well established, its use for small molecules or peptides is less explored. Herein, we advanced this acyclic pentadentate ligand by introducing an alkyne moiety for the late-stage functionalization of biomolecules via click chemistry. We show that in addition to Al^18^F-labeling, the cyclohexanediamine triazole (CHDT) moiety allows stable complexation of ^68^Ga and ^111^In. Three novel CHDT-functionalized PSMA inhibitors were synthesized and their Al^18^F-, ^68^Ga-, and ^111^In-labeled analogs were subjected to a detailed *in vitro* radiopharmacological characterization. Stability studies *in vitro* in human serum revealed among others a high kinetic inertness of all radiometal complexes. Furthermore, the Al^18^F-labeled PSMA ligands were characterized for their biodistribution in a LNCaP derived tumor xenograft mouse model by PET imaging. One radioligand, **Al[**^**18**^**F]F-CHDT-PSMA-1**, bearing a small azidoacetyl linker at the glutamate-urea-lysine motif, provided an *in vivo* performance comparable to that of **[**^**18**^**F]PSMA-1007** but with even higher tumor-to-blood and tumor-to-muscle ratios at 120 min *p.i.* Overall, our results highlight the suitability of the novel CHDT moiety for functionalization and radiolabeling of small molecules or peptides with Al^18^F, ^68^Ga, and ^111^In and the triazole ring seems to entail favorable pharmacokinetic properties for molecular imaging purposes.

## Introduction

Noninvasive imaging by positron emission tomography (PET) and single photon emission computed tomography (SPECT) has become an essential technique in nuclear medicine for the diagnosis of pathological conditions such as neurodegenerative diseases,^1,2^ cardiac diseases,^3^ inflammatory or infectious diseases,^4^ and cancer.^5^ For PET applications, a suitable vector molecule (small molecule, peptidomimetic, peptide, or protein) that targets the respective process of interest, e.g., a ligand that binds to a cell surface receptor, is equipped with a positron (β^+^) emitting radionuclide. Although a broad range of such radionuclides with suitable nuclear properties for PET imaging exists and their production is established,^6^ fluorine-18 is still one of the most frequently applied radionuclides. This originates from its sufficiently long half-life (109.8 min) that enables even multistep radiosyntheses and facilitates the transport of the final radiopharmaceutical to distant application sites within a satellite concept. Furthermore, the high percentage of β^+^ emission (97%) and the low positron energy (*E*_max_ = 0.635 MeV) favors the quality of the PET images.^7,8^

Driven by its almost ideal nuclear properties for PET imaging, the (radio)chemical toolbox for the introduction of fluorine-18 has expanded tremendously allowing the radiofluorination of basically all kinds of target molecules from small molecules to proteins.^9^ Generally, ^18^F-labeled radiotracers are accessed via direct labeling of their respective precursors^10^ or via indirect labeling using initially prepared ^18^F-labeled building blocks (prosthetic groups).^8^ In case of [^18^F]fluoride as ^18^F-species, direct labeling usually requires the workup of the crude aqueous solution via anion exchange and subsequent drying to provide reactive [^18^F]fluoride for efficient labeling reactions.^11^ Subsequent ^18^F-labeling is often performed in water-free organic solvents and at high temperatures. However, such conditions would usually not maintain the structural integrity of peptides and proteins. Furthermore, the structural complexity of these molecules bearing several functional groups lowers the labeling efficiency.^12^ Therefore, to enable the efficient direct labeling of such complex biomolecules, labeling strategies that rely on the formation of heteroatom- and metal–[^18^F]fluoride bond formation have been developed. In contrast to C–F bond formation, the introduction of [^18^F]fluoride is possible even under mild conditions and in the presence of water.^12,13^

Of these alternative ^18^F-labeling strategies, the Al^18^F-labeling approach appears particularly appealing.^14,15^ Based on the strong binding of fluoride to several metals, McBride *et al*.^16^ discovered the successful complexation of (Al[^18^F]F)^2+^ by common chelators including DTPA, NOTA, and NODA. In terms of complexation efficiency and complex stability, the cyclic pentadentate ligand NODA provided the best results due to the favorable N_3_O_2_ donor set, which leaves a sixth coordination site at the aluminum open for fluoride.^16−19^ Similar to labeling reactions with radiometals, the Al^18^F-labeling approach offers the opportunity for radiofluorination of target molecules via lyophilized kits.^20^ However, efficient Al^18^F-labeling usually entails temperatures of 100 °C, which is comparable to the conditions often applied for ^68^Ga- or ^64^Cu-labeling. Such high temperatures are tolerated by small peptides but certainly not by proteins.^12^

**Figure 1:**
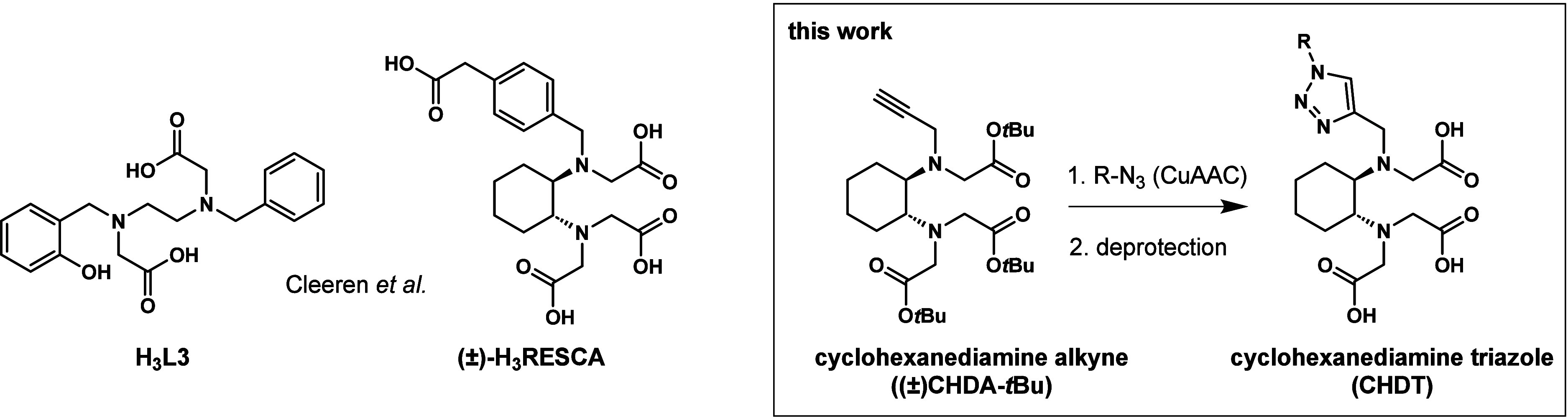
Structures of previously reported H_3_L3 and (±)-H_3_RESCA and the novel (±)-CHDA-*t*Bu for generating CHDT-functionalized molecules.

Aiming at Al^18^F-labeling at lower temperatures, Cleeren *et al*.^21^ developed acyclic pentadentate ligands with a N_2_O_3_ donor set. Of these new ligands, **H**_**3**_**L3** (Figure 1) provided an acceptable stability of the Al^18^F-complex *in vitro* and bone uptake of **Al[**^**18**^**F]F-L3** and a PSMA ligand conjugate was low at 60 min *p.i.* Later, the same group introduced a cyclohexane moiety in their ligand scaffold, resulting in the ligand called **(±)-H**_**3**_**RESCA** or *rac***-H**_**3**_**RESCA** (REStrained Complexing Agent, Figure 1) which enabled Al^18^F-labeling at very mild conditions (pH 4.6, ambient temperature) in a short period of time (12 min).^22^ Moreover, the stability of the Al[^18^F]F-RESCA-complex, in particular with regards to defluorination, is sufficient for *in vivo* applications. The introduction into biomolecules is realized by an TFP or maleimide functionalized **(±)-H**_**3**_**RESCA**,^23^ which are commercially available. However, so far only few examples of biomolecules that were labeled by the Al^18^F-RESCA-method are reported, including human serum albumin, a nanobody against the Kupffer cell marker CIRg, affibodies against HER2, and a peptidic uPAR ligand.^22,24−26^

Inspired by the general suitability of the Al^18^F-RESCA-method, we envisaged a complementary approach for the introduction of RESCA-like complexing agents into biomolecules. For this purpose, we sought to structurally modify **(±)-H**_**3**_**RESCA** by replacing the 2-(*p*-tolyl))acetic acid moiety by a propargyl moiety, which would transform into a 1,4-disubstituted-1,2,3-triazole ring upon coupling to azide-functionalized biomolecules via CuAAC (copper(I)-catalyzed azide–alkyne cycloaddition, Figure 1). Apart from this alternative functionalization strategy, we hypothesized that the triazole ring could participiate in coordination to metal ions (resulting in a N_3_O_3_ donor set), which could support thus the efficient and stable complexation of other metal ions in addition to Al^3+^. 1,2,3-Triazoles are known to participate as ligands in metal complexes including complexes of rhenium, technetium, copper, zinc, and platinum,^27−30^ but there are also occasional reports on coordination to aluminum^31^ and indium.^32^ To the best of our knowledge, studies on complexation of other metal ions, in particular radiometals for PET and SPECT imaging, by **(±)-H**_**3**_**RESCA** have not been reported so far. Furthermore, the 1,2,3-triazole ring entails an increased hydrophilicity compared to the benzyl ring in **(±)-H**_**3**_**RESCA** which might positively affect the pharmacokinetics of respective conjugates.^33^

**Scheme 1:**
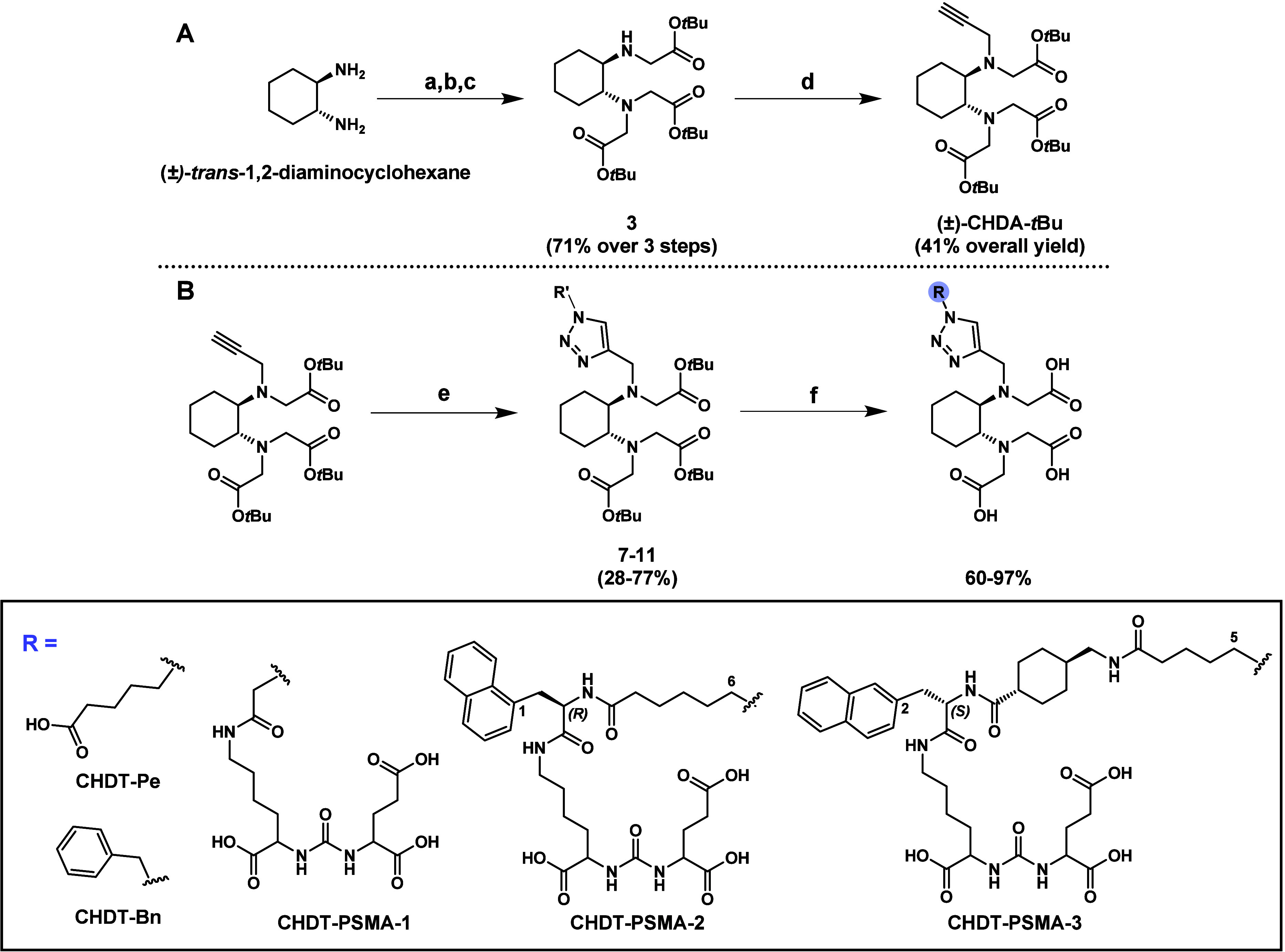
Synthesis of **(±)-CHDA-*t*Bu** (A) and of the CHDT-Functionalized Compounds (B) Reagents and conditions: (a) benzaldehyde, NaBH_4_, CH_3_OH, 12 h, Ar; (b) *tert*-butyl bromoacetate, DIPEA, CH_2_Cl_2_, 12 h; (c) Pd/C, ammonium formate, 2.5 h, 65 °C; (d) propargyl bromide, triethyl amine, CH_3_CN, 12 h; (e) R′-N_3_ (benzyl azide/5-azidopentanoic acid/**4**–**6**), CuSO_4_, sodium ascorbate, *tert*-butanol/water, 24 h; (f) TFA/CH_2_Cl_2_ (9:1, v/v), 12 h.

Herein, we present the synthesis of the novel cyclohexanediamine alkyne **(±)**-**CHDA-***t***Bu** (CycloHexaneDiamine Alkyne) in which the three acetic acid moieties are *t*Bu-protected to allow an efficient CuAAC with azide-functionalized biomolecules. **(±)**-**CHDA-***t***Bu** was then coupled to two simple model azides (5-azido pentanoic acid and benzyl azide) to generate the CHDT (CycloHexaneDiamine Triazole)-functionalized molecules **CHDT-Pe** and **CHDT-Bn**. Al^18^F-labeling but also labeling with radiometals such as ^68^Ga^3+^ and ^64^Cu^2+^ was tested and the Al^18^F-labeled compounds were subjected to *ex vivo* biodistribution studies after injection into healthy mice. Subsequently, **(±)**-**CHDA-***t***Bu** was used to prepare three novel CHDT-functionalized PSMA ligands (**CHDT-PSMA-1/2/3**, Scheme 1). PSMA (prostate specific membrane antigen) is a type II transmembrane glycoprotein with cocatalytic metallopeptidase activity^34^ and emerged as attractive target for the radionuclide diagnosis and treatment of prostate cancer,^35,36^ owing to its high abundance in this type of cancer.^37,38^ Although numerous radiolabeled PSMA ligands were developed in the past and some compounds are already in clinical use, there is still an interest in novel radioligands, in particular ^18^F-labeled ligands.^39^**CHDT-PSMA-1/2/3** were labeled with (Al^18^F)^2+^ but also ^68^Ga^3+^ and ^111^In^3+^ to demonstrate the potential of the CHDT moiety for complexing other metal ions in addition to Al^3+^. The respective nine radioligands were radiopharmacologically characterized *in vitro* with a focus on serum stability, saturation binding analyses and internalization behavior using monolayers and spheroids of PSMA-positive LNCaP cells. Moreover, the *in vivo* radiopharmacological characterization of Al^18^F-labeled **CHDT-PSMA-1/2/3** was performed in LNCaP-tumor bearing mice in comparison to the well-known PSMA ligand **[**^**18**^**F]PSMA-1007**.

## Results and Discussion

### Synthesis of (±)-CHDA-*t*Bu and the CHDT-Functionalized Compounds

The synthesis of **(±)-CHDA-***t***Bu** was accomplished in four steps starting with racemic *trans*-1,2-diaminocyclohexane in orientation to the procedure for the preparation of **(±)-H**_**3**_**RESCA-TFP** or **(±)-H**_**3**_**RESCA-Mal**.^40,41^ The first step comprised the monobenzylation by the use of benzaldehyde and sodium borohydride. This was followed by alkylation with *tert*-butyl bromoacetate under basic conditions (DIPEA). Subsequently, the benzyl group was removed by hydrogenolysis using Pd/C and ammonium formate. For the last step to **(±)-CHDA-***t***Bu**, the propargyl group was introduced by reaction with propargyl bromide and triethyl amine. All steps proceeded in good to excellent yields and **(±)-CHDA-***t***Bu** was obtained in an overall yield of 41%. The *tert*-butyl groups had to be kept for the introduction into biomolecules to enable an efficient coupling via CuAAC as the unprotected CHDT moiety is also able to complex Cu^2+^ (see below).

For model compounds **CHDT-Pe** and **CHDT-Bn**, 5-azidopentaoic acid and benzyl azide, respectively, were coupled via CuAAC. Subsequent removal of the *tert*-butyl groups by TFA treatment afforded both compounds in good yields of 46% and 81%, respectively, over these two steps. A similar procedure was followed for the synthesis of the PSMA ligands **CHDT-PSMA-1/2/3**, for which the respective *tert*-butyl protected and azide-functionalized precursor compounds (**4**–**6**) were synthesized (see Chemistry section in Supporting Information). **CHDT-PSMA-1/2/3** were obtained in overall yields of 22–65% for the coupling and deprotection steps. Regarding the linker entities between the glutamyl-urea-lysine (**KuE**) PSMA binding motif and the CHDT moiety, **CHDT-PSMA-1** was designed to bear the smallest possible azide linker (apart from the use of ε-azido-norleucine). In contrast, **CHDT-PSMA-3** was designed in orientation to **PSMA-617** (l-2NaI and *trans*-4-(aminomethyl)cyclohexanecarboxylic acid (AMCH)) by substituting the DOTA chelator with the CHDT moiety and a hexanoyl linker. For **CHDT-PSMA-2**, d-1NaI was chosen instead of l-2NaI and AMCH was omitted. Benesova *et al*.^42^ previously demonstrated that l-2NaI is better tolerated than l-1NaI due to a more favorable orientation within the binding pocket of PSMA. Moreover, l-2NaI is superior to d-2NaI. However, d-1NaI was not tested, which prompted us to test this particular amino acid in the context of the present study.

### Complexation of (Al[^18^F]F)^2+^, [^68^Ga]Ga^3+^, [^64^Cu]Cu^2+^, and [^111^In]In^3+^

Initially, **CHDT-Pe** and **CHDT-Bn** were used to test the complexation of (Al[^18^F]F)^2+^. The complexation under acidic conditions (pH 5.0) at precursor amounts of 50 μg (72 nmol) and a temperature between 25 and 40 °C proceeded with high radiochemical conversions (RCC) of >80% (Figure S1 in Supporting Information). Subsequently, we also envisaged complexation of [^68^Ga]Ga^3+^ and [^64^Cu]Cu^2+^ by the CHDT moiety. To our delight, complexation of these two radiometals was indeed possible (shown for **CHDT-Pe** in Figure S1) under similar conditions as applied for Al^18^F-labeling. This could indicate that the triazole ring participates in binding of these radiometal species as hypothesized by us. However, it is not excluded that **(±)-H**_**3**_**RESCA** is also able to complex other radiometal species but such data are not reported so far. Motivated by the initial radiolabeling experiments with **CHDT-Pe** and **CHDT-Bn**, radiolabeling of the novel CHDT-functionalized PSMA ligands, **CHDT-PSMA-1/2/3**, was performed. In addition to (Al[^18^F]F)^2+^, [^68^Ga]Ga^3+^, and [^64^Cu]Cu^2+^, we also tried the complexation of [^111^In]In^3+^. Complexation at 40 °C and a precursor amount of 10 μg (7.5, 8.3, and 10.5 nmol for **CHDT-PSMA-1/2/3**, respectively) resulted in high radiochemical conversions (>95%) for all radiometal species (Figure S2 in Supporting Information). Overall, we demonstrated that the transformation of the original **(±)-H**_**3**_**RESCA** ligand to the novel CHDT moiety still allows Al^18^F-labeling, but also labeling with [^68^Ga]Ga^3+^, [^64^Cu]Cu^2+^, and [^111^In]In^3+^.

While the ^68^Ga/^64^Cu/^111^In-labeled compounds were not further processed for their radiopharmacological characterization, residual unbound ^18^F-species were removed from the reaction mixture by the addition of hydroxyapatite. It is worth noting, that the treatment with hydroxyapatite should be conducted rather short (30 s) as it not only binds unbound ^18^F-species but leads also to decomplexation of the Al^18^F-complex, which ultimately lowers the radiochemical yield and apparent molar activity. Alternatively, unbound ^18^F-species could also be removed by solid-phase extraction using suitable cartridges. This processing would also improve radiochemical yield and molar activity values. However, herein we decided to follow the hydroxyapatite treatment to ensure [^18^F]fluoride (and Al^18^F species)-free radioligand formulations. For the radiolabeled **CHDT-PSMA-1/2/3**, radiochemical purities of >95% (Figure S3 in Supporting Information) and apparent molar activities of 10 (±1, Al^18^F), 24 (±3, ^68^Ga), and 24 (±5, ^111^In) MBq/nmol, respectively (mean ± SD values over all compounds), were achieved.

For comparative *in vitro* and *in vivo* studies, **[**^**18**^**F]PSMA-1007** (in-house prepared according to GMP and AMG) was used (RCP > 99%, Figure S3). Furthermore, as radioligand for the subsequent competition assay, **[**^**177**^**Lu]Lu-PSMA-617** was prepared under standard conditions (pH 5.0, 90 °C, 20 min, RCY > 99%, Figure S3).

### Distribution Coefficients (logD_7.4_) and Stability Studies

The distribution coefficients logD_7.4_ of the Al^18^F-, ^68^Ga-, and ^111^In-labeled PSMA conjugates were determined and are summarized in Table 1. Among the three PSMA conjugates, radiolabeled **CHDT-PSMA-1** is the most hydrophilic compound, irrespective of the radionuclide, which appears reasonable due to the short azido alkyl linker and the absence of a naphthylalanine residue compared to the other two compounds.

**Table 1:** Summary of logD_7.4_ Values and Serum Stability of the Radiolabeled PSMA Ligands^a^

		**Conjugates**		
	Radiolabel	**CHDT-PSMA-1**	**CHDT-PSMA-2**	**CHDT-PSMA-3**
logD_7.4_	Al^18^F	–3.60 ± 0.14	–3.34 ± 0.09	–3.43 ± 0.13
	^68^Ga	–3.36 ± 0.04	–3.11 ± 0.06	–3.09 ± 0.10
	^111^In	–4.23 ± 0.08	–3.44 ± 0.07	–3.87 ± 0.07
Percentage of intact radioligand after 3 h in human serum	Al^18^F	98.9 ± 0.1	99.1 ± 0.2	99.3 ± 0.1
	^68^Ga	96.6 ± 2.2^#^	97.6 ± 0.1	99.1 ± 0.4
	^111^In	99.5 ± 0.1	98.8 ± 0.4	99.2 ± 0.2
	*	99.6 ± 0.1	98.4 ± 0.4	99.1 ± 0.2
	**	99.3 ± 0.2	98.7 ± 0.7	99.4 ± 0.4

a Data shown are mean values (±SEM) of three experiments (^#^two experiments). Each experiment was performed in triplicate. Percentage of intact ^111^In-labeled conjugates after 24 h (*) and after 48 h (**). Percentage of intact radioligand was assessed by radio-TLC using iTLC-SG strips as stationary phase and 2 M ammonium acetate/methanol (1:1, v/v) as eluent. The data of the individual experiments are listed in Table S1 in Supporting Information.

Stability studies of the Al^18^F-, ^68^Ga-, and ^111^In-labeled PSMA conjugates were conducted in human serum and the percentages of intact radioligand are summarized in Table 1. Analysis of serum samples was performed by radio-TLC and radio-HPLC to correctly assess disintegration of the radiolabeled molecules caused by released radiometal species and metabolization. All compounds showed an excellent stability over 3 h with values for residual intact radioligand >96%. Moreover, the ^111^In-labeled compounds appeared also to be stable even after a prolonged incubation period of 24 and 48 h (Table 1). These results illustrate that the novel CHDT moiety provides a high kinetic inertness of the respective Al^18^F-, ^68^Ga-, and ^111^In-complexes under physiological conditions *in vitro*.

Although [^64^Cu]Cu-complexation by the CHDT moiety is also possible, the kinetic inertness of the resulting complex appears rather low. This was exemplarily demonstrated for **[**^**64**^**Cu]Cu-CHDT-PSMA-1** in a protein challenge experiments using human serum and analysis by radio-SDS-PAGE (Figure S4 in Supporting Information). There was a significant transchelation of [^64^Cu]Cu^2+^ to albumin (20%), while other copper chelators such as TETA, DOTA, NOTA, cyclam, or diamSar show only minimal ^64^Cu-transchelation under the same conditions.^43^ In contrast, for **[**^**68**^**Ga]Ga-CHDT-PSMA-1** under similar conditions no transchelation of [^68^Ga]Ga^3+^ in human serum to transferrin, which has a high affinity for Ga^3+^,^44^ was observed (Figure S5 in Supporting Information), which highlights the higher kinetic inertness of the ^68^Ga-complex compared to the ^64^Cu-complex. In this context, Ga^3+^ and In^3+^ can be classified as hard acidic cations according to the Pearson^’^s hard–soft acid–base theory and favor hard donor atoms (e.g., anionic oxygen), while Cu^2+^ is rather a borderline acid and favors soft donor atoms (e.g., nitrogen).^45^ Consequently, it appears comprehensible that the acyclic CHDT moiety with N_2_O_3_ donor set (without triazole ring) is sufficient for stable complexation of Ga^3+^ and In^3+^. In contrast, although the 1,2,3-triazole was intended to provide an additional nitrogen donor for metal complexation, this coordination (if coordination occurs at all) is not sufficient for stably complexing Cu^2+^. It is worth noting that previously a DTPA analog bearing a *trans*-1,2-diaminocyclohexane backbone (CHX-A″) has been developed for complexation of ^212^Bi,^46,47^ but this chelator is also able to complex a series of other radiometals including ^177^Lu, ^86^Y, ^68^Ga, and ^111^In.^45,48,49^ Furthermore, an EDTA analog bearing a trans-1,2-diaminocyclohexane backbone (4-ICE) allows stable complexation of radiometals, such as ^111^In, ^57^Co, and ^47^Sc, but produced a less stable complexation of ^67^Cu as seen by a higher activity uptake in the liver of a respective antibody conjugate compared to conjugates bearing other chelators.^50^ In this context, it is known that a strong binding of copper ions is favored by macrocyclic ligands due to the so-called macrocyclic effect,^51^ which adds to the above-mentioned HSAB concept for explaining the low kinetic inertness of the [^64^Cu]Cu-CHDT complex.

### Characterization of PSMA Binding

**Figure 2:**
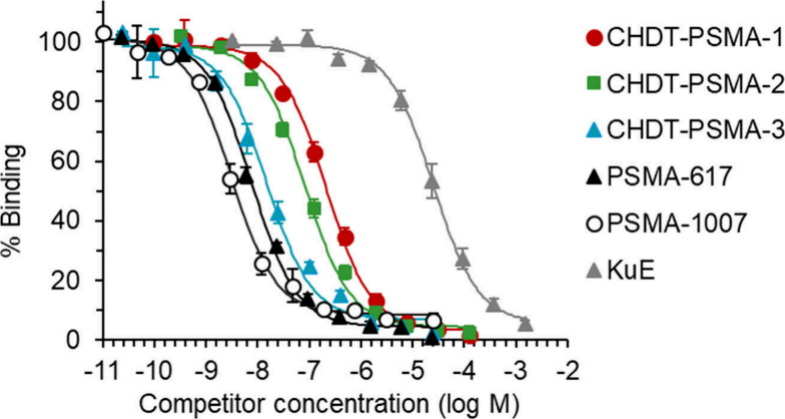
Competition binding of **CHDT-PSMA-1/2/3** compared to known PSMA ligands using LNCaP cell homogenates [^177^Lu]Lu-PSMA-617 was used as radioligand (12 nM). Data shown are mean values (±SEM) of 2–8 separate experiments (see Experimental Section for details and Table S2 in the Supporting Information for the corresponding data), each performed in triplicate or quadruplicate.

Motivated by the stability results for Al^18^F, ^68^Ga, and ^111^In-labeled **CHDT-PSMA-1/2/3**, we envisaged their further radiopharmacological characterization. First, the binding affinities of the novel compounds and three reference ligands (**PSMA-1007**, **PSMA-617**, and **KuE** (lysine-urea-glutamate, see Chemistry section in Supporting Information)) to PSMA were determined in a competition binding assay using LNCaP cell homogenates and **[**^**177**^**Lu]Lu-PSMA-617** as radioligand (Figure 2 and Table 2). In this context, LNCaP cells are known for their high amount of PSMA,^52^ which was also herein confirmed by Western blotting (Figure S6 in Supporting Information). While **PSMA-1007**, **PSMA-617**, and **CHDT-PSMA-3** show binding affinities by means of *K*_i_ values in the single-digit nanomolar range, **CHDT-PSMA-1/2** exhibit significantly lower affinities (49 and 115 nM). The comparable binding affinity of **CHDT-PSMA-3** and **PSMA-617** appears reasonable due to the high structure similarity, in particular the l-2-NaI and AMCH moieties. Similarly, the lower binding affinity of **CHDT-PSMA-2** can be rationalized due to the unfavorable orientation of the naphthyl moiety in 1-NaI within the PSMA binding site,^42^ which is obviously independent of the configuration (l or d) of the 1-naphthylalanine residue. **CHDT-PSMA-1** exhibits an even lower binding affinity than **CHDT-PSMA-2** indicating that the attachment of the CHDT moiety close to the KuE binding motif is detrimental to the binding to PSMA. In this context, although other PSMA ligands with a chelator moiety close to the KuE binding motif and a high binding affinity are known, e.g., PSMA-11 (6-aminohexanoyl linker between KuE and HBED-CC), the binding affinity seems to depend on the identity of the chelator moiety (*K*_i_ values of 12 and 37.6 nM for PSMA-11 and its DOTA analog, respectively).^53^ The lowest binding affinity was observed for the unmodified lysine-urea-motif (KuE), which agrees with the original report for this PSMA binding motif by Maresca *et al*.^54^

**Table 2:** *K*_i_ Values of **CHDT-PSMA-1/2/3** Compared to Known PSMA Ligands Determined from Competition Binding Assays^a^

	*K*_i_ (nM)
**CHDT-PSMA-1**	115 (13)
**CHDT-PSMA-2**	49 (8)
**CHDT-PSMA-3**	8.1 (1.9)
**PSMA-617**	4.2 (0.4)
**PSMA-1007**	1.7 (0.3)
**KuE**	11,300 (2,900)

a Data shown are mean values (±SEM) from 2 to 8 separate experiments (see Table S3 in the Supporting Information for the corresponding data), each performed in triplicate or quadruplicate using LNCaP cell homogenates.

**Figure 3:**
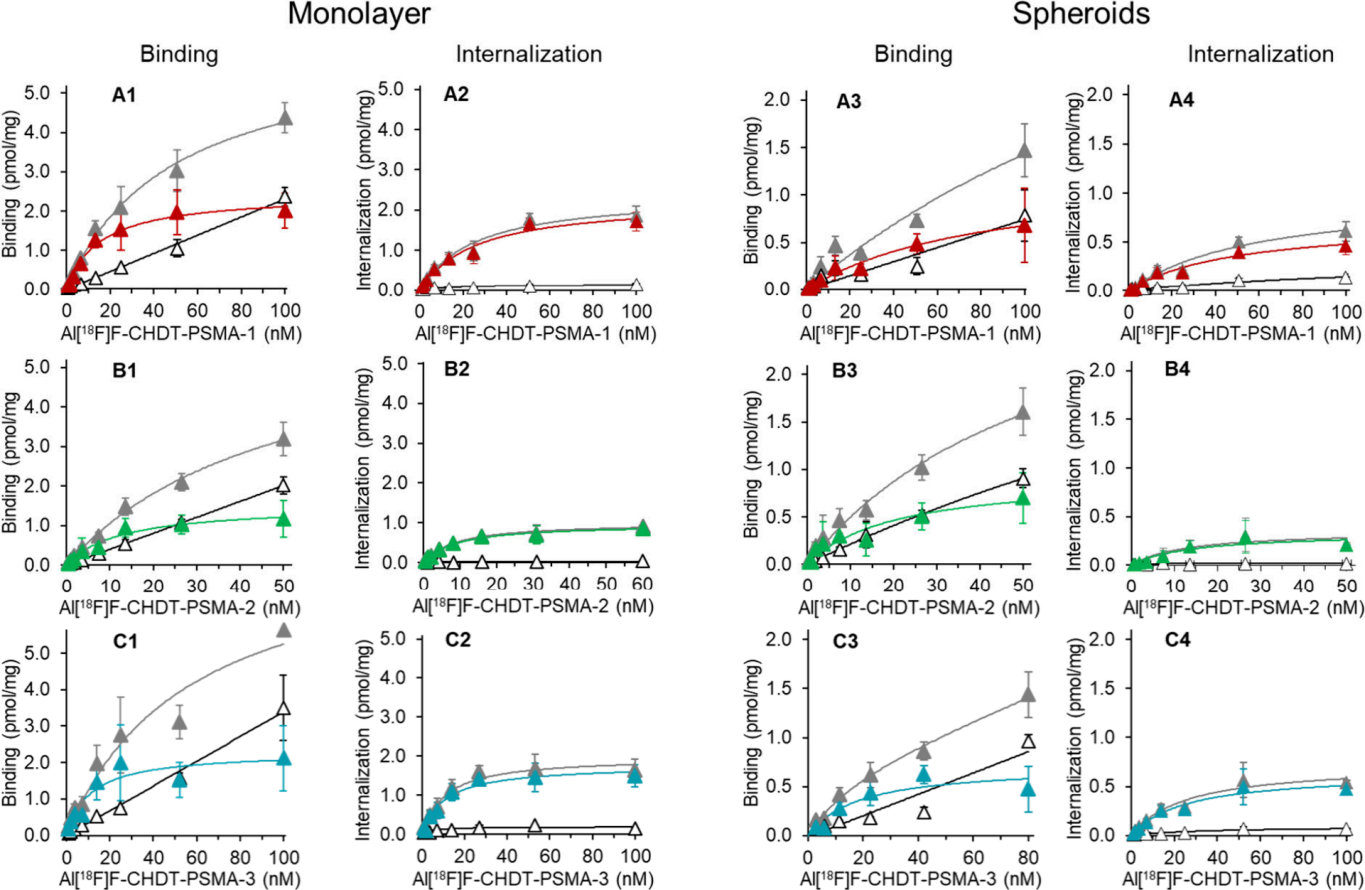
Exemplary saturation binding curves for **Al[^18^F]F-CHDT-PSMA-1/2/3** toward LNCaP cells extracellular saturation binding (“binding”) of Al[^18^F]F-CHDT-PSMA-1/2/3 toward intact LNCaP cells as monolayer (A1/B1/C1) and LNCaP spheroids (A3/B3/C3) and intracellular saturation binding (“internalization”) toward intact LNCaP cells (A2/B2/C2) and LNCaP spheroids (A4/B4/C4). Data for total (gray triangles), nonspecific (open triangles, in the presence of 800 μM KuE) and specific binding (**Al[^18^F]F-CHDT-PSMA-1** in red, **Al[^18^F]F-CHDT-PSMA-2** in green, and **Al[^18^F]F-CHDT-PSMA-3** in blue triangles) are shown as mean values (±SD) of one representative experiment, which was performed in duplicate (nonspecific binding) or triplicate (total binding). The corresponding data for these graphs are listed in Table S6 in the Supporting Information.

In addition to assessing the binding affinities for nonlabeled **CHDT-PSMA-1/2/3**, we sought to characterize the saturation binding of the Al^18^F-, ^68^Ga-, and ^111^In-labeled compounds using LNCaP cells cultured in monolayers and as spheroids. The saturation binding curves for **Al[**^**18**^**F]F-CHDT-PSMA-1/2/3** are depicted in Figure 3, while the graphs for **[**^**18**^**F]PSMA-1007** and ^68^Ga- and ^111^In-labeled **CHDT-PSMA-1/2/3** can be found in Figures S7–S9 in the Supporting Information. The binding parameters (*K*_d_ and *B*_max_) for **Al[**^**18**^**F]F-CHDT-PSMA-1/2/3** and **[**^**18**^**F]PSMA-1007** are summarized in Table 3 (for the ^68^Ga- and ^111^In-labeled compounds see Tables S4 and S5 in the Supporting Information). For the saturation binding assays, we recorded surface-bound radioligand (named “binding”), which refers to bound radioligand that can be removed by acid wash, and internalized or acid-resistant radioligand (named “internalization”), which refers to radioligand that remains cell-bound after acid wash. Consequently, two saturation binding plots were obtained and two sets of binding parameters (*K*_d_/*B*_max_ and *K*_d,int_/*B*_max,int_) were derived.

**Table 3:** Parameters for Extracellular and Intracellular Saturation Binding of **Al[^18^F]F-CHDT-PSMA-1/2/3** and **[^18^F]PSMA-1007**^a^

		Binding		Internalization	
Conjugate	LNCaP	*K*_d_ (nM)	*B*_max_ (pmol/mg)	*K*_d,int_ (nM)	*B*_max,int_ (pmol/mg)
**CHDT-PSMA-1**	M	26.4 ± 11.0	1.87 ± 0.57	18.4 ± 7.3	1.28 ± 0.96
	S	41.5 ± 26.4	1.49 ± 0.36	37.4 ± 10.7	0.49 ± 0.22
**CHDT-PSMA-2**	M	12.9 ± 4.0	1.33 ± 0.10	14.4 ± 4.8	0.79 ± 0.16
	S	15.8 ± 4.4	1.07 ± 0.13	15.7 ± 0.8	0.33 ± 0.15
**CHDT-PSMA-3**	M	19.7 ± 8.4	2.18 ± 0.97	14.4 ± 7.3	1.34 ± 0.32
	S	38.6 ± 25.8	0.90 ± 0.47	36.4 ± 14.6	0.86 ± 0.34
**PSMA-1007**	M	5.4 ± 3.6	1.11 ± 0.06	10.1 ± 5.9	1.02 ± 0.21
	S	12.9 ± 1.2	0.86 ± 0.57	25.8 ± 18.6	0.67 ± 0.32

a Data shown are mean values (±SEM) of 2–4 separate experiments (see Table S7 in the Supporting Information for the corresponding data). n.d. denotes not determined, M denotes monolayer, and S denotes spheroids.

Although the binding affinities of nonlabeled **CHDT-PSMA-1/2** were almost 1 order of magnitude lower than that of **CHDT-PSMA-3**, the *K*_d_ values (cell surface binding) of the radiolabeled analogs, independent of the particular radiolabel (Al^18^F, ^68^Ga, ^111^In), were comparable (factor < 4). For example, the *K*_d_ values for the Al^18^F-labeled compounds were 26.4, 12.9, and 19.7 nM, respectively, using LNCaP cells as monolayer. This might indicate in case of **CHDT-PSMA-1/2** that the CHDT moiety as complex with one of the above-mentioned radiometal species is better tolerated than the nonlabeled moiety. The most obvious difference after complexation is the overall charge of this moiety, with three negative charges due to the carboxyl groups being present in the nonlabeled state compared to an overall charge of −1 (Al^18^F) or ±0 (^68^Ga, ^111^In) after complexation. The longer linker entities between KuE binding motif and the CHDT moiety in **CHDT-PSMA-3** compared to the other two analogs might separate the CHDT moiety too far away from the PSMA binding site to exert any interactions with PSMA. Consequently, the complexation state of CHDT is not as crucial for the binding affinity as for the other two compounds. While there is no clear trend for the *K*_d_ values in dependence on the particular radiolabel, the *B*_max_ values were consistently higher by a factor of 2–3 for the Al^18^F-labeled compounds (1.33–2.18 pmol/mg) compared to the ^68^Ga- and ^111^In-labeled analogs (0.40–0.79 pmol/mg). In this context, **[**^**18**^**F]PSMA-1007** was also characterized in saturation binding experiments, which revealed a lower *K*_d_ value (5.4 nM) but a comparable *B*_max_ value (1.11 pmol/mg) compared to the Al^18^F-labeled **CHDT-PSMA-1/2/3**.

The results for the cell surface binding of the radioligands are largely conserved when analyzing the saturation binding data for the internalized fraction. This demonstrates that internalization is also concentration-dependent and that a constant fraction of around 50% of total-bound radioligand is internalized after 2 h (as *B*_max_ and *B*_max,int_ values are comparable). In contrast to the pronounced binding of all radioligands to LNCaP cells, binding to PSMA-negative PC3 cells was negligible (exemplarily shown for **Al[**^**18**^**F]F-CHDT-PSMA-1** in Figure S10 in Supporting Information), which further confirms their PSMA-specificity.

The results obtained for saturation binding using LNCaP cells cultured in monolayers are also conserved when using LNCaP spheroids. However, the *K*_d_ values were consistently higher and *B*_max_ values lower when comparing the data sets obtained for monolayer and spheroids. When the cells form a spheroid, the environment of the individual cells changes. An extracellular matrix is formed using adhesion molecules, and alterations regarding cell-to-cell communication and interaction, the orientation of cell structures, the ability of proliferation, aggregation, differentiation as well as the stiffness, oxygen, nutrient, and metabolic gradient occur.^55−57^ For LNCaP cells, abundance of PSMA was previously shown to be conserved in spheroids compared to monolayers.^58^ Therefore, regarding the somewhat reduced binding capacity toward spheroids observed herein originates most likely from a limited permeation potency of the radioligands into the spheroids and thus, the apparent amount of binding sites is reduced.

**Figure 4:**
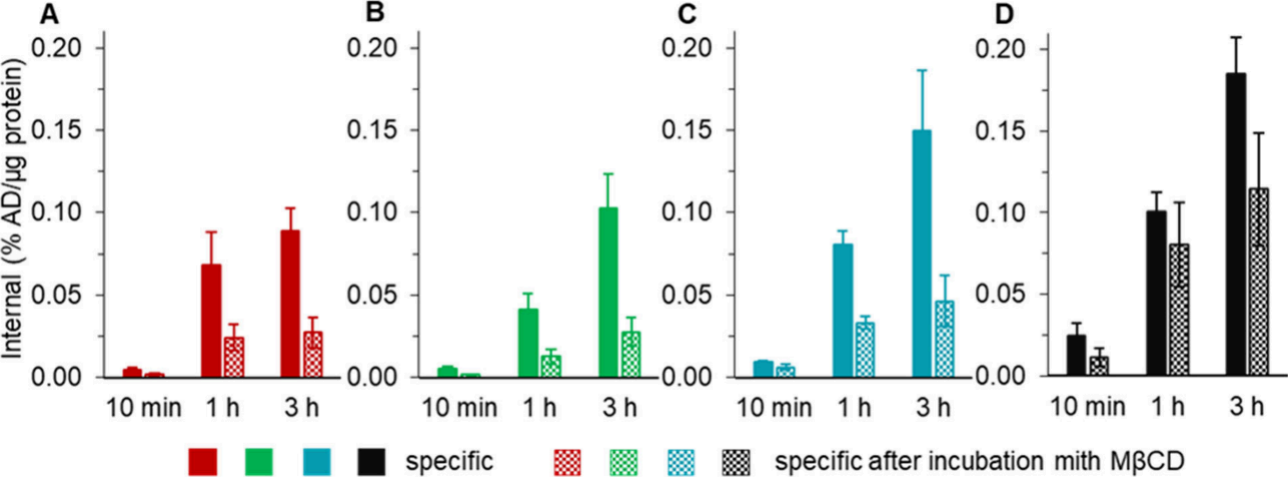
Internalization behavior of the Al^18^F-labeled **CHDT-PSMA-1/2/3** (A–C) and **[^18^F]PSMA-1007** (D) Time-depending internalization using LNCaP cells as monolayer with specific internalization and specific internalization in the presence of MβCD (3 mM). Data shown are mean values (±SEM) of 3 or 7 separate experiments (see Table S8 in the Supporting Information for the corresponding data), each performed in triplicate or quadruplicate. Same color coding as in Figure 3.

In addition to the saturation binding analyses of cell surface-bound and internalized radioligand, the time-dependent internalization of the radiolabeled **CHDT-PSMA-1/2/3** was investigated (10 min, 1 h, and 2 h). Specific internalization of the Al^18^F-labeled **CHDT-PSMA-1/2/3** and **[**^**18**^**F]PSMA-1007** are shown in Figure 4 (the data for the ^68^Ga- and ^111^In-labeled compounds are shown in Figure S11 in the Supporting Information). Internalization increased for all radiolabeled compounds over time with the highest values among the novel PSMA ligands obtained for **CHDT-PSMA-3**, irrespective of the particular radiolabel. The internalization of **Al[**^**18**^**F]F-CHDT-PSMA-3** was even comparable to that of **[**^**18**^**F]PSMA-1007**. In contrast, radiolabeled **CHDT-PSMA-1/2** exhibited a lower internalization. In agreement with the aforementioned high *B*_max_ values for the Al^18^F-labeled compounds, internalization was consistently higher for the Al^18^F-labeled **CHDT-PSMA-1/2/3** compared to their ^68^Ga- and ^111^In-labeled counterparts.

Regarding the mechanism of ligand induced PSMA internalization, it is known that this process occurs mainly via clathrin-coated pits.^59−61^ Previously, Matthias et al.^62^ demonstrated for fluorophore-labeled PSMA inhibitors and by using stimulated emission depletion (STED) nanoscopy that PSMA internalization upon inhibitor binding proceeds also via clathrin-dependent endocytosis. Herein, we also characterized the time-dependent internalization of the radiolabeled compounds in the presence of the methylated cyclic oligosaccharide methyl-β-cyclodextrin (MβCD),^63,64^ a known endocytosis inhibitor. Treatment with MβCD was associated with a significantly reduction in internalization for all PSMA radioligands examined herein, which indicates that the novel radioligands induce upon binding PSMA internalization via endocytosis.

### *Ex Vivo* Biodistribution and PET Imaging

Due to the original use of **(±)-H**_**3**_**RESCA** for complexation of (Al[^18^F]F)^2+^, we sought to subject the Al^18^F-labeled compounds developed herein bearing the novel CHDT moiety to a detailed *in vivo* radiopharmacological characterization. First, the biodistribution of the two radiolabeled model compounds, **Al[**^**18**^**F]F-CHDT-Pe** and **Al[**^**18**^**F]F-CHDT-Bn**, was studied at 60 and 240 min *p.i.* in healthy SKH1 mice to primarily assess the activity uptake in bone tissue and thus to get information about the *in vivo* stability of the **Al[^18^F]F-CHDT** complex (Tables S9 and S10 in the Supporting Information). While **Al[**^**18**^**F]F-CHDT-Pe** was mainly excreted into the urine (79% ID in urine at 60 min *p.i.*), the benzyl analog was predominantly excreted via the hepatobiliary route (51% ID in intestine at 60 min *p.i.*). This different *in vivo* behavior is reasonable considering the structures of both compounds with the aliphatic carboxylic acid moiety likely mediating the renal excretion pathway of **Al[**^**18**^**F]F-CHDT-Pe**. Activity uptake in bone tissue was assessed on the basis of the femur. Values of around 0.4%ID/g were measured, which did not increase up to 240 min *p.i.* This suggests a sufficient stability of Al[^18^F]F-CHDT complex *in vivo* regarding the release of [^18^F]F^–^ or related species that would accumulate permanently in the bone tissue.

**Figure 5:**
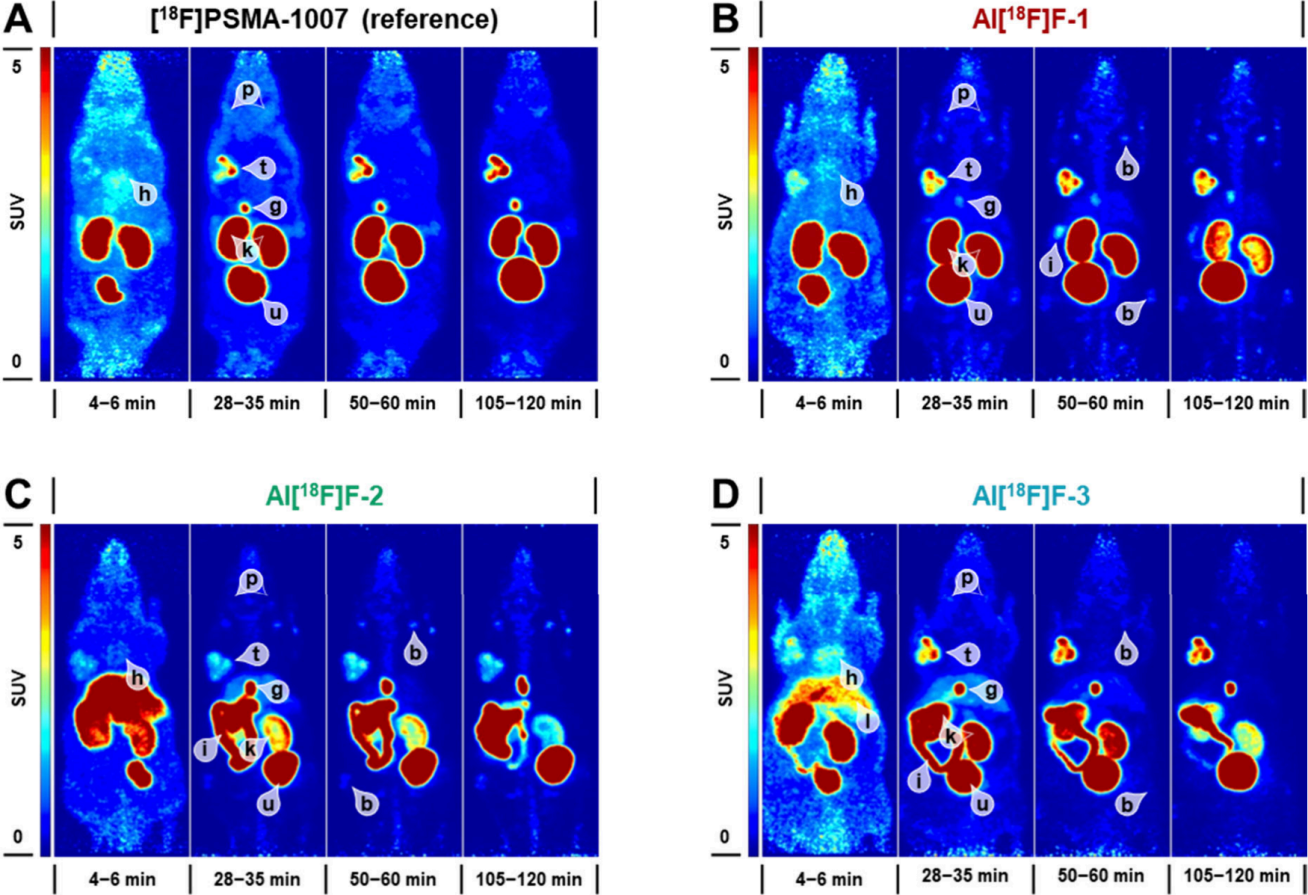
PET images of LNCaP xenograft mice after injection of Al^18^F-labeled CHDT-PSMA-1/2/3 compared to [^18^F]PSMA-1007 (A–D) Maximum intensity projections at specific time points; (b) bone, (g) gall bladder, (h) heart, (i) intestine, (k) kidneys, (l) liver, (p) parotid glands, (t) tumor, (u) urinary bladder; (SUV) standardized uptake value. Compounds names for the Al^18^F-labeled PSMA ligands were abbreviated.

**Figure 6:**
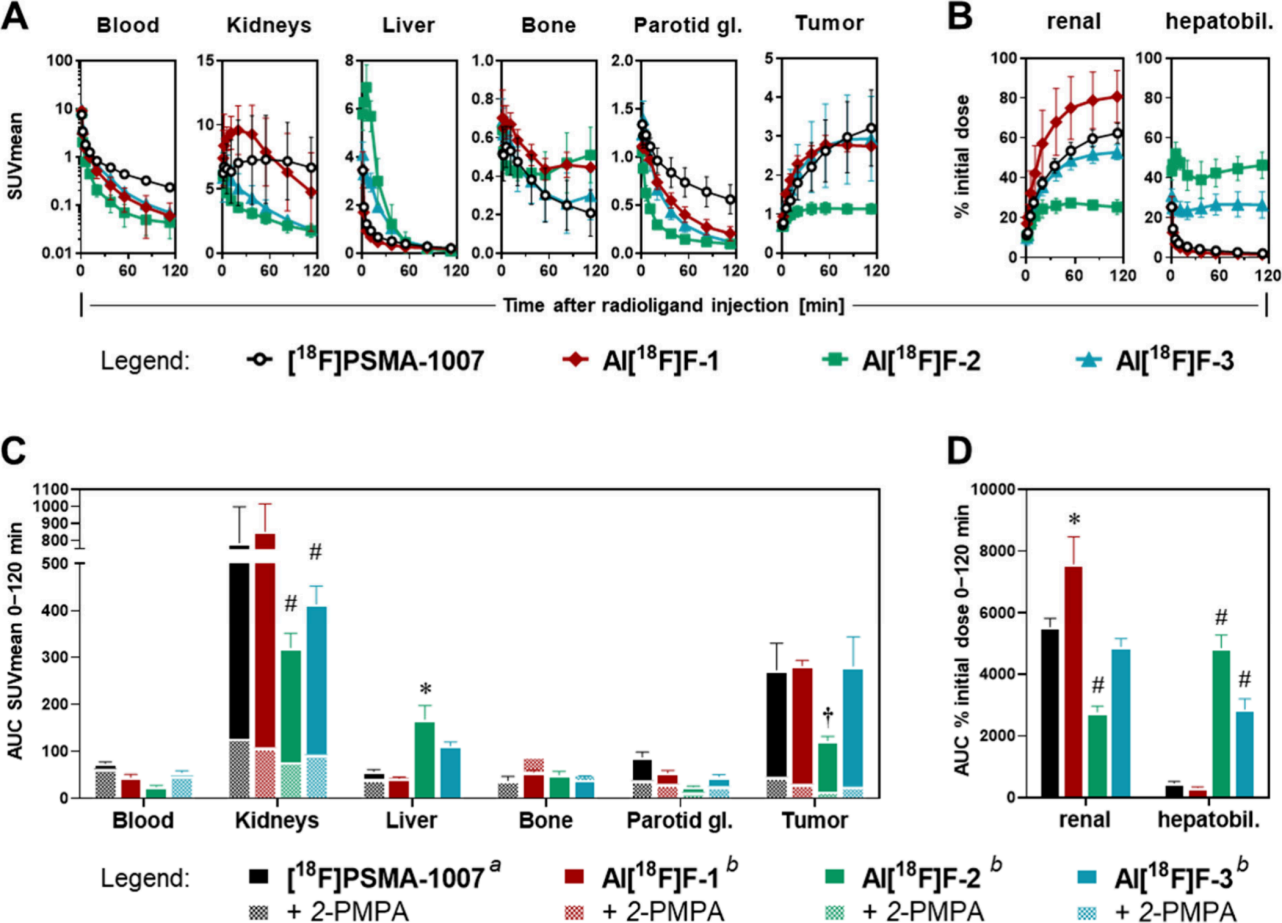
Pharmacokinetics of Al^18^F-labeled **CHDT-PSMA-1/2/3** compared to **[^18^F]PSMA-1007** data shown are from quantitative analysis of PET images. (A) time courses of activity concentrations in specific tissues (see Tables S11–S14 for the time-dependent SUVmean data); (B) time courses of excreted and/or retained activity fractions in renal (kidney + urinary bladder) and hepatobiliary (gall bladder + liver + intestine) organs; (C–D) areas under time-activity curves of specific tissues and excretory organs, (AUC) area under curve 0–120 min after radioligand injection, inhibition of PSMA-specific uptake with 2-PMPA administered simultaneously with the radioligand at a dose of 50 mg/kg; (SUV) standardized uptake value; mean values (^*a*^*n =*4, ^*b*^*n =*3, ± SD), statistical significance of differences compared to **[^18^F]PSMA-1007**: * *p* < 0.05; † *p* < 0.01; # *p* < 0.001. Compounds names for the Al^18^F-labeled PSMA ligands were abbreviated.

We furthermore investigated the biodistribution and suitability for targeting tumor-associated PSMA of Al^18^F-labeled **CHDT-PSMA-1/2/3** by PET imaging up to 120 min *p.i.* For this purpose, the PSMA-positive LNCaP tumor xenograft was used. Representative PET images at selected time points *p.i.* are shown in Figure 5, time-activity curves and total tissue uptakes based on PET images are illustrated in Figure 6. For comparison, [^**18**^**F]PSMA-1007** was also examined in this tumor model. For all radiofluorinated PSMA ligands, the LNCaP tumor was clearly visible and PSMA-specific uptake was confirmed by coadministration of 2-(phosphonomethyl)pentanedioic acid) (2-PMPA), which significantly reduced the tumor uptake (Figure 6C). Total tumor uptake was comparable for **Al[**^**18**^**F]F-CHDT-PSMA-1/3** and **[**^**18**^**F]PSMA-1007**, while uptake of **Al[**^**18**^**F]F-CHDT-PSMA-2** was significantly lower (Figure 6A and C). Considering rather comparable binding affinities of all novel Al^18^F-labeled PSMA ligands (Table 3), the inferior tumor targeting capability of **Al[**^**18**^**F]F-CHDT-PSMA-2** might primarily be a consequence of its altered pharmacokinetic behavior. **[**^**18**^**F]PSMA-1007** and **Al[**^**18**^**F]F-CHDT-PSMA-1/3** were mainly renally excreted, while **Al[**^**18**^**F]F-CHDT-PSMA-2** exhibited a higher uptake in the organs associated with hepatobiliary excretion compared to kidneys and bladder (Figure 6B and D). Apparently, the presence of the naphthylalanine residue shifts the excretion route to hepatobiliary excretion, which is partly compensated for **Al[**^**18**^**F]F-CHDT-PSMA-3** by the AMCH moiety. Although total uptake in bone tissue was low for all compounds (Figure 6C), the average SUVmean values at 120 min *p.i.* appeared to be slightly higher for the CHDT-functionalized PSMA ligands (0.30–0.51) compared to **[**^**18**^**F]PSMA-1007** (0.21, Figure 6A). This is in agreement with the results from *ex vivo* biodistribution studies of the two radiofluorinated model compounds and further supports our view that the Al[^18^F]F-CHDT complex is sufficiently stable i*n vivo*.

**Figure 7:**
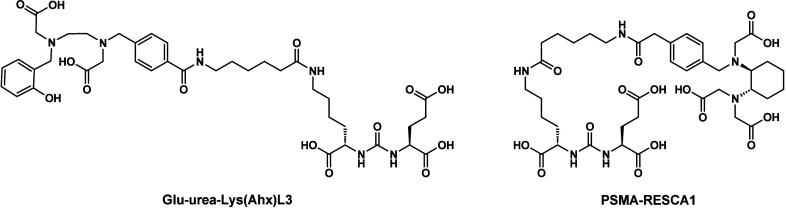
Structures of PSMA ligands Glu-urea-Lys(Ahx)L3^21,65^ and PSMA-RESCA1^65^

A more detailed view on the biodistribution and tumor uptake of **Al[**^**18**^**F]F-CHDT-PSMA-1** reveals that its *in vivo* performance is at least comparable to that of **[**^**18**^**F]PSMA-1007**. In fact, the tumor and kidney uptakes were similar but blood clearance was faster, which resulted in significantly higher tumor-to-blood and tumor-to-muscle ratios at 120 min *p.i.* (Table 4). The good *in vivo* performance of **Al[**^**18**^**F]F-CHDT-PSMA-1** is also striking considering the results of previously developed PSMA ligands by Cleeren *et al*. bearing acyclic pentadentate chelating units, i.e., **Glu-urea-Lys(Ahx)L3** and **PSMA-RESCA1** (Figure 7).^21,65^ A PSMA-specific tumor uptake was also seen for both Al^18^F-labeled ligands, but **Al[**^**18**^**F]F-PSMA-RESCA1** underlay a significant hepatobiliary excretion, which is less favorable for imaging purposes. Consequently, our objective of making the chelating unit more hydrophilic by the introduced triazole ring seems to be successful, although we should mention that the Ahx linker present in **PSMA-RESCA1** might also add a certain hydrophobicity. In contrast, **Al[**^**18**^**F]F-Glu-urea-Lys(Ahx)L3** showed a more pronounced uptake in bone tissue. Overall, our data for **Al[**^**18**^**F]F-CHDT-PSMA-1** indicate that this radioligand is an interesting candidate for clinical translation.

**Table 4:** Tumor-to-Organ Contrast in PET Imaging with Al^18^F-Labeled CHDT-**PSMA-1/2/3** Compared to **[^18^F]PSMA-1007**^a^

Radioligand	Tu/Blood	Tu/Kidney	Tu/Liver	Tu/Muscle	Tu/Bone
**[**^**18**^**F]PSMA-1007**^*i*^	14 ± 3.0	0.5 ± 0.2	16 ± 6.8	20 ± 6.9	18 ± 7.9
**Al[**^**18**^**F]F-CHDT-PSMA-1**^*ii*^	72 ± 44*	0.7 ± 0.4	15 ± 3.9	124 ± 65*	6.2 ± 0.6
**Al[**^**18**^**F]F-CHDT-PSMA-2**^*ii*^	32 ± 16	0.7 ± 0.3	22 ± 17	72 ± 32	2.3 ± 0.7*
**Al[**^**18**^**F]F-CHDT-PSMA-3**^*ii*^	43 ± 17	1.6 ± 0.5*	22 ± 14	116 ± 59	15 ± 11

a SUV mean ratios 105–120 min after radioligand injection; (Tu) tumor; mean values (^*i*^*n =* 4, ^*ii*^*n* = 3, ± SD); statistical significance of differences compared to **[**^**18**^**F]PSMA-1007**: * *p* < 0.05.

## Conclusion

Previously reported acyclic pentadentate chelating units including **(±)-H**_**3**_**RESCA** turned out to facilitate efficient Al^18^F-labeling at low temperatures with sufficient complex stabilities for *in vivo* applications. Based on **(±)-H**_**3**_**RESCA**, our newly developed alkyne-functionalized building block allows the straightforward functionalization of biomolecules via click chemistry and simultaneously forms the actual complexing unit with a triazole ring (CHDT) upon conjugation. Apart from its conserved suitability to complex (Al[^18^F]F)^2+^, we showed that the CHDT moiety also enables an efficient complexation of [^68^Ga]Ga^3+^ and [^111^In]In^3+^ and that the respective complexes exhibit a high kinetic inertness at least under physiological conditions *in vitro*. Furthermore, the preclinical characterization of the Al^18^F-labeled CHDT-functionalized PSMA ligands revealed that the triazole ring might also positively affect the *in vivo* behavior of small molecule or peptide-derived conjugates. In particular, the *in vivo* performance of **Al[**^**18**^**F]F-CHDT-PSMA-1** is even comparable to that of **[**^**18**^**F]PSMA-1007** rendering this novel PSMA ligand a promising candidate for clinical translation. Moreover, the activity uptake in bone tissue was really low and highlights the high stability of the Al[^18^F]F-CHDT-complex *in vivo*. For the [^68^Ga]Ga- and [^111^In]In-CHDT-complexes, the kinetic inertness *in vivo* needs to be further investigated. Overall, our results highlight that the excellent complexing properties of **(±)-H**_**3**_**RESCA** for (Al[^18^F]F)^2+^ can be further modified by chemical modifications to enable the efficient complexation of other radiometals and to improve the pharmacokinetics of respective biomolecule conjugates, which might pave the way for broadening the radiopharmaceutical use of this chelator type.

## Experimental Section

### General

For the syntheses of the PSMA conjugates and model compounds all chemicals were obtained by commercial suppliers and used without further purification. Solvents were obtained by Fisher Scientific and anhydrous solvents were supplied by Sigma-Aldrich. **PSMA-617** was purchased from ABX (Germany). **KuE** was prepared as previously described.^66,67^ Nuclear magnetic resonance spectra were recorded on an Agilent Technologies 400 MR spectrometer consisting of 400/54 premium compact magnet, 400 MR console and 400 MHz OneNMRProbe PT probe head (400 MHz for ^1^H, 101 MHz for ^13^C and 376 MHz for ^19^F). Spectra were processed by using the program MestreNova (version 14.2.1-27684). NMR chemical shifts were referenced to the residual solvent resonances relative to tetramethylsilane (TMS; ^1^H and ^13^C) and trichlorofluoromethane (CFCl_3_; ^19^F). Mass spectra (ESI) were obtained on a Waters Xevo TQ-S mass spectrometer (driven by the Mass Lynx software) or an Advion ExpressIon CMS spectrometer. The following molar masses of the final compounds were used for calculations (all molar masses were calculated including two TFA molecules): CHDT-Pe (697.53 g/mol), CHDT-Bn (687.54 g/mol), CHDT-PSMA-1 (956.75 g/mol), CHDT-PSMA-2 (1210.10 g/mol, CHDT-PSMA-3 (1335.27 g/mol).

### Chromatography

Thin-layer chromatography (TLC) was performed on Merck silica gel F-254 aluminum plates with visualization under UV (254 nm). Preparative column chromatography was carried out on the Flash Chromatography “Isolera Four” from Biotage using appropriate “Sfär” columns (SNAP HC-Sfär; 5, 10, or 25 g, depending on amount of crude product and solvent mixtures). Analytical RP-HPLC was performed on a VWR Hitachi system using an analytical Zorbax 300SB-C18 column, 100 × 4.6 mm (Agilent Technologies) and CH_3_CN/water (0.1% TFA) as mobile phase and a flow rate of 1 mL/min. Purification by RP-HPLC was performed on a semipreparative HPLC system (AlphaCrom, Rheinfelden, Switzerland) equipped with 2 × pumps (Varian, PrepStar 218 Solvent Delivery Module) and a UV/vis detector (Varian, ProStar 325, wavelength of detection 254 nm). A Microsorb C18 60-8 column (Viarian Dynamax 250 × 21.4 mm) was used as stationary phase and a binary gradient system of 0.1% CF_3_COOH/water (solvent A) and 0.1%CF_3_COOH/CH_3_CN at a flow rate of 10 mL/min served as the eluent. For UPLC-DAD-MS, a system from Waters (ACQUITY UPLC I class system including an ACQUITY UPLC PDA e λ detector coupled to a Xevo TQ-S mass spectrometer) was used. An ACQUITY UPLC BEH C18 column (1.7 μm, 130 Å, 100 × 2.1 mm, equipped with a ACQUITY UPLC BEH C18 VanGuard Precolumn, 1.7 μm, 130 Å, 5 × 2.1 mm) was used as stationary phase. A binary gradient system of 0.1% CH_3_COOH/water (solvent A) and 0.1% CH_3_COOH in CH_3_CN/CH_3_OH (1:1, v/v, solvent B) at a flow rate of 0.4 mL/min served as the eluent.

Analytical radio-HPLC was performed using a system from Knauer (Knauer Smartline Gabi Advanced Scientific Instruments, Smartline Pump 1000, UV-detector 2500, Manager 5000, Software: EZChrom Elite, Client/Server Version 3.2.7) with a Jupiter C-18 column (5 μm, 300 Å, 4.6 × 250 mm, Phenomenex, USA) as stationary phase. For the ^18^F-, ^68^Ga-, and ^111^In-labeled **CHDT-PSMA-1/2/3**, the following binary gradient system of water with 0.1% acetic acid (solvent A) and acetonitrile with 0.1% acetic acid (solvent B) was used: 0 to 3 min 95% A, 3 to 20 min 95% A to 5% A, 20 to 25 min 5% A, 25 to 28 min 5% A to 95% A, 28 to 37 min 95% A, flow rate 1 mL/min. For **[**^**177**^**Lu]Lu-PSMA-617**, the following binary gradient system of water with 0.1% TFA (solvent A) and acetonitrile with 0.1% TFA (solvent B) was used: 0 to 4 min 95% A, 4 to 15 min 95% A to 5% A, 15 to 20 min 5% A, 20 to 25 min 5% A to 95% A, 25 to 30 min 95% A, flow rate 1 mL/min.^68^ For radio-TLC, iTLC-SG strips (Agilent Technologies, USA) were used. An aliquot (0.3 μL, 1 μL for stability studies in human serum) of the respective radiolabeled compound was applied to the strip. The eluent was 2 M ammonium acetate/methanol (1:1, v/v). At the end, the stripes were exposed to a phosphor image plate (Fuji) and the scanned images (BAS 3000, Fuji, Raytest, Germany) were evaluated by the image analyzer program AIDA (Version 5.1 SP 4, Raytest, Germany).

### Radiolabeling

[^18^F]fluoride (no-carrier-added) was produced on a cyclotron (30 MeV TR-Flex-Cyclotron, Advanced Cyclotron Systems Inc., Canada) by irradiation of [^18^O]H_2_O via the ^18^O(p,n)^18^F nuclear reaction. An aliquot of the aqueous [^18^F]fluoride solution (600 MBq) was withdrawn and sodium acetate buffer (0.1 M, pH 4.0) was added to reach a volume of 300 μL. For formation of (Al[^18^F]F)^2+^, 10 μL of AlCl_3_ (2 mM, in 0.1 M sodium acetate, pH 4) was added and the mixture was incubated for 10 min at 22 °C. Subsequently, 10 μL of the respective CHDT-functionalized compound (stock of 1 μg/μL) were added and the mixture was incubated for 20 min at 40 °C (300 rpm, Thermomixer Comfort, Eppendorf, Germany). For processing after successful labeling, a few crumbs of hydroxyapatite were added to the mixture. After 30 s, the suspension was shortly centrifuged (10 s at 10000*g*, Eppendorf 5415R), and the supernatant was transferred to a clean vial. **[**^**18**^**F]PSMA-1007** was prepared according to a protocol from Cardinale *et al*.^69^

^68^Ga was eluted as [^68^Ga]GaCl_3_ from a ^68^Ge/^68^Ga generator of iThemba Laboratories (South Africa) in 0.6 M HCl. To 300 MBq of [^68^Ga]GaCl_3_, 1 M MES buffer (pH 6.0) was added until a pH of 5 to 6 was reached. Subsequently, 10 μL of the respective CHDT-functionalized compound (stock of 1 μg/μL) were added and the mixture was incubated for 20 min at 40 °C.

^111^In was purchased as [^111^In]InCl_3_ from Curium (United Kingdom). To 50 MBq of [^111^In]InCl_3_, 390 μL MES buffer (0.1 M, pH 5.5) and 10 μL of the respective CHDT-functionalized compound (stock of 1 μg/μL) were added and the mixture was incubated for 20 min at 40 °C.

[^177^Lu]LuCl_3_ was purchased from Isotope Technologies Munich AG (Germany). To 60 MBq of [^177^Lu]LuCl_3_, 90 μL MES buffer (0.1 M, pH 5.0) and 10 μL of **PSMA-617** (stock of 1 μg/μL) were added and the mixture was incubated for 20 min at 90 °C.

[^64^Cu]CuCl_2_ was produced on the 30 MeV TR-Flex-Cyclotron (Advanced Cyclotron Systems, Inc., ACSI, Canada) by ^64^Ni(p,n)^64^Cu nuclear reaction as reported previously.^70,71^ To 12 MBq of [^64^Cu]CuCl_3_, 0.1 M MES buffer (pH 5.5) was added until a pH of 5 to 6 was reached. Subsequently, 10 μL of the respective CHDT-functionalized compound (stock of 1 μg/μL) were added and the mixture was incubated for 30 min at 50 °C.

### *n*-Octanol/PBS Distribution Coefficient (logD_7.4_ Value)

The log D_7.4_ values were determined as follows: 500 μL of *n*-octanol and 450 μL of PBS (pH 7.4) were premixed prior to the addition of 50 μL of the respective radiolabeled compound. The mixture was vigorously stirred for 30 min at 22 °C, and thereafter centrifuged for 5 min at 7,500*g* at 22 °C. Aliquots of the n-octanol and the aqueous phases were transferred to measuring tubes, and the activity counts in both phases were measured in a gamma counter (Hidex Deutschland Vertrieb GmbH, Germany). The logarithm of the quotient of the activity counts from the *n*-octanol and the aqueous phase was calculated, which is equal to the log D_7.4_ value.

### Serum Stability Assay

To investigate the serum stability of the radiolabeled **CHDTA-PSMA-1/2/3**, human serum (frozen, from male AB clotted whole blood; Sigma-Aldrich/Merck) was centrifuged for 5 min at 20,000*g* at 4 °C after thawing. The supernatant was sterile filtrated (filter pore 0.2 μm). Four parts serum and one part of radioligand were incubated at 37 °C with shaking. Aliquots were taken after 3 h and for the ^111^In-labeled compounds also after 24 and 48 h, and residual intact radiolabeled compound was determined by radio-TLC and/or radio-HPLC. For analysis by radio-HPLC, the aliquots were mixed with Supersol (mixture for protein precipitation that consists of 20% ethanol, 0.5% Triton X-100, 5 mM EDTA and 0.1% saponin)^72^ and kept on ice for 5 min. After centrifugation for 5 min at 20,000*g* at 4 °C, the supernatant was used for radio-HPLC analysis.

### Biological *In Vitro* Assays

Binding assays were conducted using the high PSMA synthesizing human prostate carcinoma cell line LNCaP (ATCC CRL-1740) and the PSMA negative cell line PC3 (ATCC CRL-1435). The cells were grown as monolayer at 37 °C in a humidified atmosphere comprising 5% CO_2_ and 95% air in RPMI medium including 10% FCS (Biochrom AG). After washing the confluent cells twice with PBS and detaching them with trypsin/EDTA (0.05%/0.02%), the cells were suspended in RPMI and counted (Casy TT, Omni Life Science, Germany).

For the formation of spheroids, the LNCaP cells were incubated overnight in a normal culture bottle with biocompatible nanoparticles (1 × 10^–6^ cells with 80 μL of a suspension consisting of Au, Fe_2_O_3_ and poly-l-lysine called Nanoshuttle, Greiner Bio-One GmbH, Germany). After harvesting and cell counting, 10,000 cells/well of a 24-well plate or 7,000 cells/well of a 96-well plate (cell-repellent plates) were sown. The well plates were placed on matching plates with button magnets (Greiner Bio-One GmbH, Germany). The magnetized LNCaP cells formed suitable spheroids that were used after 5 d.

In order to assess the PSMA synthesis of LNCaP and PC3 cells, Western blotting was carried out. Cell lysates were prepared with RIPA buffer and 10 μg (protein level determined by Bio-Rad protein assay) was separated by SDS-PAGE (12% separating gel). This was followed by blotting onto a polyvinylidene fluoride membrane and blocking with 5% bovine serum albumin (BSA) in phosphate-buffered saline +0.1% Tween 20 (PBST) for 1 h, followed by an 1 h-incubation with appropriate primary antibodies (rabbit anti-PSMA 1:5,000; rabbit anti-β-actin 1:5,000, Cell Signaling Technology, USA) diluted with 5% BSA in PBST. After washing three times in PBST (à 5 min), the blots were incubated with a horseradish peroxidase (HRP)-conjugated secondary antibody (antirabbit-HRP 1:20,000, Cell signaling Technology Inc., Danvers, USA) for 1 h in 5% BSA-TBST. The visualization of the HRPenzyme activity was accomplished with Western blotting luminol reagent according to manufacturer’s instructions (Santa Cruz Biotechnology, Dallas, TX, USA). Enhanced chemiluminescence was detected with a blot scanner (C-DiGit Blot Scanner, LI-COR Bioscience GmbH, Germany).

#### Competition Binding Assay

The binding affinity of the nonlabeled **CHDT-PSMA-1/2/3**, **PSMA-1007**, **PSMA-617**, and **KuE** was assessed in competition binding assays using LNCaP cell homogenates (∼1 × 10^6^ cells/mL using Potter-Elvehjem) and the radioligand **[**^**177**^**Lu]Lu-PSMA-617** (*n* = 5 for **CHDT-PSMA-1**; *n* = 4 for **CHDT-PSMA-2/3**; *n* = 2 for **PSMA-1007**; *n* = 8 for **PSMA-617**; *n* = 6 for **KuE**). Homogenized cells were incubated with 40 μL of **[**^**177**^**Lu]Lu-PSMA-617** (final concentration 12 nM) in the presence of increasing concentrations of the compounds at 37 °C for 60 min: **CHDT-PSMA-1/2** (1.91 × 10^–9^ to 1.25 × 10^–4^ M), **CHDT-PSMA-3** (3.89 × 10^–10^ to 2.55 × 10^–5^ M), **PSMA-617** (9.15 × 10^–11^ to 1.20 × 10^–5^ M), and **KuE** (2.29 × 10^–8^ to 1.50 × 10^–3^ M). The assays were performed in a final volume of 200 μL. The incubation was stopped by washing the homogenate with cold PBS four times using a filter (Whatman GF/C, 90 min presoaked in 0.3% polyethylenimine) in a cell harvester (Brandel, USA). The radioactivity bound to the filter was measured in a gamma counter. Filter binding was determined for adjacent samples without cell homogenate. The experiments were performed two to four times in triplicates or quadruplicates. Inhibitory constants (*K*_i_) were derived from nonlinear regression according to the model of “One site – Fit Ki” as implemented in GraphPad Prism 10. The *K*_d_ value of **[**^**177**^**Lu]Lu-PSMA-617** was determined in saturation binding assays using LNCaP cell homogenates to be 15.2 (±2.1) nM.

#### Saturation and Internalization Assays

Saturation binding assays using cell homogenates of LNCaP cells (∼1 × 10^6^ cells/mL) were performed for **[**^**68**^**Ga]Ga-CHDT-PSMA-1**/**2** (0.2 to 50 nM) and using cell homogenates of PC3 cells (∼2 × 10^6^ cells/mL) for **Al[**^**18**^**F]F-CHDT-PSMA-1** (0.3 to 40 nM). To obtain the nonspecific binding adjacent samples received **KuE** (0.8 mM). The further procedure was carried out as described for the ’Competition Assay’ (see above).

For saturation assays in well plates, monolayers of LNCaP (sowing 5 × 10^4^ cells/well in 48-well plates 2 days before the assay), as well as LNCaP spheroids were incubated with radiolabeled **CHDT-PSMA-1/2/3** and **PSMA-1007** (0.2 nM to 100 nM) with adjacent samples receiving **KuE** (0.8 mM) at 37 °C for 2 h. The number of experiments can be derived from the data in the Tables S4, S5, and S7 in the Supporting Information. After removing the incubation medium and washing of cells with ice-cold PBS (containing Mg^2+^ and Ca^2+^) the surface-bound activity was stripped with 4 °C cold acid-wash buffer (0.2 M glycine, 0.15 M NaCl, pH 3.0) for 10 min. The acid wash buffer was transferred from the wells to measuring tubes, as was the PBS buffer after washing once. Cytosolic activity (internalized radioligand) was determined after treatment with cell lysis buffer (1% SDS in 0.1 M NaOH) at 37 °C for 30 min. Cell surface and cytosolic activity were measured separately in a gamma counter. Time-depending internalization assays were performed only with cells in monolayer. The incubation (radioligand concentration 13 nM) for these assays was terminated after 10 min, 1 and 3 h, with ^111^In and ^177^Lu additionally after 24 and 48 h at 37 °C, respectively. During the internalization assays adjacent samples received KuE (0.8 mM) for PSMA blocking, methyl-β-cyclodextrin (MβCD; 3 mM) or **KuE** together with MβCD, 1 h before radioligand application. The number of experiments can be derived from the data of Figure S11 and associated table and from Table S8 in the Supporting Information.

Dissociations constants (*K*_d_ and *K*_d,int_), as well as maximal binding capacities (*B*_max_ and *B*_max,int_), were derived from specific binding data by nonlinear regression according to the model of “One site-specific binding” as implemented in GraphPad Prism 10. Protein content was determined using the bicinchoninic acid protein assay kit (Pierce, Thermo Scientific) to calculate the binding capacities in pmol/mg and for the time-depending internalization the internalized percent of administrated dose per μg protein (% AD/μg protein).

### Small Animal PET/CT Imaging

#### Tumor Xenograft Model

All animal experiments were performed in accordance with the German Animal Welfare regulations and were approved by the local ethics committee for animal experiments (DD24.1-51-31/449/49, Oct 26, 2018). A PSMA-positive prostate cancer xenograft model was generated by subcutaneous injection of human LNCaP cells into the right shoulder of 8–12 week-old male nude mice (Rj:NMRI-*Foxn1*^*nu/nu*^, Janvier Laboratories, Le Genest-Saint-Isle, France). Imaging studies were performed when subcutaneous tumors reached a diameter of at least 6 mm. General anesthesia of the animals was induced and maintained by inhalation of 10% (v/v) desflurane in 30/70% (v/v) oxygen/air. Animals were continuously warmed at 37 °C during anesthesia.

#### Positron Emission Tomography

Positron emission tomography (PET) was performed *in vivo* using the nanoScan PET/CT scanner (Mediso Medical Imaging Systems, Budapest, Hungary), especially suited for small animals. Each animal received 8–12 MBq of the reference compound **[**^**18**^**F]PSMA-1007** or the radioligands **Al[**^**18**^**F]F-CHDT-PSMA-1/2/3** in phosphate-buffered saline by intravenous injection via a tail vein catheter. At the time of injection, molar activities and radiochemical purities of the compounds were as follows: **[**^**18**^**F]PSMA-1007** (15 MBq/nmol, >99%); **Al[**^**18**^**F]F-CHDT-PSMA-1** (10 MBq/nmol, 99%); **Al[**^**18**^**F]F-CHDT-PSMA-2** (6.3 MBq/nmol, 98%); **Al[**^**18**^**F]F-CHDT-PSMA-3** (4.5 MBq/nmol, >99%). Specificity of target binding was assessed by blocking PSMA binding sites with 2-phosphonomethyl pentanedioic acid (2-PMPA), administered simultaneously with the radioligand at a dose of 50 mg/kg. With injection of the radioligand, emission of the annihilation photons was recorded continuously in coincidence mode of 1:5 for 120 min. A corresponding CT image was acquired with each PET scan and used for attenuation correction and anatomical referencing. From three-dimensional list mode data, events within the energy window of 400–600 keV were extracted and sorted into 36 time frames (f1–f36) with a sequence of 15 × 10 s, 5 × 30 s, 5 × 60 s, 4 × 300 s, 3 × 600 s, and 4 × 900 s. For each time frame, a PET image was reconstructed with the three-dimensional (3D) Tera-Tomo algorithm, with a voxel size of 0.4 mm, applying corrections for random events, scattering, attenuation, and decay.

#### Quantitative Analysis of PET Images

PET images were analyzed using ROVER (ABX, Radeberg, Germany) and displayed as maximum intensity projections with identical intensity scaling. Three-dimensional regions of interest (ROIs) were defined within an image series of selected time frames using fixed intensity thresholds for delineation of organs: bones at knee joint position (0–120 min, 55%), cardiac blood volume (0–1.7 min, 70%), gall bladder (0–120 min, 39%), intestine (0–120 min, 5%), kidneys (0–2.5 min, 15%), liver (0–2.5 min, 55%), muscle (0–120 min, 0%), parotid glands (0–120 min, 39%), tumor (0–120 min, 39%), and urinary bladder (0–120 min, 5%). Activity concentrations in tissue were determined as standardized uptake value (SUV = [MBq detected activity/mL tissue]/[MBq injected activity/g body weight]) and expressed as SUVmean (ROI-averaged). Time-activity curves were generated and analyzed using Prism (GraphPad Software, San Diego CA, USA). Data points of the time-activity curves were generated by extracting the maximum value at <1.7 min followed by averaging the subsequent values in subgroups with summarized midframe times of 2.1, 5.5, 11, 20, 38, 55, 82, and 113 min. Areas under curves (AUC) were calculated using the trapezoid rule. Statistical significance of mean differences was tested by analysis of variance (ANOVA) using the Šidák test.

## Supporting Information Available

Exemplary radio-TLC and radio-HPLC chromatograms, radio-SDS-PAGE radioluminograms for the serum stability assays of **[**^**64**^**Cu]Cu-CHDT-PSMA-1** and **[**^**68**^**Ga]Ga-CHDT-PSMA-1**, Western Blot for PSMA-synthesis in LNCaP and PC3 cells, exemplary saturation binding curves of **[**^**18**^**F]PSMA-1007**, **[**^**68**^**Ga]Ga-CHDT-PSMA-1/2/3**, **[**^**111**^**In]In-CHDT-PSMA-1/2/3** toward LNCaP cells and of **Al[**^**18**^**F]F-CHDT-PSMA-1** toward PC3 cells, internalization data for **[**^**68**^**Ga]Ga-CHDT-PSMA-1/2/3**, **[**^**111**^**In]In-CHDT-PSMA-1/2/3** using LNCaP cells, individual data and mean values for the determination of logD_7.4_, as well as the competition binding, saturation binding, and internalization assays shown in the main article and the Supporting Information, *ex vivo* biodistribution data of **Al[**^**18**^**F]F-CHDT-Pe** and **Al[**^**18**^**F]F-CHDT-Bn**, SUV-time data for all PET experiments, and synthetic procedures and analytical data (chemistry) of all compounds (NMR, ESI-MS, HPLC) (PDF)

## Abbreviations Used

AMCH: *trans*-4-(aminomethyl)cyclohexanecarboxylic acid
CHDA: cyclohexanediamine alkyne
CHDT: cyclohexanediamine triazole
PSMA: prostate specific membrane antigen
RESCA: restrained complexing agent
SUV: standardized uptake value
