# Supplementary material for: Cyclohexanediamine Triazole (CHDT) Functionalization Enables Labeling of Target Molecules with Al18F/68Ga/111In
Source: Bioconjug Chem. 2024 Aug 26;35(9):1402–16. doi: 10.1021/acs.bioconjchem.4c00313 (PMC11417994; doi:10.1021/acs.bioconjchem.4c00313)
Supplement: Supplementary file 1 — bc4c00313_si_001.pdf [file bc4c00313_si_001.pdf]

Supporting Information

Cyclohexanediamine triazole (CHDT)-functionalization enables labeling of target molecules with $Al^{18}F$ / ^{68}Ga / ^{111}In

Wiebke Sihver[a,*], Martin Walther[a,#], Martin Ullrich[a,#], Anne-Kathrin Nitt-Weber[a], Jenny Böhme[a], Falco Reissig[a], Magdalena Saager[a], Kristof Zarschler[a], Christin Neuber[a], Jörg Steinbach[a], Klaus Kopka[a,b], Hans-Jürgen Pietzsch[a,b], Robert Wodtke[a], Jens Pietzsch[a,b]

[a] Helmholtz-Zentrum Dresden-Rossendorf, Institute of Radiopharmaceutical Cancer Research, Bautzner Landstraße 400, 01328 Dresden, Germany

[b] Technische Universität Dresden, School of Science, Faculty of Chemistry and Food Chemistry, Mommsenstraße 4, 01069 Dresden, Germany

[#]*These authors contributed equally to this work.*

*E-mail: w.sihver@hzdr.de

Table of Contents

Figure S1: Exemplary radio-TLCs for complexation of $(Al[^{18}F]F)^{2+}$, $[^{68}Ga]Ga^{2+}$ and $[^{64}Cu]Cu^{2+}$ by CHDT-Pe and of $(Al[^{18}F]F)^{2+}$ by CHDT-Bn......4

Figure S2: Exemplary radio-TLCs for complexation of $(Al[^{18}F]F)^{2+}$, $[^{68}Ga]Ga^{2+}$, $[^{64}Cu]Cu^{2+}$, and $[^{111}In]In^{3+}$ by CHDT-PSMA-1...............................5

Figure S3: Analytical radio-HPLC chromatograms of radiolabeled CHDT-PSMA-1/2/3 and of $[^{177}Lu]$Lu-PSMA-617 and $[^{18}F]$PSMA-1007...............6

Figure S4: Serum stability assay for $[^{64}Cu]$Cu-CHDT-PSMA-1 using human serum 7

Figure S5: Serum stability assay for $[^{68}Ga]$Ga-CHDT-PSMA-1 using human serum 8

Figure S6: Confirmation of PSMA synthesis on LNCaP cells by Western blotting..9

Figure S7: Exemplary saturation binding curves of $[^{18}F]$PSMA-1007 toward LNCaP cells..10

Figure S8: Exemplary saturation binding curves of $[^{68}Ga]$Ga-CHDT-PSMA-1/2/3 toward LNCaP cells...11

Figure S9: Exemplary saturation binding curves of $[^{111}In]$In-CHDT-PSMA-1/2/3 toward LNCaP cells...13

Figure S10: Exemplary saturation binding curves of $Al[^{18}F]$F-CHDT-PSMA-1 toward PC3 cells...16

Figure S11: Internalization behavior of the ^{68}Ga- and ^{111}In-labeled CHDT-PSMA-1/2/3 17

Table S1: Individual values for $logD_{7.4}$ and serum stability of the radiolabeled CHDT conjugated compounds 19

Table S2: Individual data (percent binding $[^{177}Lu]$Lu-PSMA-617) for each competition binding experiment 20

Table S3: Individual mean values for K_i determined from each competition binding experiment 22

Table S4: Parameters for extracellular and intracellular saturation binding of $[^{68}Ga]$Ga-CHDT-PSMA-1/2/3 23

Table S5: Parameters for extracellular and intracellular saturation binding of $[^{111}In]$In-CHDT-PSMA-1/2/3 24

Table S6: Data for the exemplary saturation binding curves of $Al[^{18}F]$F-CHDT-PSMA-1/2/3...25

Table S7: Individual mean values for K_d and B_{max} determined from each saturation binding experiment with $Al[^{18}F]$F-CHDT-PSMA-1/2/3 and $[^{18}F]$PSMA-1007...27

Table S8: Individual mean values for the internalization determined from each internalization experiment with $Al[^{18}F]$F-CHDT-PSMA-1/2/3 and $[^{18}F]$PSMA-1007...28

Table S9: Ex vivo biodistribution data for $Al[^{18}F]$F-CHDT-Pe in SKH1 mice29

Table S10: **Ex vivo biodistribution data for Al[^{18}F]F-CHDT-Bn in SKH1 mice 31**

Table S11: **Tissue-specific time-activity courses of [^{18}F]PSMA-1007 in LNCaP xenograft mice 33**

Table S12: **Tissue-specific time-activity courses of Al[^{18}F]F-CHDT-PSMA-1 in LNCaP xenograft mice 34**

Table S13: **Tissue-specific time-activity courses of Al[^{18}F]F-CHDT-PSMA-2 in LNCaP xenograft mice 35**

Table S14: **Tissue-specific time-activity courses of Al[^{18}F]F-CHDT-PSMA-3 in LNCaP xenograft mice 36**

Chemistry 37

Intermediate compounds....37

Compound 1....37

Compound 2....38

Compound 3....39

Compound 4....40

Compound 5....41

Compound 6....42

Compound 7....43

Compound 8....44

Compound 9 (2×TFA)....45

Compound 10....46

Compound 11....47

Final compounds....48

(±)-CHDA-*t*Bu....48

CHDT-Pe (×2TFA)....49

CHDT-Bn (×2TFA)....50

CHDT-PSMA-1 (×2TFA)....51

CHDT-PSMA-2 (×2TFA)....52

CHDT-PSMA-3 (×2TFA)....53

References 54

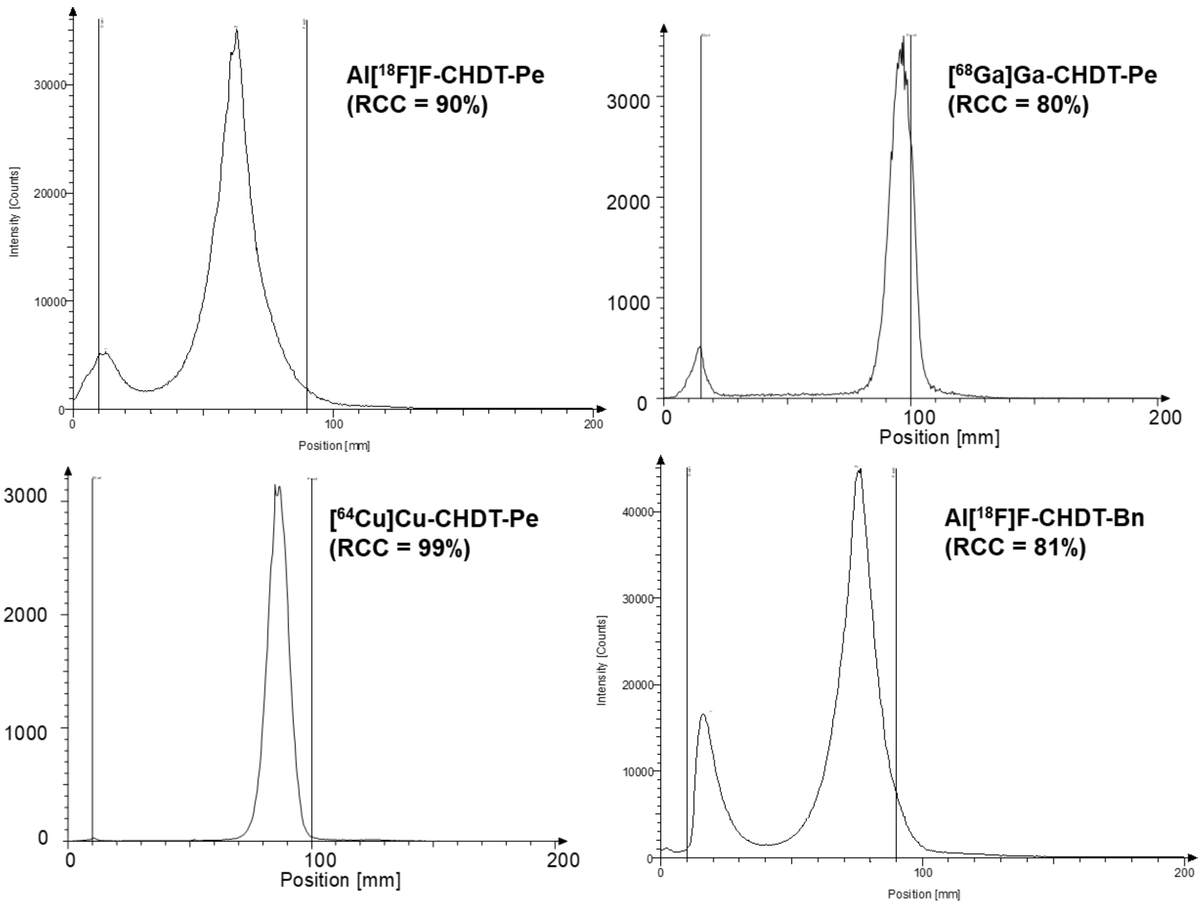

Figure S1: Exemplary radio-TLCs for complexation of $(Al[^{18}F]F)^{2+}$, $[^{68}Ga]Ga^{2+}$ and $[^{64}Cu]Cu^{2+}$ by CHDT-Pe and of $(Al[^{18}F]F)^{2+}$ by CHDT-Bn

---

Radiochemical conversions were assessed by radio-TLC (2 M NH_4OAc/methanol 25:75 (v/v) RP18 material). For all of the tested complexation reactions unreacted radiometal species remain at the start of the TLC stripes (R_f = 0), whereas the complexes exhibit R_f values around 0.7-1.0. Conditions for $Al^{18}F$-labeling were as follows: 0.2 M MES (pH 5.0, 200 µL), 2 mM $AlCl_3$ (5 µL), $[^{18}F]F^-$ (66 µL, 262 MBq, aqueous solution from irradiation), **CHDT-Pe** or **CHDT-Bn** (50 µL of 1 µg/µL stock, final 0.16 µg/µL), 20 min rt for formation of $(Al[^{18}F]F)^{2+}$ and 20 min rt for complexation. Similar conditions were applied for ^{68}Ga- and ^{64}Cu-labeling.

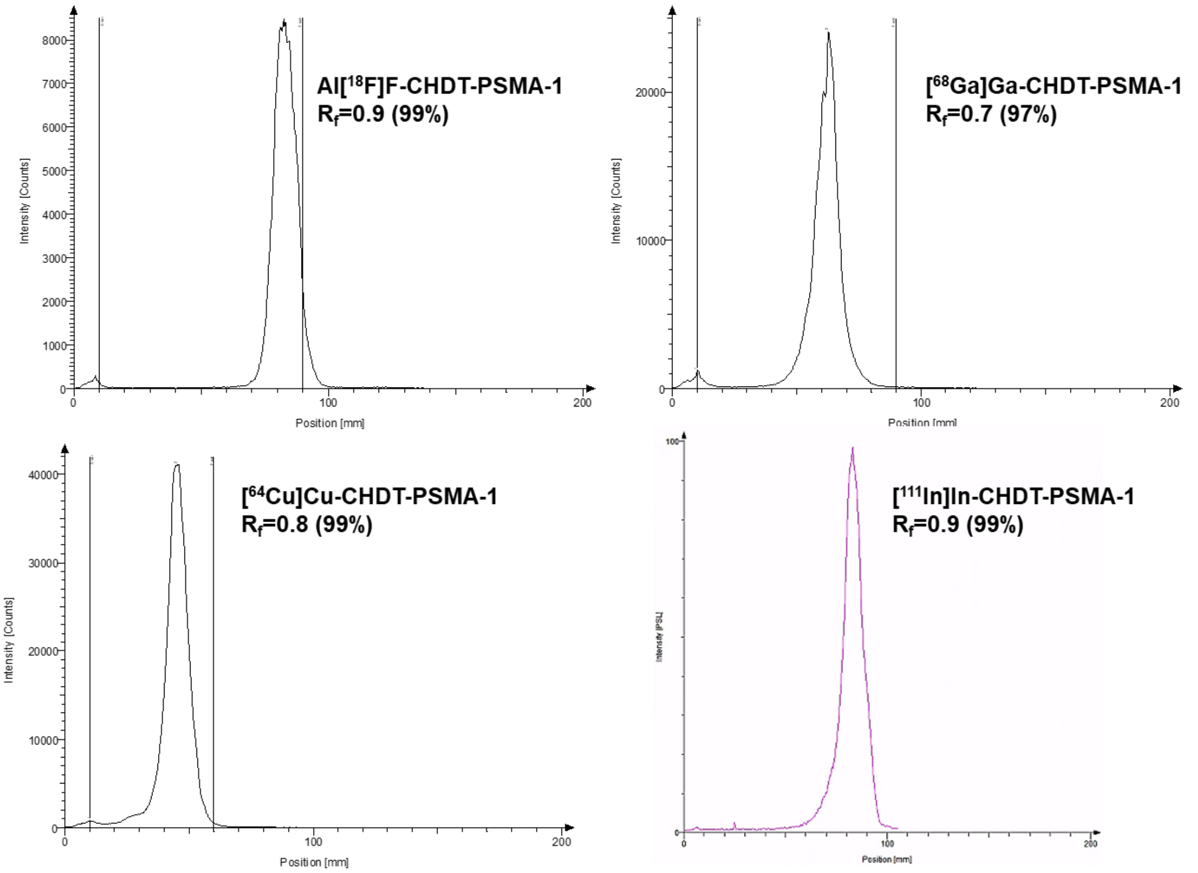

Figure S2: Exemplary radio-TLCs for complexation of (Al[^{18}F]F)$^{2+}$, [^{68}Ga]Ga^{2+}, [^{64}Cu]Cu^{2+}, and [^{111}In]In^{3+} by CHDT-PSMA-1

Radiochemical conversions were assessed by radio-TLC (2 M NH_4OAc/methanol 1:1 (v/v) RP18 material for **Al[^{18}F]F-CHDT-PSMA-1**; 2 M NH_4OAc/methanol 1:1 (v/v) ITLC-SG material for **[^{68}Ga]Ga-CHDT-PSMA-1** and **[^{111}In]In-CHDT-PSMA-1**; CH_3CN/water 1:1 (v/v) +0.1%TFA RP18 material for **[^{64}Cu]Cu-CHDT-PSMA-1**). For all of the tested complexation reactions unreacted radiometal species remain at the start of the TLC stripes (R_f = 0), whereas the complexes show R_f values around 0.7-1.0 under the specified conditions.

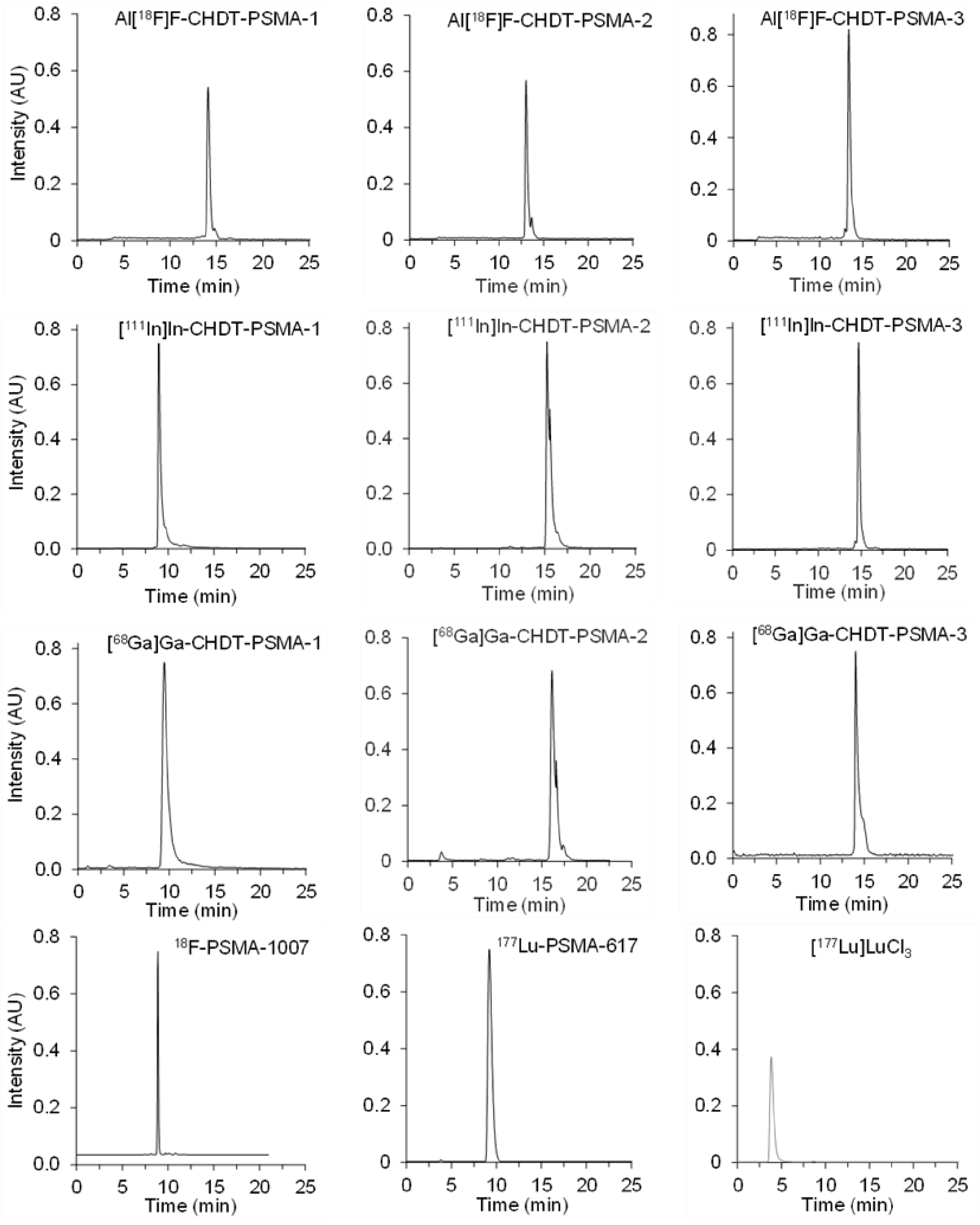

Figure S3: **Analytical radio-HPLC chromatograms of radiolabeled CHDT-PSMA-1/2/3 and of [^{177}Lu]Lu-PSMA-617 and [^{18}F]PSMA-1007**

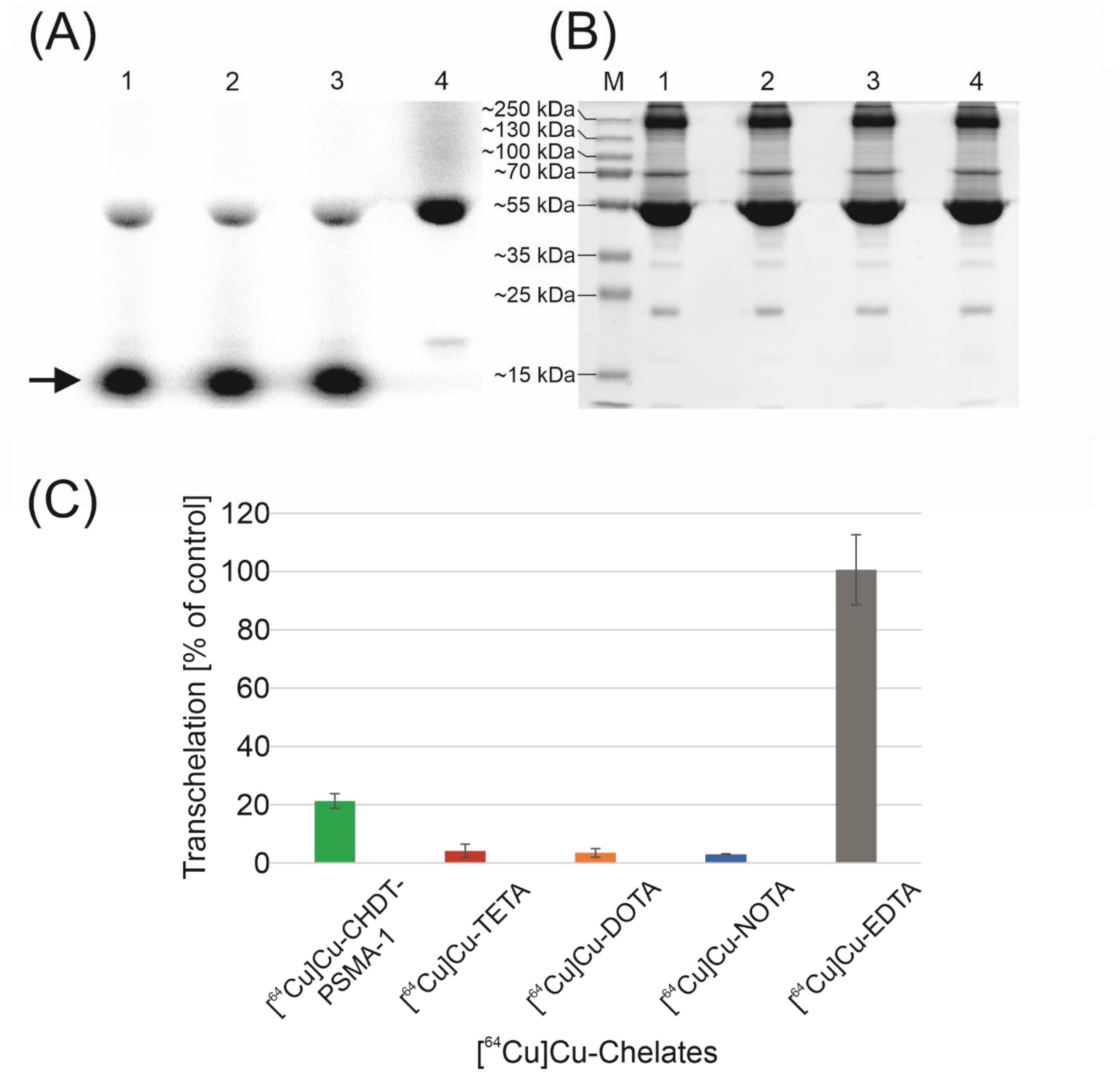

Figure S4: Serum stability assay for [^{64}Cu]Cu-CHDT-PSMA-1 using human serum

After radiolabeling, ^{64}Cu-species were incubated for 1 h at 37°C in human serum and subsequently separated by SDS-PAGE. Following electronic autoradiography (**A**), gels were stained with colloidal Coomassie staining solution (**B**): lane 1 to 3 triplicates of **[^{64}Cu]Cu-CHDT-PSMA-1**; lane 4 [^{64}Cu]$CuCl_2$. The bands corresponding to **[^{64}Cu]Cu-CHDT-PSMA-1** are highlighted by an arrow. The bands with a molecular weight of ~55 kDa represent human serum albumin. The comparative quantitative analysis of ^{64}Cu-transchelation (% of control) in human serum for ^{64}Cu-labeled chelates is shown in (**C**).

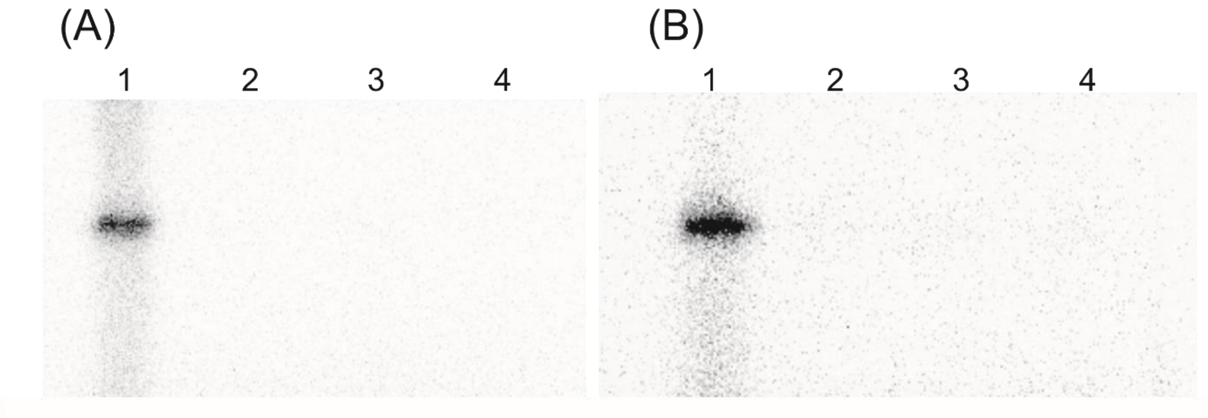

Figure S5: Serum stability assay for [^{68}Ga]Ga-CHDT-PSMA-1 using human serum

After radiolabeling, ^{68}Ga-species **[^{68}Ga]Ga-CHDT-PSMA-1** (**A**) or **[^{68}Ga]Ga-DOTA** (**B**), respectively, were incubated for 1 h at 37°C in human serum and subsequently separated by SDS-PAGE. Autoradiography showing ^{68}Ga-labeld bands of human serum proteins: lane 1 [^{68}Ga]$GaCl_3$ and lane 2 to 4 triplicates of **[^{68}Ga]Ga-CHDT-PSMA-1** (**A**) or **[^{68}Ga]Ga-DOTA** (**B**). The bands occurred at a molecular weight of ~76 kDa and represent human transferrin.

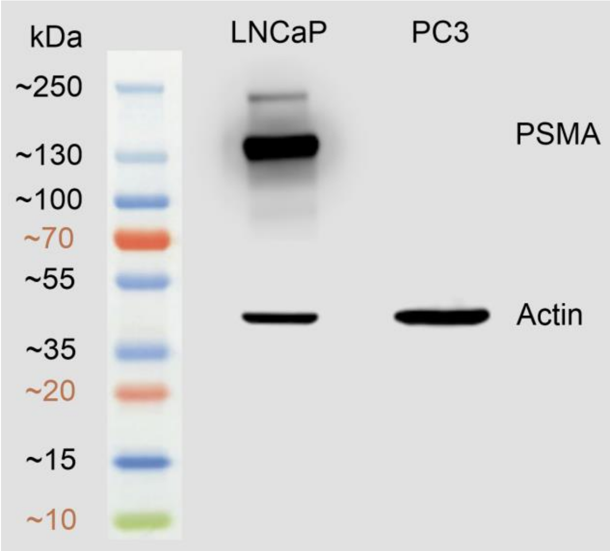

Figure S6: Confirmation of PSMA synthesis on LNCaP cells by Western blotting

Detection of endogenous PSMA expression in LNCaP and PC3 cells relative to β-actin expression by Western blotting

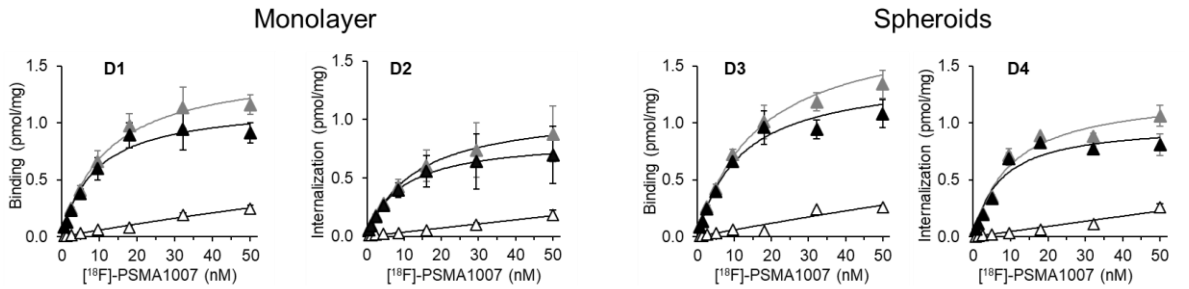

Figure S7: Exemplary saturation binding curves of [^{18}F]PSMA-1007 toward LNCaP cells

Extracellular saturation binding ("binding") of **[^{18}F]PSMA-1007** toward intact LNCaP cells as monolayer (**D1**) and spheroids (**D3**) as well as intracellular saturation binding ("internalization") toward intact LNCaP cells as monolayer (**D2**) and spheroids (**D4**). Data for total (grey triangles), nonspecific (open triangles, in the presence of 800 µM KuE) and specific binding (black triangles) are shown as mean values (± SD) of one representative experiment, which was performed in duplicate or triplicate. The corresponding data are shown in the tables below.

**D1 Binding LNCaP Monolayer**						
Conc (nM)	Total bg (pmol/mg)		Nonsp bg (pmol/mg)		Spec bg (pmol/mg)	
	mean	SD	mean	SD	T-Nsp	SD
**0.68**	0.086	0.003	0.004	0.000	0.082	0.003
**1.32**	0.132	0.005	0.007	0.000	0.125	0.005
**2.60**	0.247	0.014	0.015	0.000	0.232	0.014
**4.99**	0.407	0.041	0.031	0.007	0.376	0.041
**9.56**	0.658	0.098	0.060	0.002	0.598	0.098
**17.96**	0.971	0.111	0.080	0.016	0.891	0.112
**32.2**	1.132	0.181	0.190	0.018	0.942	0.182
**50.0**	1.159	0.087	0.248	0.028	0.911	0.091

**D2 Internalization LNCaP Monolayer**						
Conc (nM)	Total int (pmol/mg)		Nonsp int (pmol/mg)		Spec int (pmol/mg)	
	mean	SD	mean	SD	T-Nsp	SD
**0.61**	0.060	0.004	0.008	0.005	0.052	0.006
**1.19**	0.099	0.008	0.008	0.003	0.090	0.009
**2.34**	0.179	0.007	0.013	0.003	0.166	0.007
**4.45**	0.281	0.004	0.019	0.001	0.262	0.004
**8.47**	0.427	0.064	0.030	0.005	0.397	0.064
**16.02**	0.606	0.133	0.049	0.007	0.556	0.134
**29.4**	0.738	0.235	0.099	0.017	0.639	0.236
**50.0**	0.877	0.240	0.181	0.042	0.696	0.244

**D3 Binding LNCaP Spheroids**						
Conc (nM)	Total bg (pmol/mg)		Nonsp bg (pmol/mg)		Spec bg (pmol/mg)	
	mean	SD	mean	SD	T-Nsp	SD
**0.68**	0.089	0.003	0.004	0.000	0.084	0.003
**1.32**	0.138	0.007	0.009	0.000	0.130	0.007
**2.60**	0.263	0.016	0.019	0.001	0.244	0.016
**4.99**	0.423	0.034	0.029	0.001	0.394	0.034
**9.56**	0.723	0.042	0.061	0.002	0.663	0.042
**17.96**	1.008	0.148	0.050	0.002	0.958	0.148
**32.2**	1.184	0.082	0.223	0.018	0.962	0.084
**50.0**	1.340	0.122	0.260	0.010	1.079	0.123

**D4 Internalization LNCaP Spheroids**						
Conc (nM)	Total int (pmol/mg)		Nonsp int (pmol/mg)		Spec int (pmol/mg)	
	mean	SD	mean	SD	T-Nsp	SD
**0.68**	0.065	0.005	0.003	0.000	0.063	0.005
**1.32**	0.117	0.006	0.005	0.000	0.112	0.006
**2.60**	0.204	0.003	0.010	0.000	0.195	0.003
**4.99**	0.355	0.012	0.020	0.000	0.335	0.012
**9.56**	0.715	0.055	0.033	0.002	0.682	0.055
**17.96**	0.885	0.007	0.063	0.011	0.822	0.013
**32.2**	0.882	0.026	0.111	0.003	0.771	0.026
**50.0**	1.060	0.091	0.255	0.031	0.805	0.096

Monolayer

Spheroids

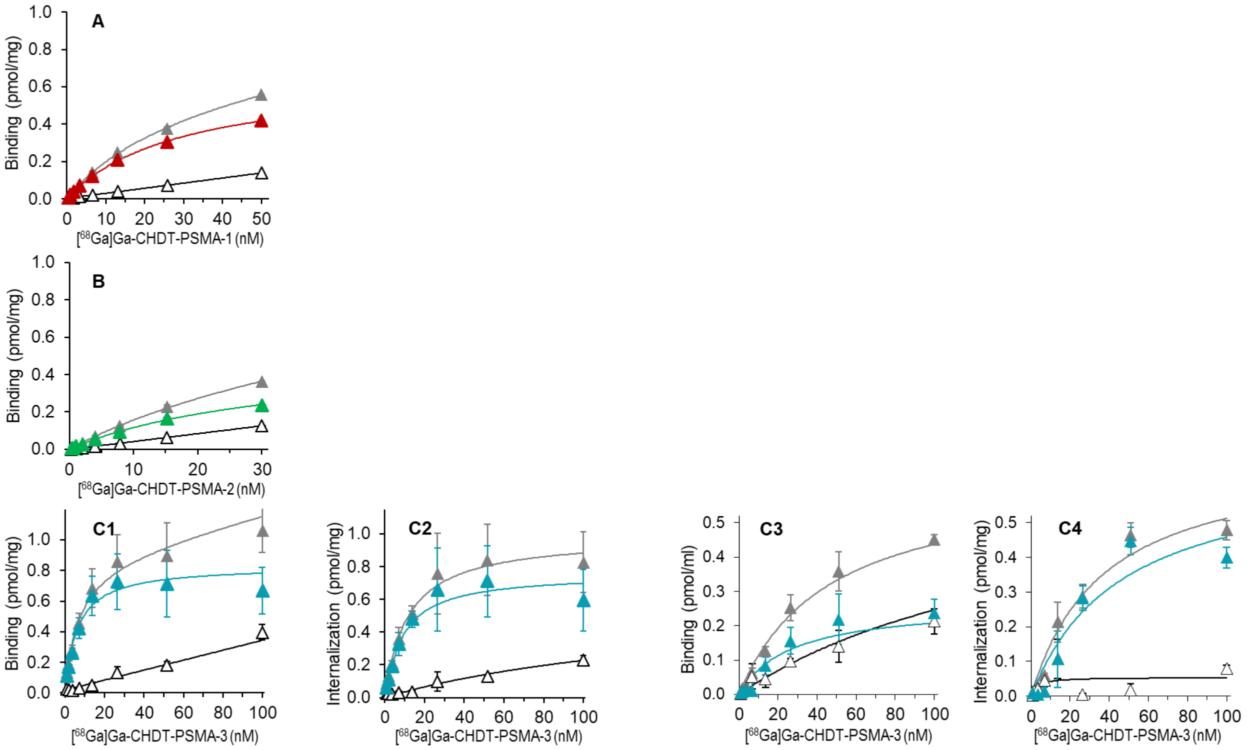

Figure S8: Exemplary saturation binding curves of [^{68}Ga]Ga-CHDT-PSMA-1/2/3 toward LNCaP cells

---

Extracellular saturation binding ("binding") of **[^{68}Ga]Ga-CHDT-PSMA-1/2/3** toward intact LNCaP cells as monolayer (**A**/**B**/**C1**) and spheroids (**C3**) as well as intracellular saturation binding ("internalization") toward intact LNCaP cells as monolayer (**C2**) and spheroids (**C4**). Data for total (grey triangles), nonspecific (open triangles, in the presence of 800 µM KuE), and specific binding (**[^{68}Ga]Ga-CHDT-PSMA-1** in red, **[^{68}Ga]Ga-CHDT-PSMA-2** in green, and **[^{68}Ga]Ga-CHDT-PSMA-3** in blue triangles) are shown as mean values (± SD) of one representative experiment, which was performed in duplicate or triplicate. The corresponding data are shown in the tables below. Data for missing graphs were not recorded.

[^{68}Ga]Ga-CHDT-PSMA-1

A Binding LNCaP Monolayer

Conc (nM)	Total bg (pmol/mg)		Nonsp bg (pmol/mg)		Spec bg (pmol/mg)	
	mean	SD	mean	SD	T-Nsp	SD
**0.45**	0.011	0.004	0.004	0.001	0.007	0.003
**0.85**	0.025	0.002	0.004	0.000	0.021	0.001
**1.74**	0.044	0.001	0.005	0.000	0.039	0.001
**3.32**	0.078	0.002	0.010	0.001	0.068	0.002
**6.65**	0.141	0.006	0.019	0.002	0.122	0.005
**13.09**	0.248	0.004	0.041	0.002	0.207	0.004
**25.89**	0.376	0.009	0.073	0.000	0.303	0.007
**50.00**	0.556	0.003	0.137	0.003	0.419	0.002

[^{68}Ga]Ga-CHDT-PSMA-2

B Internalization LNCaP Monolayer

Conc (nM)	Total int (pmol/mg)		Nonsp int (pmol/mg)		Spec int (pmol/mg)	
	mean	SD	mean	SD	T-Nsp	SD
**0.29**	0.004	0.001	0.000	0.000	0.004	0.001
**0.55**	0.009	0.001	0.003	0.001	0.005	0.001
**1.06**	0.019	0.002	0.004	0.000	0.016	0.002
**2.04**	0.031	0.002	0.008	0.001	0.024	0.002
**4.02**	0.069	0.003	0.016	0.001	0.052	0.003
**7.90**	0.123	0.002	0.032	0.000	0.091	0.002
**15.23**	0.227	0.006	0.061	0.002	0.165	0.007
**30.00**	0.362	0.006	0.126	0.000	0.236	0.006

[^{68}Ga]Ga-CHDT-PSMA-3

C1 Binding LNCaP Monolayer

Conc (nM)	Total bg (pmol/mg)		Nonsp bg (pmol/mg)		Spec bg (pmol/mg)	
	mean	SD	mean	SD	T-Nsp	SD
**1.00**	0.137	0.017	0.024	0.000	0.113	0.017
**1.91**	0.188	0.020	0.021	0.004	0.167	0.021
**3.68**	0.279	0.032	0.019	0.009	0.261	0.033
**7.07**	0.455	0.066	0.032	0.003	0.422	0.066
**13.78**	0.683	0.126	0.048	0.017	0.635	0.127
**26.51**	0.855	0.177	0.132	0.038	0.724	0.181
**51.7**	0.894	0.218	0.180	0.027	0.714	0.220
**100.0**	1.062	0.145	0.395	0.051	0.667	0.154

C2 Internalization LNCaP Monolayer

Conc (nM)	Total int (pmol/mg)		Nonsp int (pmol/mg)		Spec int (pmol/mg)	
	mean	SD	mean	SD	T-Nsp	SD
**1.00**	0.087	0.018	0.025	0.010	0.062	0.021
**1.91**	0.134	0.026	0.027	0.006	0.107	0.027
**3.68**	0.211	0.013	0.022	0.005	0.189	0.014
**7.07**	0.358	0.068	0.032	0.007	0.327	0.068
**13.78**	0.511	0.050	0.033	0.011	0.478	0.051
**26.51**	0.756	0.247	0.098	0.057	0.658	0.254
**51.7**	0.839	0.217	0.129	0.007	0.710	0.217
**100.0**	0.825	0.185	0.231	0.027	0.594	0.187

C3 Binding LNCaP Spheroids

Conc (nM)	Total bg (pmol/mg)		Nonsp bg (pmol/mg)		Spec bg (pmol/mg)	
	mean	SD	mean	SD	T-Nsp	SD
**0.92**	0.007	0.001	0.002	0.001	0.004	0.002
**1.80**	0.016	0.001	0.008	0.003	0.008	0.003
**3.49**	0.026	0.005	0.013	0.000	0.013	0.005
**6.79**	0.063	0.014	0.052	0.036	0.010	0.039
**13.64**	0.128	0.011	0.044	0.024	0.084	0.026
**26.44**	0.252	0.038	0.096	0.009	0.156	0.039
**51.0**	0.359	0.057	0.140	0.047	0.218	0.074
**100.0**	0.451	0.014	0.213	0.037	0.238	0.040

C4 Internalization LNCaP Spheroids

Conc (nM)	Total int (pmol/mg)		Nonsp int (pmol/mg)		Spec int (pmol/mg)	
	mean	SD	mean	SD	T-Nsp	SD
**0.92**	0.011	0.006	0.002	0.002	0.009	0.006
**1.80**	0.016	0.005	0.016	0.011	0.000	0.012
**3.49**	0.027	0.005	0.023	0.003	0.004	0.006
**6.79**	0.059	0.006	0.046	0.010	0.013	0.012
**13.64**	0.214	0.058	0.107	0.057	0.106	0.081
**26.44**	0.287	0.035	0.004	0.002	0.283	0.035
**51.0**	0.464	0.035	0.017	0.018	0.447	0.040
**100.0**	0.479	0.028	0.079	0.011	0.400	0.031

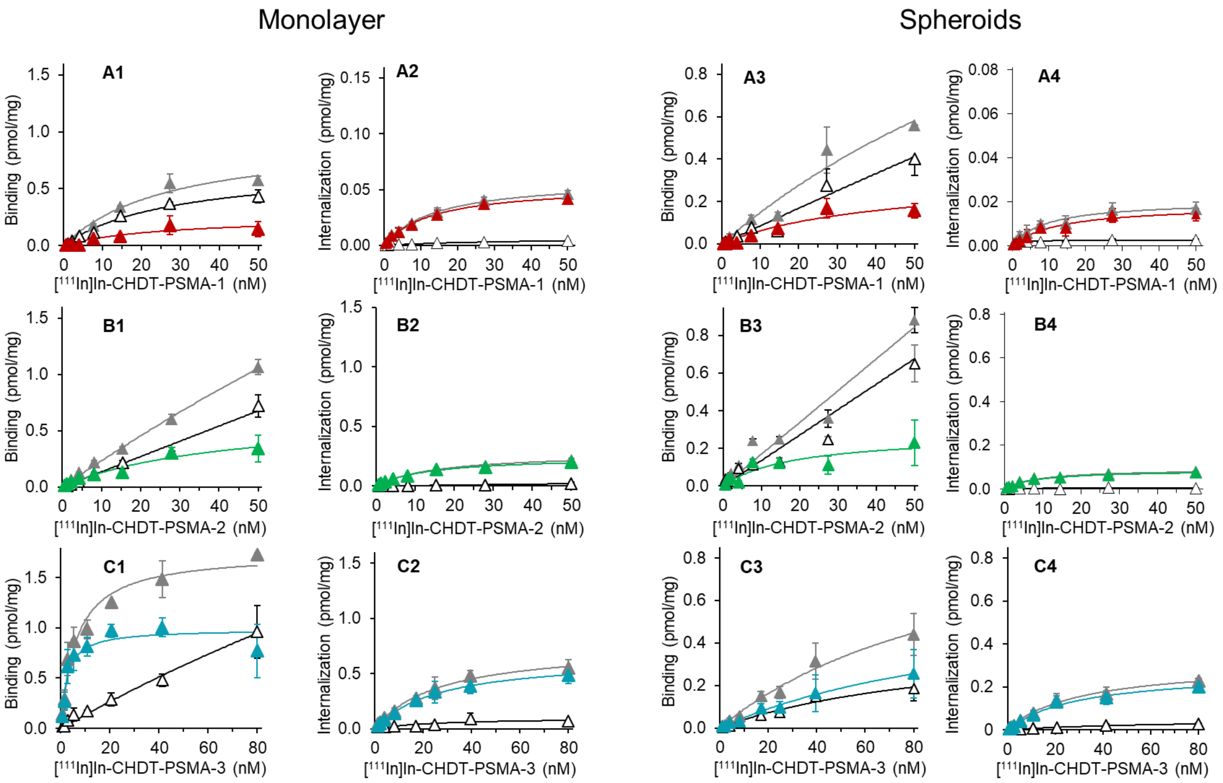

Figure S9: Exemplary saturation binding curves of [^{111}In]In-CHDT-PSMA-1/2/3 toward LNCaP cells

---

Extracellular saturation binding ("binding") of **[^{111}In]In-CHDT-PSMA-1/2/3** toward intact LNCaP cells as monolayer (**A1**/**B1**/**C1**) and spheroids (**A3/B3/C3**) as well as intracellular saturation binding ("internalization") toward intact LNCaP cells as monolayer (**A2/B2/C2**) and spheroids (**A4/B4/C4**). Data for total (grey triangles), nonspecific (open triangles, in the presence of 800 µM KuE) and specific binding (**[^{111}In]In-CHDT-PSMA-1** in red, **[^{111}In]In-CHDT-PSMA-2** in green, and **[^{111}In]In-CHDT-PSMA-3** in blue triangles) are shown as mean values (± SD) of one experiment, which was performed in duplicate or triplicate. The corresponding data are shown in the tables below.

[^{111}In]In-CHDT-PSMA-1

A1 Binding LNCaP Monolayer							A2 Internalization LNCaP Monolayer						
Conc (nM)	Total bg (pmol/mg)		Nonsp bg (pmol/mg)		Spec bg (pmol/mg)		Conc (nM)	Total int (pmol/mg)		Nonsp int (pmol/mg)		Spec int (pmol/mg)	
	mean	SD	mean	SD	T-Nsp	SD		mean	SD	mean	SD	T-Nsp	SD
**0.57**	0.011	0.002	0.009	0.000	0.002	0.002	**0.57**	0.002	0.0004	0.0002	0.0002	0.002	0.0005
**1.09**	0.022	0.002	0.016	0.003	0.006	0.003	**1.09**	0.004	0.0007	0.0002	0.0002	0.004	0.0007
**2.07**	0.051	0.007	0.040	0.003	0.011	0.008	**2.07**	0.009	0.0017	0.0008	0.0004	0.009	0.0018
**3.97**	0.087	0.008	0.082	0.002	0.005	0.008	**3.97**	0.013	0.0036	0.0007	0.0001	0.012	0.0036
**7.67**	0.172	0.018	0.114	0.011	0.059	0.021	**7.67**	0.020	0.0014	0.0010	0.0004	0.019	0.0015
**14.53**	0.342	0.011	0.260	0.009	0.081	0.014	**14.53**	0.030	0.0037	0.0021	0.0000	0.028	0.0037
**27.26**	0.548	0.083	0.370	0.013	0.178	0.084	**27.26**	0.040	0.0030	0.0028	0.0005	0.037	0.0030
**50.00**	0.574	0.036	0.431	0.059	0.143	0.069	**50.00**	0.046	0.0029	0.0041	0.0004	0.042	0.0029

A3 Binding LNCaP Speroids							A4 Internalization LNCaP Spheroids						
Conc (nM)	Total bg (pmol/mg)		Nonsp bg (pmol/mg)		Spec bg (pmol/mg)		Conc (nM)	Total int (pmol/mg)		Nonsp int (pmol/mg)		Spec int (pmol/mg)	
	mean	SD	mean	SD	T-Nsp	SD		mean	SD	mean	SD	T-Nsp	SD
**0.57**	0.006	0.001	0.004	0.000	0.002	0.001	**0.57**	0.0013	0.0004	0.0006	0.0002	0.001	0.0005
**1.09**	0.010	0.001	0.009	0.000	0.001	0.001	**1.09**	0.0019	0.0003	0.0010	0.0002	0.001	0.0004
**2.07**	0.032	0.014	0.012	0.003	0.020	0.006	**2.07**	0.0047	0.0005	0.0023	0.0012	0.002	0.0013
**3.97**	0.040	0.004	0.036	0.003	0.003	0.002	**3.97**	0.0059	0.0007	0.0020	0.0008	0.004	0.0010
**7.67**	0.116	0.019	0.078	0.001	0.038	0.008	**7.67**	0.0098	0.0040	0.0017	0.0006	0.008	0.0040
**14.53**	0.134	0.014	0.062	0.013	0.072	0.008	**14.53**	0.0101	0.0028	0.0018	0.0007	0.008	0.0029
**27.26**	0.443	0.108	0.274	0.012	0.169	0.047	**27.26**	0.0166	0.0011	0.0028	0.0000	0.014	0.0011
**50.00**	0.560	0.001	0.403	0.079	0.157	0.034	**50.00**	0.0169	0.0038	0.0028	0.0006	0.014	0.0039

[^{111}In]In-CHDT-PSMA-2

B1 Binding LNCaP Monolayer							B2 Internalization LNCaP Monolayer						
Conc (nM)	Total bg (pmol/mg)		Nonsp bg (pmol/mg)		Spec bg (pmol/mg)		Conc (nM)	Total int (pmol/mg)		Nonsp int (pmol/mg)		Spec int (pmol/mg)	
	mean	SD	mean	SD	T-Nsp	SD		mean	SD	mean	SD	T-Nsp	SD
**0.60**	0.018	0.002	0.007	0.000	0.011	0.002	**0.60**	0.012	0.002	0.000	0.000	0.011	0.002
**1.16**	0.032	0.005	0.009	0.000	0.023	0.005	**1.16**	0.022	0.002	0.001	0.000	0.021	0.002
**2.21**	0.063	0.002	0.021	0.001	0.042	0.003	**2.21**	0.039	0.003	0.002	0.000	0.037	0.003
**4.23**	0.126	0.009	0.049	0.006	0.078	0.011	**4.23**	0.060	0.005	0.002	0.000	0.058	0.005
**8.07**	0.221	0.012	0.111	0.014	0.111	0.019	**8.07**	0.088	0.005	0.003	0.001	0.085	0.005
**15.36**	0.342	0.013	0.213	0.021	0.129	0.025	**15.36**	0.146	0.008	0.006	0.001	0.140	0.008
**27.9**	0.602	0.047	0.300	0.016	0.302	0.049	**27.9**	0.167	0.012	0.009	0.001	0.158	0.012
**50.0**	1.064	0.067	0.720	0.099	0.343	0.119	**50.0**	0.218	0.016	0.021	0.011	0.197	0.019

B3 Binding LNCaP Spheroids

Conc (nM)	Total bg (pmol/mg)		Nonsp bg (pmol/mg)		Spec bg (pmol/mg)	
	mean	SD	mean	SD	T-Nsp	SD
**0.57**	0.040	0.013	0.035	0.111	0.005	0.056
**1.11**	0.044	0.008	0.011	0.001	0.033	0.004
**2.13**	0.067	0.007	0.025	0.087	0.042	0.044
**4.08**	0.111	0.007	0.094	0.207	0.017	0.104
**7.78**	0.239	0.021	0.113	0.004	0.126	0.011
**14.73**	0.249	0.051	0.128	0.046	0.120	0.034
**27.41**	0.358	0.022	0.248	0.019	0.111	0.015
**50.01**	0.880	0.095	0.651	0.064	0.229	0.057

B4 Internalization LNCaP Spheroids

Conc (nM)	Total int (pmol/mg)		Nonsp int (pmol/mg)		Spec int (pmol/mg)	
	mean	SD	mean	SD	T-Nsp	SD
**0.57**	0.0055	0.0005	0.0005	0.0000	0.005	0.001
**1.11**	0.0089	0.0004	0.0003	0.0003	0.009	0.001
**2.13**	0.0145	0.0040	0.0021	0.0010	0.012	0.004
**4.08**	0.0313	0.0028	0.0031	0.0000	0.028	0.003
**7.78**	0.0478	0.0046	0.0023	0.0004	0.046	0.005
**14.73**	0.0540	0.0142	0.0007	0.0001	0.053	0.014
**27.41**	0.0676	0.0146	0.0056	0.0037	0.062	0.015
**50.01**	0.0794	0.0197	0.0043	0.0004	0.075	0.020

[^{111}In]In-CHDT-PSMA-3

C1 Binding LNCaP Monolayer

Conc (nM)	Total bg (pmol/mg)		Nonsp bg (pmol/mg)		Spec bg (pmol/mg)	
	mean	SD	mean	SD	T-Nsp	SD
**0.73**	0.138	0.020	0.019	0.003	0.119	0.020
**1.44**	0.290	0.093	0.020	0.003	0.269	0.093
**2.78**	0.689	0.167	0.077	0.017	0.612	0.168
**5.34**	0.867	0.141	0.136	0.067	0.731	0.156
**10.76**	0.987	0.093	0.171	0.010	0.815	0.093
**20.56**	1.256	0.004	0.285	0.064	0.971	0.064
**41.4**	1.485	0.182	0.480	0.060	1.005	0.096
**80.0**	1.730	0.019	0.960	0.264	0.769	0.265

C2 Internalization LNCaP Monolayer

Conc (nM)	Total int (pmol/mg)		Nonsp int (pmol/mg)		Spec int (pmol/mg)	
	mean	SD	mean	SD	T-Nsp	SD
**1.17**	0.044	0.004	0.017	0.000	0.027	0.004
**2.14**	0.061	0.004	0.018	0.000	0.043	0.004
**4.01**	0.100	0.007	0.018	0.000	0.082	0.007
**8.03**	0.159	0.029	0.021	0.001	0.138	0.029
**16.97**	0.277	0.018	0.025	0.002	0.253	0.018
**24.75**	0.367	0.099	0.034	0.004	0.333	0.099
**39.5**	0.476	0.050	0.090	0.049	0.386	0.070
**80.0**	0.550	0.075	0.066	0.001	0.484	0.075

C3 Binding LNCaP Spheroids

Conc (nM)	Total bg (pmol/mg)		Nonsp bg (pmol/mg)		Spec bg (pmol/mg)	
	mean	SD	mean	SD	T-Nsp	SD
**1.17**	0.010	0.005	0.003	0.000	0.007	0.005
**2.14**	0.014	0.003	0.007	0.001	0.007	0.003
**4.01**	0.033	0.012	0.010	0.000	0.023	0.012
**8.03**	0.054	0.006	0.024	0.002	0.030	0.006
**16.97**	0.150	0.021	0.058	0.008	0.092	0.023
**24.75**	0.167	0.029	0.072	0.009	0.094	0.031
**39.5**	0.313	0.085	0.149	0.018	0.164	0.087
**80.0**	0.439	0.098	0.185	0.056	0.254	0.113

C4 Internalization LNCaP Spheroids

Conc (nM)	Total int (pmol/mg)		Nonsp int (pmol/mg)		Spec int (pmol/mg)	
	mean	SD	mean	SD	T-Nsp	SD
**0.73**	0.010	0.002	0.003	0.001	0.007	0.002
**1.44**	0.013	0.001	0.005	0.002	0.008	0.002
**2.78**	0.020	0.001	0.004	0.000	0.016	0.001
**5.34**	0.043	0.003	0.005	0.000	0.038	0.003
**10.76**	0.076	0.016	0.008	0.001	0.068	0.017
**20.56**	0.138	0.029	0.009	0.001	0.129	0.029
**41.4**	0.168	0.031	0.019	0.008	0.148	0.032
**80.0**	0.228	0.013	0.028	0.004	0.200	0.013

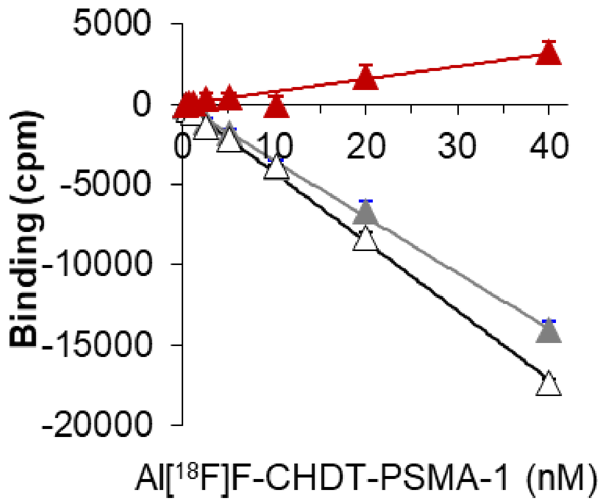

Figure S10: Exemplary saturation binding curves of Al[^{18}F]F-CHDT-PSMA-1 toward PC3 cells

Saturation binding of **Al[^{18}F]F-CHDT-PSMA-1** toward PC3 cell homogenate. Data for total (grey triangles), nonspecific (open triangles, in the presence of 800 µM KuE) and specific binding (red) are shown as mean values (± SD) of one experiment, which was performed in triplicate. The corresponding data are shown in the table below.

Binding PC3 homogenate						
Conc (nM)	Total bg (cpm)		Nonsp bg (cpm)		Spec bg (cpm)	
	mean	SD	mean	SD	T-Nsp	SD
**0.33**	-165.1	111.8	-63.7	202.5	0	231.3
**0.62**	-217.8	151.1	-315.8	146.0	98	210.1
**1.24**	-477.6	117.7	-514.3	122.5	37	169.9
**2.54**	-1081.9	207.9	-1454.9	263.5	373	335.6
**5.03**	-1822.9	250.9	-2225.4	20.0	402	251.7
**10.15**	-3981.7	470.1	-3852.4	88.0	0	478.3
**19.96**	-6628.9	619.6	-8332.9	382.0	1704	727.9
**40**	-14035.8	566.1	-17320.6	187.5	3285	596.3

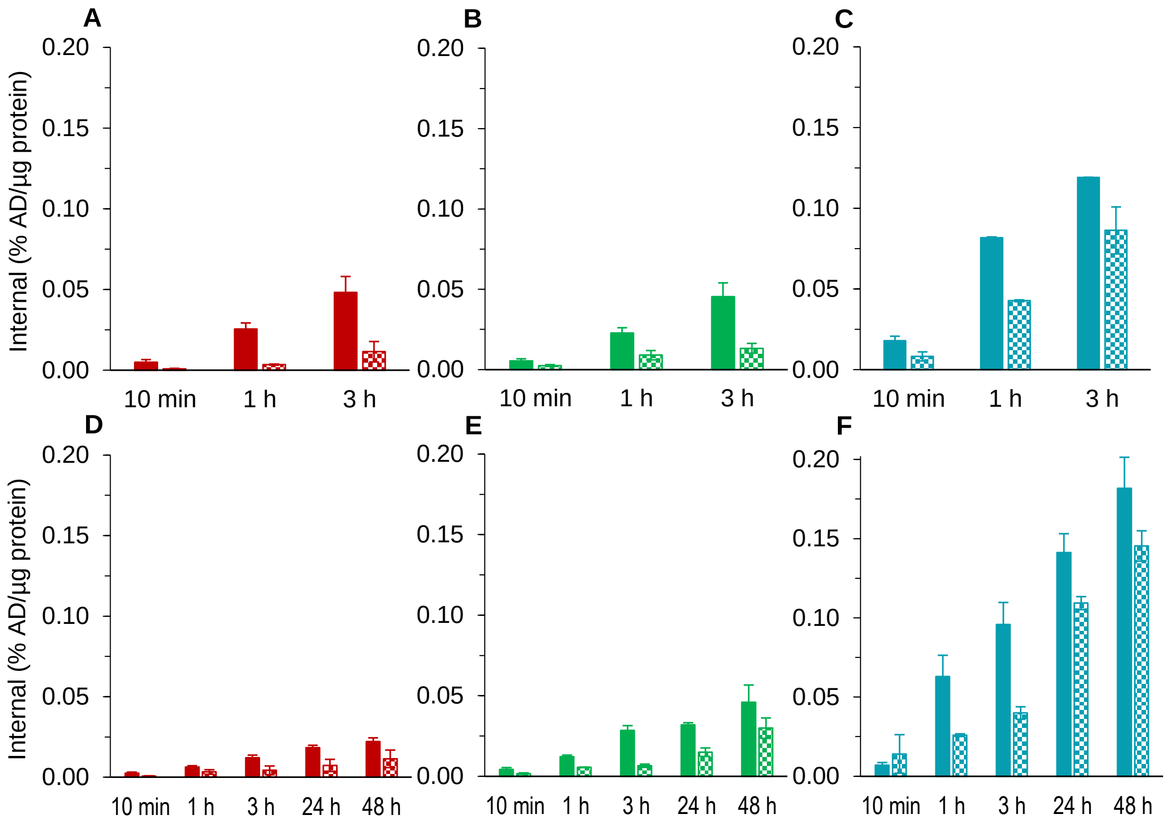

Figure S11: Internalization behavior of the ^{68}Ga- and ^{111}In-labeled CHDT-PSMA-1/2/3

Time-depending internalization using LNCaP as monolayer with specific internalization (filled bars) and specific internalization after treatment with MβCD (3 mM, checkered bars); **[^{68}Ga]Ga-CHDT-PSMA-1/2/3** (**A**/**B**/**C**) and **[^{111}In]In-CHDT-PSMA-1/2/3** (**D**/**E**/**F**). Data shown are mean values (± SEM) of 2-5 separate experiments, each performed in triplicate to quintuplicate with data for **^{68}Ga/^{111}In-labeled CHDT-PSMA-1** in red, for **^{68}Ga/^{111}In-labeled CHDT-PSMA-2** in green, and for **^{68}Ga/^{111}In-labeled CHDT-PSMA-3** in blue. The individual mean values for each experiment are shown in the tables below.

Time	[68Ga]Ga-CHDT-PSMA-1 (A)		[68Ga]Ga-CHDT-PSMA-2 (B)		[68Ga]Ga-CHDT-PSMA-3 (C)	
	specific	spec MβCD	specific	spec MβCD	specific	spec MβCD
10 min	0.003164	0.001007	0.002739	0.001959	0.015158	0.005569
	0.009682	0.001515	0.002191	0.001099	0.020711	0.011021
	0.00296652	0.000543	0.005328	0.001712		
	0.004268	0.000539	0.008964	0.003451		
			0.003838	0.00164		
			0.00955	0.005002		
1 h	0.02008935	0.003	0.011785	0.002092	0.082274	0.043388
	0.030906	0.00398	0.021258	0.00381	0.081498	0.042215
			0.019927	0.008216		
			0.036755	0.006141		
			0.031866	0.010201		
			0.021361	0.007478		
			0.015932	0.024918		
3 h	0.062945	0.030524	0.033563	0.000402	0.119265	0.100967
	0.062945	0.030524	0.033563	0.000402	0.119128	0.071722
	0.0517021	0.00539	0.052921	0.01181		
	0.058644	0.006459	0.073286	0.012175		
			0.068314	0.024287		
			0.052545	0.020798		
			0.011604	0.016092		

Time	[111In]In-CHDT-PSMA-1 (D)		[111In]In-CHDT-PSMA-2 (E)		[111In]In-CHDT-PSMA-3 (F)	
	specific	spec MβCD	specific	spec MβCD	specific	spec MβCD
10 min	0.00264	0.00045	0.006003	0.001919	0.004325	0.03834
	0.002361	0.000721	0.002443	0.000644	0.01009	0.000425
	0.001317	0.000441	0.006125	0.002784	0.006594	0.003527
	0.00411	0.001203	0.002629	0.001113		
1 h	0.007254	0.001177	0.013801	0.00539	0.067614	0.024969
	0.004494	0.002298	0.012395	0.005926	0.083552	0.027718
	0.007242	0.00637	0.010344	0.005387	0.037757	0.025076
3 h	0.011925	0.000229	0.036592	0.005201	0.092946	0.035718
	0.012006	0.002172	0.027519	0.005711	0.121048	0.036185
	0.008712	0.002499	0.027169	0.009622	0.073606	0.047844
	0.016022	0.012195	0.022597	0.005721		
24 h	0.020085	0.000692	0.035252	0.014051	0.139122	0.113738
	0.014081	0.000943	0.030782	0.012392	0.162762	0.101044
	0.01787	0.006964	0.02931	0.022489	0.12175	0.113129
	0.021213	0.020792	0.032592	0.010677		
48 h	0.015654	0.000514	0.043856	0.022035	0.200323	0.161254
	0.025831		0.076399	0.047425	0.202363	0.146879
	0.021493	0.016581	0.028237	0.01959	0.142656	0.128196
	0.025358	0.024175	0.035317	0.030483		

Table S1: Individual values for $logD_{7.4}$ and serum stability of the radiolabeled CHDT conjugated compounds

Summary data are shown in Table 1 in the main article.

	Radiolabel	Conjugates CHDT-PSMA-1	CHDT-PSMA-2	CHDT-PSMA-3
**log $D_{7.4}$**	**$Al^{18}F$**	-3.85	-3.51	-3.47
		-3.36	-3.19	-3.20
		-3.60	-3.32	-3.63
	**^{68}Ga**	-3.34	-3.20	-3.26
		-3.43	-2.99	-2.93
		-3.30	-3.14	-3.10
	**^{111}In**	-4.38	-3.59	-3.76
		-4.21	-3.35	-4.00
		-4.10	-3.39	-3.84
**Percentage of intact radioligand after 3 h in human serum (*) after 24 h, (**) after 48 h**	**$Al^{18}F$**	98.9	99.3	99.2
		99.0	98.7	99.2
		98.8	99.3	99.6
	**^{68}Ga**	98.8	97.7	98.6
		94.4	97.4	99.6
			97.0	99.3
	**^{111}In**	99.7	99.6	99.5
		99.3	98.3	98.8
		99.4	98.6	99.2
	*****	99.8	98.9	98.9
		99.5	98.8	99.6
		99.5	97.6	98.9
	******	99.7	99.9	99.9
		99.3	98.5	99.5
		98.9	97.6	98.7

Table S2: Individual data (percent binding [^{177}Lu]Lu-PSMA-617) for each competition binding experiment

Competition binding curves are shown in Figure 2 in the main article (mean ± SEM).

	**CHDT-PSMA-1**				
Conc (log M)	Percent binding of **[^{177}Lu]Lu-PSMA-617** using LNCaP cell homogenate				
-10.02	102.32	100.52	101.23	97.71	99.12
-9.42	-	-	-	-	100.88
-8.72	97.68	99.48	98.77	101.73	96.48
-8.12	94.72	94.67	93.47	100.56	87.23
-7.52	81.25	86.02	77.31	85.79	83.79
-6.91	55.71	62.48	56.16	69.28	71.29
-6.31	27.43	36.68	29.34	37.22	42.79
-5.71	8.22	12.93	10.09	14.78	20.63
-5.11	3.12	1.85	3.72	6.82	13.63
-4.51	1.83	7.30	2.06	1.43	5.81
-3.90	1.27	5.23	0.85	0.02	

**CHDT-PSMA-2**					**CHDT-PSMA-3**				
Percent binding of **[^{177}Lu]Lu-PSMA-617** using LNCaP cell homogenate									
Conc (log M)					Conc (log M)				
					**-10.61**	103.27	101.81	103.41	103.71
**-9.32**	104.83	98.72	102.28	101.96	**-10.01**				96.40
**-8.72**	95.17	101.28	97.72	98.04	**-9.41**	96.73	98.19	96.59	103.60
**-8.12**	87.04	92.43	85.50	84.31	**-8.81**	89.20	77.88	91.96	88.51
**-7.52**	71.11	76.49	68.16	66.52	**-8.21**	69.07	53.29	71.10	76.54
**-6.91**	43.14	51.07	46.32	36.15	**-7.60**	45.81	32.70	42.56	49.72
**-6.31**	21.42	25.27	27.51	16.78	**-7.00**	29.13	22.17	23.47	23.71
**-5.71**	7.84	9.08	15.23	4.49	**-6.40**	18.72	12.22	12.79	15.90
**-5.11**	2.87	3.39	10.22	2.33	**-5.80**	12.25	7.20	3.04	1.05
**-4.51**	1.27	0.20	9.25	2.62	**-5.20**	10.15	4.25	2.28	1.30
**-3.90**	0.11	0.32	8.88	0.18	**-4.59**	9.39	0.00	0.96	0.00

	PSMA-617							
	Percent binding of **[^{177}Lu]Lu-PSMA-617** using LNCaP cell homogenate							
Conc (log M)								
**-10.64**	102.78	101.09	106.12	100.86	101.43	97.77	102.88	100.91
**-10.04**				99.14	98.57	102.23	97.12	99.09
**-9.44**	97.22	98.91	93.88	95.25	88.03	100.72	94.10	99.42
**-8.83**	87.16	90.71	91.88	88.01	78.41	82.42	91.59	81.47
**-8.23**	49.95	40.47	60.60	64.46	56.46	58.30	58.93	52.75
**-7.63**	34.42	25.85	36.58	28.09	29.72	32.79	35.53	29.10
**-7.03**	8.61	13.96	14.16	10.38	11.38	21.44	19.59	12.57
**-6.43**	2.97	6.68	10.03	2.89	4.62	14.60	15.61	4.25
**-5.82**	2.15	2.86	3.65	2.20	0.64	12.99	10.97	1.21
**-5.22**	0.00	5.05	1.26	1.09	0.26	12.85	14.89	0.23
**-4.62**	0.00	3.16	0.00	0.16	0.49	97.77	102.88	100.91

KuE							PSMA-1007		
Percent binding of **[^{177}Lu]Lu-PSMA-617** using LNCaP cell homogenate									
Conc (log M)							Conc (log M)		
							**-11.00**	103.28	103.21
**-8.50**	101.45	99.39	100.63	100.46	99.27	101.70	**-10.33**		96.79
**-7.64**	98.55	100.61	99.37	99.54	100.73	98.30	**-9.73**	96.72	93.33
**-7.04**	94.18	104.24	99.93	102.02	96.91	111.07	**-9.13**	85.95	87.59
**-6.44**	93.16	95.64	94.09	96.03	89.87	98.41	**-8.53**	59.00	49.35
**-5.83**	88.84	92.06	90.75	92.03	96.71	94.43	**-7.93**	29.40	22.14
**-5.23**	77.08	90.00	70.64	77.45	90.80	76.96	**-7.32**	12.88	23.72
**-4.63**	55.04	66.96	37.53	36.89	70.30	52.77	**-6.72**	9.75	10.95
**-4.03**	33.58	31.86	14.12	20.31	36.57	27.22	**-6.12**	8.56	11.40
**-3.43**	10.99	15.46	4.54	12.21	18.29	10.51	**-5.52**	5.59	8.68
**-2.82**	7.02	8.93	1.24	4.16	4.70	7.68	**-4.59**	4.54	8.83

Table S3: Individual mean values for K_i determined from each competition binding experiment

Mean (± SEM) are shown in Table 2 in the main article.

	CHDT-PSMA-1	CHDT-PSMA-2	CHDT-PSMA-3	PSMA-617	PSMA-1007	KuE
	85.47	48.06	7.65	4.10	2.00	2,132
	119.5	71.23	3.21	2.59	1.39	16,070
	86.89	40.45	9.15	5.55		22,670
	152	34.45	12.6	5.49		7,893
	132			4.57		7,995
				3.33		11,300
				4.02		
				3.71		
**mean**	**115**	**49**	**8.1**	**4.2**	**1.7**	**11,300**
SEM	13	8	1.9	0.4	0.3	2,900

Table S4: Parameters for extracellular and intracellular saturation binding of [^{68}Ga]Ga-CHDT-PSMA-1/2/3

Data shown are mean values (± SEM) of 2-6 separate experiments. n.d. denotes not determined, M denotes monolayer, and S denotes spheroids. [a]Assay performed using cell homogenates. [b]Only one assay was performed (in triplicates).

		Binding		Internalization	
**Conjugate**	**LNCaP**	**K_d (nM)**	**B_{max} (pmol/mg)**	**$K_{d,int}$ (nM)**	**$B_{max,int}$ (pmol/mg)**
**CHDT-PSMA-1**	M<br>S	31.9 ± 5.4[a]<br>n.d.	0.40 ± 0.08[a]<br>n.d.	-	-
**CHDT-PSMA-2**	M<br>S	45.5 ± 8.6[a]<br>n.d.	0.64 ± 0.18[a]<br>n.d.	-	-
**CHDT-PSMA-3**	M<br>S	11.1 ± 5.7<br>26.7 ± 16.0[b]	0.85 ± 0.28<br>0.26 ± 0.27[b]	17.3 ± 9.7<br>46.2 ± 28.7[b]	1.16 ± 0.51<br>0.75 ± 0.20[b]

The individual mean values for each experiment are shown in the subsequent table.

		Binding		Internalization	
**Conjugate**	**LNCaP**	**K_d (nM)**	**B_{max} (pmol/mg)**	**$K_{d,int}$ (nM)**	**$B_{max,int}$ (pmol/mg)**
**CHDT-PSMA-1**	M[a]	29.5<br>18.2<br>28.4<br>21.6<br>39.7<br>54.0	0.65<br>0.53<br>0.29<br>0.21<br>0.16<br>0.53		
	S	n.d.	n.d.		
**CHDT-PSMA-2**	M[a]	74.8<br>34.3<br>31.3<br>55.1<br>31.9	0.45<br>0.51<br>1.35<br>0.56<br>0.35		
	S	n.d.	n.d.		
**CHDT-PSMA-3**	M	7.2<br>22.4<br>3.7	1.03<br>1.23<br>0.31	10.1<br>36.5<br>5.4	0.94<br>2.13<br>0.42
	S[b]	27.7	0.31	46.2	0.75

Table S5: Parameters for extracellular and intracellular saturation binding of [^{111}In]In-CHDT-PSMA-1/2/3

Data shown are mean values (± SEM) of 2-4 separate experiments. n.d. denotes not determined, M denotes monolayer, and S denotes spheroids.

		Binding		Internalization	
**Conjugate**	**LNCaP**	**K_d (nM)**	**B_{max} (pmol/mg)**	**$K_{d,int}$ (nM)**	**$B_{max,int}$ (pmol/mg)**
**CHDT-PSMA-1**	M	29.7 ± 5.9	0.32 ± 0.12	16.9 ± 3.6	0.08 ± 0.03
	S	38.7 ± 3.0	0.22 ± 0.12	14.5 ± 1.5	0.04 ± 0.02
**CHDT-PSMA-2**	M	25.3 ± 7.8	0.45 ± 0.11	11.4 ± 2.4	0.21 ± 0.04
	S	40.3 ± 21.5	0.20 ± 0.05	38.2 ± 29.5	0.15 ± 0.05
**CHDT-PSMA-3**	M	20.0 ± 2.8	0.70 ± 0.21	17.0 ± 9.6	0.57 ± 0.08
	S	62.0 ± 11.0	0.30 ± 0.10	25.9 ± 3.5	0.32 ± 0.01

The individual mean values for each experiment are shown in the subsequent table.

		Binding		Internalization	
**Conjugate**	**LNCaP**	**K_d (nM)**	**B_{max} (pmol/mg)**	**$K_{d,int}$ (nM)**	**$B_{max,int}$ (pmol/mg)**
**CHDT-PSMA-1**	M	30.8	0.66	13.3	0.05
		17.1	0.25	20.5	0.11
		25.9	0.25		
		45.2	0.12		
	S	41.7	0.33	13.0	0.03
		35.6	0.10	16.1	0.06
**CHDT-PSMA-2**	M	19.5	0.38	13.7	0.25
		40.7	0.66	9.0	0.17
		15.8	0.30		
	S	61.7	0.15	67.7	0.20
		18.8	0.25	8.8	0.10
**CHDT-PSMA-3**	M	22.8	0.50	26.7	0.65
		17.2	0.91	7.4	0.49
	S	72.9	0.21	22.4	0.31
		51.0	0.40	29.4	0.33

Table S6: Data for the exemplary saturation binding curves of Al[^{18}F]F-CHDT-PSMA-1/2/3

The respective saturation binding curves are shown in Figure 3 in the main article.

Al[^{18}F]F-CHDT-PSMA-1

A1 Binding LNCaP Monolayer							A2 Internalization LNCaP Monolayer						
Conc (nM)	Total bg (pmol/mg)		Nonsp bg (pmol/mg)		Spec bg (pmol/mg)		Conc (nM)	Total int (pmol/mg)		Nonsp int (pmol/mg)		Spec int (pmol/mg)	
	mean	SD	mean	SD	T-Nsp	SD		mean	SD	mean	SD	T-Nsp	SD
**0.90**	0.134	0.020	0.036	0.012	0.098	0.023	**0.90**	0.093	0.008	0.013	0.004	0.080	0.009
**1.74**	0.176	0.016	0.062	0.006	0.114	0.017	**1.74**	0.168	0.053	0.030	0.010	0.138	0.054
**3.32**	0.429	0.018	0.104	0.004	0.325	0.018	**3.32**	0.303	0.020	0.060	0.027	0.243	0.034
**6.61**	0.808	0.033	0.140	0.015	0.668	0.036	**6.61**	0.597	0.033	0.068	0.010	0.529	0.035
**13.29**	1.562	0.185	0.307	0.007	1.255	0.185	**13.29**	0.834	0.115	0.047	0.006	0.787	0.115
**24.95**	2.098	0.534	0.562	0.013	1.536	0.534	**24.95**	0.982	0.247	0.058	0.002	0.924	0.247
**50.7**	3.024	0.521	1.055	0.224	1.969	0.567	**50.7**	1.776	0.134	0.112	0.034	1.664	0.138
**100.0**	4.375	0.384	2.367	0.234	2.008	0.450	**100.0**	1.868	0.233	0.149	0.033	1.719	0.235

A3 Binding LNCaP Spheroids							A4 Internalization LNCaP Spheroids						
Conc (nM)	Total bg (pmol/mg)		Nonsp bg (pmol/mg)		Spec bg (pmol/mg)		Conc (nM)	Total int (pmol/mg)		Nonsp int (pmol/mg)		Spec int (pmol/mg)	
	mean	SD	mean	SD	T-Nsp	SD		mean	SD	mean	SD	T-Nsp	SD
**0.90**	0.024	0.013	0.018	0.012	0.007	0.018	**0.90**	0.018	0.018	0.018	0.018	0.018	0.003
**1.74**	0.031	0.004	0.010	0.003	0.021	0.005	**1.74**	0.005	0.005	0.005	0.005	0.005	0.015
**3.32**	0.078	0.046	0.033	0.018	0.045	0.049	**3.32**	0.049	0.049	0.049	0.049	0.049	0.025
**6.61**	0.245	0.106	0.152	0.048	0.094	0.117	**6.61**	0.117	0.117	0.117	0.117	0.117	0.015
**13.29**	0.463	0.100	0.232	0.078	0.231	0.126	**13.29**	0.126	0.126	0.126	0.126	0.126	0.045
**24.95**	0.389	0.026	0.160	0.032	0.229	0.041	**24.95**	0.041	0.041	0.041	0.041	0.041	0.024
**50.7**	0.734	0.059	0.250	0.092	0.484	0.110	**50.7**	0.110	0.110	0.110	0.110	0.110	0.054
**100.0**	1.467	0.279	0.787	0.270	0.680	0.388	**100.0**	0.388	0.388	0.388	0.388	0.388	0.099

Al[^{18}F]F-CHDT-PSMA-2

B1 Binding LNCaP Monolayer							B2 Internalization LNCaP Monolayer						
Conc (nM)	Total bg (pmol/mg)		Nonsp bg (pmol/mg)		Spec bg (pmol/mg)		Conc (nM)	Total int (pmol/mg)		Nonsp int (pmol/mg)		Spec int (pmol/mg)	
	mean	SD	mean	SD	T-Nsp	SD		mean	SD	mean	SD	T-Nsp	SD
**0.49**	0.070	0.011	0.036	0.000	0.034	0.011	**0.55**	0.074	0.025	0.042	0.000	0.032	0.025
**0.97**	0.143	0.026	0.053	0.010	0.089	0.028	**1.10**	0.137	0.004	0.007	0.000	0.130	0.004
**1.86**	0.243	0.047	0.077	0.034	0.166	0.058	**2.15**	0.168	0.018	0.027	0.000	0.141	0.018
**3.60**	0.459	0.020	0.120	0.338	0.338	0.339	**4.18**	0.331	0.005	0.016	0.000	0.315	0.005
**7.44**	0.738	0.084	0.289	0.040	0.449	0.092	**8.08**	0.492	0.022	0.012	0.000	0.480	0.022
**13.60**	1.484	0.220	0.538	0.106	0.946	0.244	**15.90**	0.672	0.027	0.026	0.000	0.645	0.027
**26.5**	2.097	0.210	1.070	0.100	1.027	0.233	**30.9**	0.730	0.221	0.031	0.000	0.698	0.221
**50.0**	3.187	0.415	2.019	0.221	1.168	0.470	**60.0**	0.910	0.023	0.048	0.000	0.862	0.023

B3 Binding LNCaP Spheroids

Conc (nM)	Total bg (pmol/mg)		Nonsp bg (pmol/mg)		Spec bg (pmol/mg)	
	mean	SD	mean	SD	T-Nsp	SD
**0.49**	0.048	0.015	0.026	0.008	0.022	0.017
**0.97**	0.060	0.007	0.037	0.012	0.023	0.014
**1.86**	0.195	0.042	0.034	0.011	0.161	0.043
**3.60**	0.282	0.232	0.069	0.000	0.213	0.232
**7.44**	0.458	0.127	0.157	0.023	0.301	0.129
**13.60**	0.569	0.103	0.311	0.138	0.258	0.172
**26.5**	1.020	0.130	0.515	0.054	0.505	0.141
**50.0**	1.605	0.248	0.907	0.098	0.699	0.267

B4 Internalization LNCaP Spheroids

Conc (nM)	Total int (pmol/mg)		Nonsp int (pmol/mg)		Spec int (pmol/mg)	
	mean	SD	mean	SD	T-Nsp	SD
**0.49**	0.009	0.001	0.004	0.001	0.004	0.002
**0.97**	0.014	0.003	0.004	0.002	0.010	0.003
**1.86**	0.029	0.006	0.006	0.001	0.022	0.006
**3.60**	0.042	0.014	0.010	0.002	0.032	0.014
**7.44**	0.115	0.058	0.027	0.020	0.088	0.061
**13.60**	0.198	0.060	0.010	0.001	0.188	0.060
**26.5**	0.314	0.169	0.019	0.008	0.294	0.169
**50.0**	0.223	0.007	0.019	0.000	0.204	0.007

Al[^{18}F]F-CHDT-PSMA-3

C1 Binding LNCaP Monolayer

Conc (nM)	Total bg (pmol/mg)		Nonsp bg (pmol/mg)		Spec bg (pmol/mg)	
	mean	SD	mean	SD	T-Nsp	SD
**1.02**	0.257	0.028	0.064	0.012	0.193	0.030
**1.98**	0.472	0.037	0.099	0.002	0.373	0.037
**3.80**	0.755	0.112	0.168	0.019	0.587	0.113
**7.27**	0.843	0.214	0.287	0.086	0.556	0.231
**13.97**	1.980	0.484	0.520	0.033	1.460	0.485
**24.92**	2.756	1.038	0.755	0.029	2.001	1.039
**52.1**	3.106	0.461	1.587	0.134	1.519	0.480
**100.0**	5.627	0.078	3.503	0.891	2.124	0.894

C2 Internalization LNCaP Monolayer

Conc (nM)	Total int (pmol/mg)		Nonsp int (pmol/mg)		Spec int (pmol/mg)	
	mean	SD	mean	SD	T-Nsp	SD
**0.98**	0.207	0.024	0.044	0.021	0.163	0.032
**1.91**	0.220	0.058	0.117	0.007	0.103	0.059
**3.69**	0.501	0.129	0.047	0.004	0.454	0.129
**7.18**	0.713	0.203	0.106	0.007	0.607	0.203
**14.05**	1.180	0.213	0.106	0.019	1.074	0.214
**26.89**	1.601	0.157	0.179	0.061	1.422	0.168
**53.2**	1.687	0.359	0.235	0.002	1.451	0.359
**100.0**	1.650	0.282	0.147	0.014	1.502	0.282

C3 Binding LNCaP Spheroids

Conc (nM)	Total bg (pmol/mg)		Nonsp bg (pmol/mg)		Spec bg (pmol/mg)	
	mean	SD	mean	SD	T-Nsp	SD
**2.98**	0.157	0.027	0.070	0.000	0.087	0.027
**5.97**	0.174	0.015	0.095	0.000	0.078	0.015
**11.44**	0.422	0.069	0.148	0.000	0.274	0.069
**22.70**	0.617	0.131	0.184	0.000	0.433	0.131
**42.1**	0.861	0.092	0.236	0.000	0.624	0.092
**80.0**	1.441	0.233	0.965	0.000	0.476	0.233

C4 Internalization LNCaP Spheroids

Conc (nM)	Total int (pmol/mg)		Nonsp int (pmol/mg)		Spec int (pmol/mg)	
	mean	SD	mean	SD	T-Nsp	SD
**1.02**	0.031	0.011	0.009	0.001	0.022	0.011
**1.98**	0.054	0.006	0.010	0.001	0.044	0.006
**3.80**	0.091	0.018	0.024	0.018	0.067	0.026
**7.27**	0.159	0.011	0.022	0.007	0.137	0.013
**13.97**	0.276	0.048	0.021	0.000	0.254	0.048
**24.92**	0.295	0.010	0.026	0.003	0.269	0.011
**52.1**	0.568	0.180	0.068	0.024	0.500	0.182
**100.0**	0.544	0.017	0.068	0.003	0.476	0.018

Table S7: Individual mean values for K_d and B_{max} determined from each saturation binding experiment with Al[18F]F-CHDT-PSMA-1/2/3 and [18F]PSMA-1007

The respective mean (± SEM) values are shown in Table 3 in the main article.

		Binding		Internalization	
**Conjugate**	**LNCaP**	**K_d (nM)**	**B_{max} (pmol/mg)**	**$K_{d,int}$ (nM)**	**$B_{max,int}$ (pmol/mg)**
**CHDT-PSMA-1**	M	37.5	1.30	11.0	0.33
		15.4	2.44	25.7	2.24
	S	15.1	1.84	26.7	0.26
		68.0	1.13	48.1	0.71
**CHDT-PSMA-2**	M	6.2	1.16	8.9	0.97
		20.0	1.3	24.0	0.92
		12.6	1.52	10.2	0.47
	S	11.4	1.20	16.7	0.14
		20.3	0.93	14.6	0.51
**CHDT-PSMA-3**	M	44.0	1.64	35.8	1.98
		6.6	2.29	6.0	0.71
		11.0	2.61	4.8	0.89
		17.0		10.9	1.77
	S	18.2	0.17	57.3	1.75
		89.8	0.46	0.4	0.14
		7.7	0.15	25.0	0.64
			2.26	63.0	0.89
**PSMA-1007**	M	9.0	1.17	16.1	1.23
		1.9	1.05	4.2	0.81
	S	11.7	1.43	7.2	0.99
		14.2	0.29	44.4	0.35

M = monolayer; S = spheroids

Table S8: Individual mean values for the internalization determined from each internalization experiment with Al[^{18}F]F-CHDT-PSMA-1/2/3 and [^{18}F]PSMA-1007

The respective bar graphs of mean (± SEM) are shown in Figure 4 in the main article. The values are given in % AD/µg protein.

Time	Al[^{18}F]F-CHDT-PSMA-1		Al[^{18}F]F-CHDT-PSMA-2		Al[^{18}F]F-CHDT-PSMA-3		[^{18}F]PSMA-1007	
	specific	spec MβCD	specific	spec MβCD	specific	spec MβCD	specific	spec MβCD
10 min	0.005338	0.002220837	0.002731	0.00146	0.007117571	0.002781	0.036962	0.022316
	0.002753		0.002731	0.00146	0.0112609	0.00902885	0.036962	0.022316
	0.0014186	0.000953004	0.0066586	0.000841	0.00907706	0.00636696	0.026161	0.008008
	0.004874	0.00036252						
	0.004758	0.000635578						
	0.010646	0.0052607						
	0.0014324	0.001926832						
1 h	0.045553	0.010476199	0.052336	0.010207	0.063910383	0.026437	0.07765	0.028328
	0.050225	0.0239692	0.022931	0.005974	0.0897806	0.038899405	0.10709	0.103804
	0.1084241	0.037806045	0.04930909	0.02164	0.08852552	0.03400334	0.117084	0.109069
3 h	0.07009	0.016845567	0.14384	0.038853	0.07948292	0.020095	0.229519	0.045655
	0.051929		0.077747	0.010807	0.1989737	0.045167606	0.170097	0.147839
	0.0383653	0.0015014	0.08637015	0.033414	0.17108623	0.07366584	0.156221	0.149495
	0.113953	0.02029044						
	0.095522	0.0138721						
	0.118793	0.0610216						
	0.1343468	0.049404948						

Table S9: Ex vivo biodistribution data for Al[^{18}F]F-CHDT-Pe in SKH1 mice

For ex vivo biodistribution studies, two groups of SKH1 mice (n=4, body weight of 28.1 ± 1.9 g) received 0.20 MBq of **Al[^{18}F]F-CHDT-Pe** and were sacrificed under desflurane anesthesia at 60 and 240 min p.i. Organs and tissues of interest were removed, weighed, and the activity was measured in a cross-calibrated well counter and dose calibrator. The decay corrected data were normalized to the amount of injected activity calculated from the activity of injection syringes before and after injection and expressed as percentage of injected activity per gram tissue (%ID/g tissue or %ID for intestine and urine). Values are quoted as mean (± SD). The single values for each mouse are listed in the table on the next page.

**%ID/g**	**60 min**	**SD**	**240 min**	**SD**
blood	1.19	1.01	0.23	0.42
brown adipose tissue	0.05	0.01	0.02	0.01
skin	0.12	0.04	0.04	0.03
brain	0.01	0.00	0.00	0.00
ovaries	0.15	0.08	0.01	0.01
uterus	0.13	0.03	0.02	0.01
pancreas	0.12	0.05	0.01	0.01
spleen	0.14	0.04	0.01	0.01
adrenals	0.22	0.11	0.03	0.03
kidneys	0.37	0.08	0.03	0.01
fat	0.21	0.29	0.01	0.01
muscle	0.06	0.01	0.02	0.02
heart	0.09	0.01	0.02	0.01
lung	0.23	0.09	0.03	0.01
thyroid	0.25	0.09	0.38	0.49
gall bladder*	39.9	-	24.7 / 24.6	-
liver	0.25	0.13	0.12	0.07
**femur**	**0.36**	**0.05**	**0.37**	**0.09**
**%ID**				
intestine	13.51	1.09	20.90	9.94
urine	79.26	1.31	73.24	11.75

*Only one (60 min) and two (240 min) values were determined.

%ID/g	60 min				240 min			
	**M1**	**M2**	**M3**	**M4**	**M5**	**M6**	**M7**	**M8**
blood	1.57	0.67	2.40	0.11	0.01	0.04	0.01	0.86
brown adipose tissue	0.05	0.04	0.07	0.06	0.02	0.04	0.01	0.01
skin	0.08	0.15	0.15	0.10	0.04	0.09	0.03	0.02
brain	0.01	0.02	0.02	0.01	0.00	0.01	0.00	0.01
ovaries	0.09	0.11	0.15	0.27	0.00	0.02	0.01	0.02
uterus	0.15	0.11	0.17	0.11	0.01	0.03	0.01	0.02
pancreas	0.12	0.13	0.17	0.05	0.01	0.02	0.00	0.01
spleen	0.16	0.08	0.15	0.16	0.01	0.02	0.01	0.02
adrenals	0.26	0.15	0.35	0.10	0.05	0.06	0.00	0.00
kidneys	0.35	0.29	0.41	0.48	0.03	0.05	0.03	0.03
fat	0.33	0.28	0.39	0.44	0.03	0.06	0.02	0.02
muscle	0.34	0.28	0.40	0.46	0.03	0.05	0.03	0.03
heart	0.08	0.64	0.06	0.05	0.00	0.01	0.00	0.01
lung	0.05	0.05	0.08	0.05	0.02	0.03	0.00	0.04
thyroid	0.08	0.10	0.11	0.08	0.02	0.04	0.01	0.01
gall bladder*	-	-	-	39.90	24.70		24.57	
liver	0.17	0.16	0.21	0.45	0.12	0.20	0.14	0.03
**femur**	**0.36**	**0.32**	**0.42**	**0.31**	**0.41**	**0.28**	**0.49**	**0.32**
**%ID**								
intestine	13.06	14.48	14.33	12.19	17.93	35.56	16.51	13.59
urine	79.39	79.04	77.70	80.89	77.28	55.74	79.26	80.67

Table S10: Ex vivo biodistribution data for Al[^{18}F]F-CHDT-Bn in SKH1 mice

For ex vivo biodistribution studies, two groups of SKH1 mice (n=3 for 60 min p.i. and n=4 for 240 min p.i., body weight of 25.8 ± 2.4 g) received 0.20 MBq of **Al[^{18}F]F-CHDT-Bn** and were sacrificed under desflurane anesthesia at 60 and 240 min p.i. Organs and tissues of interest were removed, weighed, and the activity was measured in a cross-calibrated well counter and dose calibrator. The decay corrected data were normalized to the amount of injected activity calculated from the activity of injection syringes before and after injection and expressed as percentage of injected activity per gram tissue (%ID/g tissue or %ID for intestine and urine). Values are quoted as mean (± SD). The single values for each mouse are listed in the table on the next page.

**%ID/g**	**60 min**	**SD**	**240 min**	**SD**
blood	0.03	0.02	0.02	0.01
brown adipose tissue	0.13	0.15	0.03	0.02
skin	0.08	0.07	0.09	0.09
brain	0.01	0.00	0.01	0.00
ovaries	0.10	0.05	0.06	0.07
uterus	0.11	0.07	0.04	0.02
pancreas	0.04	0.01	0.04	0.02
spleen	0.02	0.01	0.02	0.02
adrenals	0.07	0.00	0.14	0.06
kidneys	0.14	0.09	0.04	0.02
fat	0.12	0.06	0.10	0.09
muscle	0.13	0.07	0.02	0.01
heart	0.02	0.01	0.01	0.01
lung	0.05	0.03	0.02	0.01
thyroid	0.02	0.01	0.18	0.10
gall bladder*	74.10	98.99	108.14	64.83
liver	1.62	2.22	0.22	0.21
**femur**	**0.38**	**0.14**	**0.46**	**0.18**
**%ID**				
intestine	51.28	1.65	54.06	7.77
urine	39.20	2.12	32.68	1.81

%ID/g	60 min			240 min			
	**M1**	**M2**	**M3**	**M4**	**M5**	**M6**	**M7**
blood	0.03	0.05	0.02	0.03	0.02	0.01	0.01
brown adipose tissue	0.30	0.05	0.03	0.06	0.02	0.02	0.01
skin	0.04	0.03	0.16	0.03	0.02	0.22	0.09
brain	0.01	0.01	0.01	0.01	0.01	0.01	0.01
ovaries	0.16	0.07	0.07	0.16	0.06	0.01	0.02
uterus	0.03	0.12	0.17	0.06	0.05	0.02	0.03
pancreas	0.04	0.05	0.02	0.06	0.02	0.04	0.05
spleen	0.01	0.03	0.02	0.05	0.01	0.02	0.01
adrenals	-	0.07	0.07	0.23	0.08	0.15	0.11
kidneys	0.23	0.15	0.05	0.10	0.07	0.03	0.03
fat	0.14	0.16	0.05	0.04	0.05	0.02	0.02
muscle	0.17	0.16	0.05	0.07	0.06	0.02	0.03
heart	0.02	0.03	0.01	0.23	0.10	0.06	0.02
lung	0.03	0.08	0.02	0.02	0.03	0.02	0.01
thyroid	0.03	0.02	0.01	0.02	0.02	0.01	0.00
gall bladder*	209.17	-	87.22	99.01	202.46	67.04	64.07
liver	0.56	4.17	0.12	0.51	0.23	0.04	0.11
**femur**	**0.39**	**0.51**	**0.23**	**0.42**	**0.72**	**0.31**	**0.37**
**%ID**							
intestine	53.33	50.64	51.72	60.17	54.44	42.99	58.63
urine	40.96	38.19	40.96	31.92	32.60	30.99	35.21

Table S11: Tissue-specific time-activity courses of [^{18}F]PSMA-1007 in LNCaP xenograft mice

Data presented as ROI-averaged standardized uptake values [SUVmean] or proportion of initial activity dose [% ID] in renal (kidneys + urinary bladder) and hepatobiliary (gall bladder + liver + intestine) excretion systems; replicates (n = 4) stacked in columns.

**Time after injection [min]**		**0.8**	**2.1**	**5.5**	**11**	**20**	**38**	**55**	**83**	**113**
**Blood (heart content)**	[SUVmean]	7.06	3.65	2.01	1.40	0.90	0.64	0.45	0.33	0.25
		7.89	3.18	1.77	1.20	0.84	0.64	0.50	0.38	0.28
		7.88	3.50	1.85	1.25	0.78	0.57	0.40	0.29	0.21
		7.69	3.29	1.62	1.15	0.79	0.57	0.41	0.30	0.20
**Kidneys**	[SUVmean]	4.65	5.46	3.98	2.89	2.46	2.58	2.80	3.11	3.40
		6.11	6.22	6.25	6.36	6.79	7.09	7.21	7.21	6.65
		7.08	7.51	7.97	8.07	9.91	10.8	11.1	10.3	8.83
		7.15	7.78	8.12	8.13	9.00	8.56	8.13	8.08	7.86
**Liver**	[SUVmean]	3.34	2.16	1.37	1.07	0.78	0.60	0.48	0.37	0.29
		3.41	1.76	1.04	0.86	0.61	0.46	0.37	0.27	0.19
		3.28	1.86	1.19	0.92	0.64	0.48	0.37	0.27	0.19
		3.76	1.93	1.29	0.93	0.68	0.49	0.36	0.26	0.19
**Bone**	[SUVmean]	0.71	0.71	0.71	0.71	0.65	0.57	0.50	0.43	0.38
		0.44	0.41	0.53	0.53	0.47	0.37	0.30	0.26	0.21
		0.39	0.45	0.48	0.44	0.37	0.29	0.22	0.16	0.14
		0.53	0.47	0.49	0.45	0.41	0.30	0.19	0.15	0.11
**Parotid glands**	[SUVmean]	1.13	1.10	1.09	0.98	0.79	0.65	0.54	0.45	0.38
		1.59	1.32	1.33	1.18	1.06	0.96	0.83	0.73	0.63
		1.45	1.35	1.24	1.15	0.93	0.78	0.70	0.58	0.50
		1.19	1.12	1.26	1.13	1.07	0.97	0.88	0.80	0.73
**Tumor**	[SUVmean]	0.60	0.68	0.98	1.16	1.47	1.78	2.05	2.32	2.53
		0.71	0.85	1.33	1.62	2.32	3.00	3.67	4.21	4.55
		0.80	0.81	1.30	1.54	2.02	2.42	2.82	3.13	3.34
		0.74	0.79	0.99	1.09	1.42	1.66	1.96	2.25	2.44
**Muscle**	[SUVmean]	0.59	0.61	0.55	0.50	0.42	0.34	0.28	0.22	0.17
		0.63	0.55	0.57	0.52	0.46	0.40	0.35	0.27	0.21
		0.50	0.48	0.47	0.43	0.34	0.27	0.21	0.16	0.12
		0.83	0.63	0.62	0.51	0.45	0.36	0.28	0.22	0.17
**Renal**	[% ID]	9.48	11.1	19.4	26.5	37.0	46.1	53.7	60.6	66.3
		12.2	12.8	20.4	26.8	35.4	44.8	54.5	61.1	62.9
		9.61	10.5	18.7	25.2	35.1	42.0	48.0	53.3	55.1
		13.9	15.6	24.7	31.8	41.7	50.9	58.0	63.4	65.2
**Hepatobiliary**	[% ID]	24.5	16.0	10.2	8.22	6.41	5.40	4.55	3.88	3.21
		25.0	13.0	7.73	6.37	4.79	3.63	2.93	2.26	1.58
		24.7	14.3	9.31	7.28	5.17	4.00	3.13	2.37	1.78
		27.3	14.1	9.45	6.86	5.22	3.87	2.93	2.13	1.58

Table S12: Tissue-specific time-activity courses of Al[^{18}F]F-CHDT-PSMA-1 in LNCaP xenograft mice

Data presented as ROI-averaged standardized uptake values [SUVmean] or proportion of initial activity dose [% ID] in renal (kidneys + urinary bladder) and hepatobiliary (gall bladder + liver + intestine) excretion systems; replicates (n = 3) stacked in columns.

**Time after injection [min]**		**0.8**	**2.1**	**5.5**	**11**	**20**	**38**	**55**	**83**	**113**
**Blood (heart content)**	[SUVmean]	11.8	3.35	1.61	1.05	0.51	0.21	0.10	0.05	0.03
		8.84	2.83	1.30	0.80	0.36	0.16	0.11	0.05	0.03
		6.57	3.00	1.61	1.12	0.68	0.40	0.24	0.17	0.12
**Kidneys**	[SUVmean]	9.53	10.9	9.87	9.40	9.31	9.28	6.70	5.00	3.47
		7.53	8.26	8.05	8.09	7.85	7.01	6.07	4.18	2.61
		5.21	6.00	8.84	10.7	11.6	11.5	10.9	9.77	8.28
**Liver**	[SUVmean]	3.13	1.70	0.89	0.67	0.45	0.34	0.26	0.21	0.18
		4.33	1.85	0.98	0.67	0.43	0.27	0.21	0.18	0.15
		2.96	1.58	0.92	0.72	0.53	0.41	0.32	0.27	0.24
**Bone**	[SUVmean]	0.61	0.56	0.58	0.60	0.53	0.45	0.39	0.39	0.45
		0.63	0.61	0.72	0.71	0.59	0.52	0.42	0.52	0.42
		0.87	0.78	0.74	0.70	0.64	0.56	0.50	0.47	0.47
**Parotid glands**	[SUVmean]	1.13	1.15	1.22	1.01	0.77	0.59	0.44	0.28	0.21
		1.09	1.04	0.98	0.95	0.65	0.46	0.31	0.19	0.13
		1.12	1.09	1.06	0.96	0.79	0.60	0.46	0.35	0.28
**Tumor**	[SUVmean]	0.67	0.85	1.48	1.82	2.20	2.47	2.77	2.72	2.65
		0.90	1.02	1.63	2.05	2.53	2.79	3.03	2.97	2.91
		0.82	0.96	1.44	1.75	2.14	2.40	2.55	2.63	2.67
**Muscle**	[SUVmean]	1.13	0.71	0.44	0.32	0.16	0.10	0.04	0.03	0.02
		0.37	0.37	0.29	0.24	0.13	0.07	0.05	0.03	0.02
		0.63	0.60	0.54	0.43	0.29	0.18	0.12	0.08	0.05
**Renal**	[% ID]	18.3	21.7	36.8	48.2	64.2	74.5	81.5	84.2	85.4
		21.4	25.7	40.4	52.0	69.3	80.6	86.4	89.5	91.0
		11.3	12.9	19.7	26.2	38.0	48.8	57.1	62.7	65.9
**Hepatobiliary**	[% ID]	22.7	12.3	6.45	4.85	3.30	2.45	1.90	1.60	1.34
		31.3	13.5	7.16	4.87	3.13	2.01	1.68	1.45	1.22
		21.9	11.9	6.99	5.50	4.05	3.16	2.52	2.21	1.98

Table S13: Tissue-specific time-activity courses of Al[^{18}F]F-CHDT-PSMA-2 in LNCaP xenograft mice

Data presented as ROI-averaged standardized uptake values [SUVmean] or proportion of initial activity dose [% ID] in renal (kidneys + urinary bladder) and hepatobiliary (gall bladder + liver + intestine) excretion systems; replicates (n = 3) stacked in columns.

**Time after injection [min]**		**0.8**	**2.1**	**5.5**	**11**	**20**	**38**	**55**	**83**	**113**
**Blood (heart content)**	[SUVmean]	8.95	2.41	0.90	0.48	0.19	0.09	0.05	0.03	0.03
		8.35	1.76	0.66	0.35	0.16	0.09	0.05	0.04	0.03
		7.11	1.93	0.83	0.50	0.27	0.17	0.11	0.08	0.07
**Kidneys**	[SUVmean]	7.27	7.22	5.07	4.34	3.80	3.36	3.01	2.30	1.78
		7.00	6.26	4.34	3.79	3.10	2.63	2.19	1.53	1.22
		4.06	4.61	4.70	4.22	3.79	3.37	3.00	2.62	2.29
**Liver**	[SUVmean]	6.53	7.16	7.91	5.96	2.50	0.76	0.17	0.06	0.03
		5.43	5.83	6.07	4.97	2.40	0.76	0.22	0.08	0.06
		5.35	5.81	6.68	6.16	4.09	2.15	0.96	0.43	0.20
**Bone**	[SUVmean]	0.63	0.44	0.41	0.41	0.37	0.36	0.28	0.33	0.39
		0.67	0.58	0.47	0.43	0.44	0.48	0.52	0.64	0.67
		0.62	0.56	0.49	0.45	0.44	0.41	0.42	0.44	0.47
**Parotid glands**	[SUVmean]	1.23	0.89	0.66	0.49	0.33	0.24	0.16	0.14	0.11
		0.90	0.84	0.53	0.42	0.25	0.17	0.12	0.09	0.08
		1.04	0.91	0.68	0.48	0.31	0.21	0.16	0.13	0.11
**Tumor**	[SUVmean]	0.68	0.72	0.97	1.07	1.17	1.23	1.24	1.23	1.23
		0.74	0.77	0.94	1.08	1.15	1.24	1.25	1.19	1.19
		0.61	0.61	0.74	0.81	0.90	0.95	0.97	0.98	0.98
**Muscle**	[SUVmean]	0.64	0.43	0.30	0.19	0.08	0.05	0.03	0.02	0.02
		0.31	0.27	0.17	0.11	0.06	0.03	0.02	0.01	0.01
		0.53	0.43	0.30	0.22	0.12	0.08	0.05	0.04	0.03
**Renal**	[% ID]	11.7	13.1	20.9	25.2	28.7	30.1	29.8	24.0	21.1
		12.2	11.6	16.2	21.1	24.7	24.9	27.6	28.7	27.8
		7.74	8.69	12.6	15.7	19.7	22.8	24.9	26.3	27.1
**Hepatobiliary**	[% ID]	48.7	52.9	58.4	45.6	32.6	28.5	36.6	42.2	47.1
		41.8	44.5	47.1	45.2	44.1	46.0	50.1	51.5	52.4
		40.0	43.1	50.6	51.4	46.9	42.5	40.5	40.2	39.4

Table S14: Tissue-specific time-activity courses of Al[^{18}F]F-CHDT-PSMA-3 in LNCaP xenograft mice

Data presented as ROI-averaged standardized uptake values [SUVmean] or proportion of initial activity dose [% ID] in renal (kidneys + urinary bladder) and hepatobiliary (gall bladder + liver + intestine) excretion systems; replicates (n = 3) stacked in columns.

**Time after injection [min]**		**0.8**	**2.1**	**5.5**	**11**	**20**	**38**	**55**	**83**	**113**
**Blood (heart content)**	[SUVmean]	7.62	3.13	1.60	1.06	0.61	0.35	0.19	0.11	0.08
		10.2	4.24	2.07	1.24	0.72	0.38	0.19	0.11	0.06
		11.0	4.22	1.92	1.22	0.66	0.34	0.19	0.10	0.07
**Kidneys**	[SUVmean]	3.81	4.57	5.00	4.45	3.85	3.28	2.77	2.20	1.65
		6.35	7.13	6.87	6.10	5.27	4.61	3.76	2.79	2.02
		7.57	8.11	7.36	6.62	6.09	5.11	3.97	2.84	1.90
**Liver**	[SUVmean]	3.53	3.05	2.92	2.75	1.98	1.19	0.66	0.37	0.22
		4.24	3.50	3.53	3.38	2.14	1.09	0.48	0.25	0.13
		4.54	3.33	3.04	2.67	1.72	0.88	0.40	0.20	0.11
**Bone**	[SUVmean]	0.80	0.77	0.72	0.68	0.57	0.50	0.47	0.44	0.44
		0.69	0.58	0.60	0.56	0.39	0.34	0.27	0.18	0.26
		0.57	0.50	0.58	0.44	0.35	0.28	0.19	0.16	0.20
**Parotid glands**	[SUVmean]	1.19	1.18	1.03	0.83	0.54	0.34	0.21	0.14	0.09
		1.30	1.47	1.22	1.07	0.69	0.46	0.32	0.21	0.14
		1.22	1.53	1.25	1.10	0.76	0.49	0.35	0.21	0.13
**Tumor**	[SUVmean]	0.61	0.70	1.01	1.19	1.44	1.62	1.75	1.84	1.89
		0.67	0.68	1.16	1.51	1.94	2.35	2.65	2.80	2.87
		0.86	1.09	1.69	2.20	2.85	3.43	3.88	4.12	4.06
**Muscle**	[SUVmean]	0.82	0.76	0.54	0.41	0.25	0.15	0.09	0.06	0.04
		0.66	0.52	0.43	0.31	0.19	0.11	0.05	0.03	0.02
		0.79	0.42	0.42	0.35	0.24	0.14	0.10	0.06	0.03
**Renal**	[% ID]	6.31	7.45	14.6	21.1	31.0	38.1	43.5	47.5	50.0
		9.86	11.3	19.1	25.6	35.5	44.0	49.7	51.3	51.4
		13.7	15.5	23.9	30.4	40.0	47.9	52.9	56.1	56.9
**Hepatobiliary**	[% ID]	27.3	23.6	22.4	21.9	19.8	19.8	20.5	19.5	18.8
		32.3	26.7	27.0	28.7	28.3	29.0	29.7	29.8	29.2
		33.7	24.9	22.8	22.1	22.4	25.8	29.5	30.8	31.0

Chemistry

Intermediate compounds

Compound 1

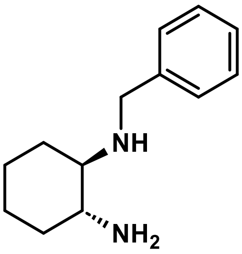

(±)-*trans*-1,2-Diaminocyclohexane (11.3 mL, 94.2 mmol, 10 eq.) was dissolved in CH_3OH (30 mL) under argon atmosphere. To this solution, benzaldehyde (0.95 mL, 9.4 mmol, 1 eq.) was added dropwise under vigorous stirring. The reaction mixture was stirred for 2 h at 65°C. Subsequently, the mixture was cooled on ice and $NaBH_4$ (356 mg, 9.4 mmol, 1 eq.) was added in portions and the resulting mixture was stirred overnight (without argon). The solvent was removed *in vacuo* and the residue was taken up in sat. $NaHCO_3$ (25 mL). The aqueous phase was extracted with CH_2Cl_2 (3×25 mL) and the combined organic phases were dried over Na_2SO_4 and the solvent was removed *in vacuo*. The crude product was purified by column chromatography using aluminium oxide 60 and CH_2Cl_2/CH_3OH/triethylamine (97:3+0.1, v/v/v). The product containing fractions were combined and the solvent was removed *in vacuo*. Compound **1** (1.76 g, 92%) was obtained as a yellowish oil. R_f = 0.33 (aluminium oxide basic, CH_2Cl_2/CH_3OH/triethylamine, 97/3+0.1); **^{1}H-NMR** ($CDCl_3$): δ= 7.38–7.19 (m, 5H, 5×H_{Phenyl}), 3.95 (d, ^2J=13.0 Hz, 1H, C*H*H-phenyl), 3.69 (d, ^2J=13.0 Hz, 1H, CH*H*-phenyl), 2.45–2.34 (m, 1H), 2.19–2.06 (m, 2H), 1.93–1.85 (m, 1H), 1.75–1.63 (m, 1H), 1.35–0.94 (m, 5H); **^{13}C-NMR** ($CDCl_3$): δ=141.06 (C-1 phenyl), 128.50 (2×CH phenyl), 128.29 (2×CH phenyl), 126.96 (C-4 phenyl), 63.31, 55.49, 51.19, 35.96, 31.48, 25.38, 25.35.

Compound 2

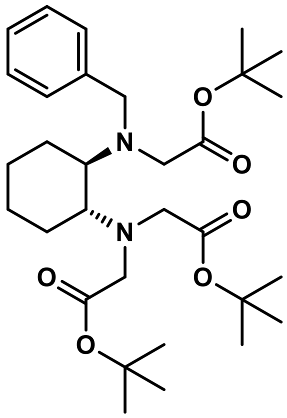

Compound **1** (824 mg, 4.03 mmol, 1 eq.) was dissolved in CH_2Cl_2 (5 mL) under argon atmosphere. To this solution, DIPEA (2.82 mL, 16.1 mmol, 4 eq.) and *tert*-butyl bromoacetate (3.15 g, 16.1 mmol, 4 eq.) were added dropwise and alternately. Subsequently, additional CH_2Cl_2 (5 mL) was added. The mixture was stirred overnight. The solvent was removed *in vacuo* and the residue was taken up in water (50 mL). The aqueous phase was extracted with CH_2Cl_2 (3×20 mL) and the combined organic phases were again washed with water (20 mL) and dried over Na_2SO_4 followed by removal of the solvent *in vacuo*. The crude product was purified by flash chromatography using a gradient of ethyl acetate/hexane from 0/100 to 30/70. The product containing fractions were combined and the solvent was removed *in vacuo*. Compound **2** (1.83 g, 83%) was obtained as a light-yellow oil. **^{1}H-NMR** ($CDCl_3$): δ=7.47–7.40 (m, 2H, 2×H_{phenyl}), 7.31–7.17 (m, 3H, 3×H_{phenyl}), 4.00 (d, ^2J=13.4 Hz, 1H, C*H*H-phenyl), 3.68 (d, ^2J=13.4 Hz, 1H, CH*H*-phenyl), 3.50–3.38 (m, 4H, 2×CH_2-N), 3.39 (d, ^2J=16.7 Hz, 1H, C*H*H-N), 3.26 (d, ^2J=16.8 Hz, 1H, CH*H*-N), 2.75–2.65 (m, 1H, CH), 2.59–2.49 (m, 1H, CH), 2.11–1.96 (m, 2H), 1.71–1.63 (m, 2H), 1.45–1.40 (m, 27H, 9×CH_3), 1.22–0.99 (m, 4H); **^{13}C-NMR** ($CDCl_3$): δ=172.29 (CO), 171.85 (2×CO), 140.35 (C-1 phenyl), 129.43 (2×CH phenyl), 128.17 (2×CH phenyl), 126.87 (C-4 phenyl), 80.40 (2×$C_{quart.}$ *t*Bu), 80.37 ($C_{quart.}$ *t*Bu), 63.62, 60.99, 54.86, 53.36 (2×C), 52.87, 29.68, 28.36, 28.30/28.28/28.24/28.12 (CH_3 signals), 26.02, 25.88.

Compound 3

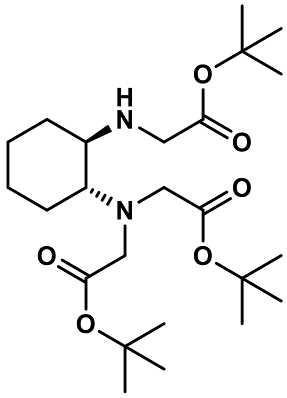

Compound **2** (1.05 g, 1.92 mmol, 1 eq.) was dissolved in CH_3OH (55 mL) in a high-pressure laboratory autoclave followed by the addition of ammonium formate (0.61 g, 9.59 mmol, 5 eq.). Subsequently, 10% Pd/C (0.58 mg) was added and the autoclave was sealed. The mixture was stirred for 2 h at 65°C. After that, the mixture was cooled to room temperature and the autoclave was carefully opened. The methanolic solution was filtered through a glass fibre microfilter and subsequently the solvent was removed *in vacuo*. The residue was taken up in CH_2Cl_2 und the organic phase was washed with water (3×20 mL). The organic phase was dried over Na_2SO_4 and the solvent was removed *in vacuo*. Compound **3** (0.82 g, 93%) was obtained as clear oil and was used without further purification. **^{1}H-NMR** ($CDCl_3$): δ=3.84 (d, ^2J=16.9 Hz, 1H, C*H*H-N), 3.58–3.28 (m, 5H, 2×CH_2-N, CH*H*-N), 2.92–2.78 (m, 1H, CH-N), 2.66–2.54 (m, 1H, CH-N), 2.11–2.00 (m, 2H), 1.85–1.71 (m, 2H), 1.70–1.57 (m, 1H, CH*H*), 1.50–1.05 (m, 32H, 9×CH_3, C*H*H, 2×CH_2); **^{13}C-NMR** ($CDCl_3$): δ=172.17 (2×CO), 167.05 (CO), 83.25 ($C_{quart.}$ *t*Bu),, 81.69 (2×$C_{quart.}$ *t*Bu), 64.95, 58.82, 45.65, 28.24/28.15 (CH_3 signals), 27.47, 24.88, 24.36. Signals for 3×CH/CH_2 no visible.

Compound 4

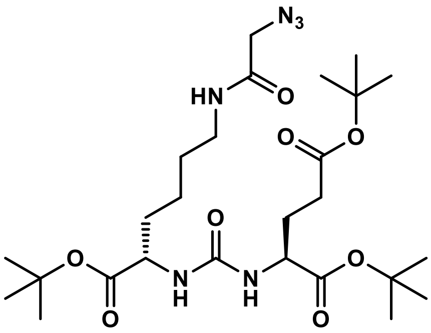

Di-*tert*-butyl (((*S*)-6-amino-1-(*tert*-butoxy)-1-oxohexan-2-yl)carbamoyl)-L-glutamate (83 mg, 0.17 mmol, 1 eq., prepared as described by Xiao *et al.*[1]) was dissolved in CH_2Cl_2/DMF (10:1, v/v, 3.3 mL) followed by the addition azidoacetic acid (25.4 µL, 0.34 mmol, 2 eq.), DIPEA (118.5 µL, 0.34 mmol, 2 eq.) and PyBOP (177 mg, 0.34 mmol, 2 eq.). The formation of the desired product was followed by UPLC-DAD-MS analysis. After stirring for 3.5 h, the solvent was evaporated *in vacuo*. The crude product was purified by RP-HPLC. The product-containing fractions were collected and after lyophilization compound **A** was obtained as white solid (47 mg, 48%). **^{1}H-NMR** ($CDCl_3$): δ=6.57 (broad s, 1H, $N_\varepsilon H$), 5.21 (broad s, 2H, 2×NH), 4.31 (s, 2H, 2×C_α), 4.05–3.92 (m, 2H, CH_2-N_3), 3.41–3.18 (m, 2H, $C_\varepsilon H_2$), 2.41–1.23 (m, 37H, 9×CH_3, 2×$C_\beta H_2$, 2×$C_\gamma H_2$, $C_\delta H_2$); **^{13}C-NMR** ($CDCl_3$): δ=172.60 (CO), 172.45 (CO), 172.38 (CO), 167.21 (CO), 157.20 (NHCONH), 82.39 ($C_{quart.}$ of *tert*-butyl), 82.02 ($C_{quart.}$ of *tert*-butyl), 80.81 ($C_{quart.}$ of *tert*-butyl), 53.44 (C_α), 53.25 (C_α), 52.72 (CH_2-N_3), 39.01 (C_ε), 32.51, 31.72, 28.83, 28.39, 28.22, 28.15, 22.41; m/z calculated for $C_{26}H_{47}N_6O_8$: 571.34 $[M+H]^+$, found: 571.0.

Compound 5

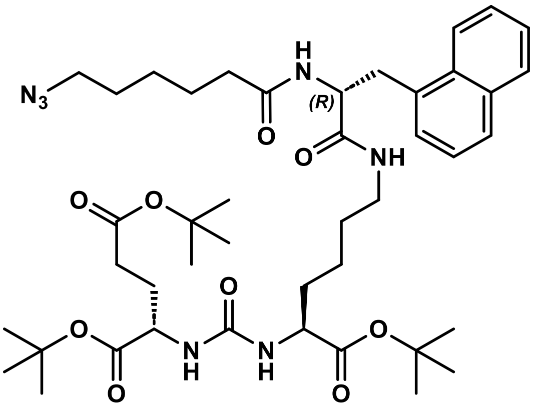

Di-*tert*-butyl (((*RS*)-6-((*R*)-2-amino-3-(naphthalen-1-yl)propanamido)-1-(*tert*-butoxy)-1-oxo-hexan-2-yl)carbamoyl)-L-glutamate (200 mg, 0.35 mmol, 1 eq., synthesized according to ref. [2]) and 6-azidohexanoic acid (60 mg, 0.38 mmol, 1.1 eq.) were dissolved in DMF (15 mL). To this solution, DIPEA (66 uL, 0.38 mmol, 1.1 eq.), EDC (73 mg, 0.38 mmol, 1.1 eq.) and HOBt (52 mg, 0.38 mmol, 1.1 eq.) were added and the mixture was stirred overnight. DMF was removed *in vacuo* and the residue was taken up in CH_2Cl_2 (20 mL), the organic phase was washed with sat. $NaHCO_3$ (2×10 mL) and brine (10 mL), dried over Na_2SO_4 and the solvent was removed *in vacuo*. The crude product was purified by column chromatography with a gradient of CH_3OH/CH_2Cl_2 from 0/100 → 4/96. The product-containing fraction were combined and the solvent was removed *in vacuo*. Compound **5** (210 mg, 74%) was obtained as oily residue. **^{1}H NMR** ($CDCl_3$): δ=8.41 (d, ^3J=7.7 Hz, 1H, CH 1-naphthyl), 7.95 (d, ^3J=8.1 Hz, 1H, CH 1-naphthyl), 7.66–7.51 (m, 2H, 2×CH 1-naphthyl), 7.43–7.30 (m, 2H, 2×CH 1-naphthyl), 6.59 (d, ^3J=7.3 Hz, 1H, N_αH 1-Nal), 5.80 (broad s, 1H, N_εH), 4.90 (broad s, 1H, NH), 4.84–4.74 (m, 1H, C_αH 1-Nal), 4.45 (broad s, 1H, NH), 4.18 (s, 2H, 2×C_αH), 3.74 (dd, ^2J=13.4 Hz, ^3J=5.3 Hz, 1H, $C_\beta H$H 1-Nal), 3.32–3.17 (m, 4H, C_βH*H* 1-Nal, C_ε*H*H Lys, CH_2-N_3), 2.75–2.64 (m, 1H, C_εH*H* Lys), 2.35–2.19 (m, 4H), 2.10–1.97 (m, 1H, C*H*H), 1.79–1.52 (m, 6H, 2×CH*H*, 2×CH_2), 1.51–1.29 (m, 30H, 9×CH_3, CH_2, *CHH*), 1.15–1.08 (m, 2H, CH_2), 0.87–0.81 (m, 2H, CH_2); **^{13}C NMR** ($CDCl_3$): δ=172.90 (CO), 172.64 (CO), 172.50 (CO), 172.30 (CO), 171.16 (CO), 157.03 (NHCONH), 133.96, 133.41, 132.26, 128.82 (CH 1-naphthyl), 128.39 (CH 1-naphthyl), 128.01 (CH 1-naphthyl), 126.91 (CH 1-naphthyl), 126.22 (CH 1-naphthyl), 125.90 (CH 1-naphthyl), 124.69 (CH 1-naphthyl), 82.27 ($C_{quart.}$ of *tert*-butyl), 81.74 ($C_{quart.}$ of *tert*-butyl), 80.77 ($C_{quart.}$ of *tert*-butyl), 54.69 (C_α 1-Nal), 53.23 (C_α), 53.13 (C_α), 51.38 (C-N_3), 38.29 (C_ε), 37.00 (C_β 1-Nal), 36.39, 32.10, 31.43, 28.77, 28.27/28.19/28.12 (CH_3 signals, 1×CH_2), 28.02, 26.46, 25.19, 21.35.

Compound 6

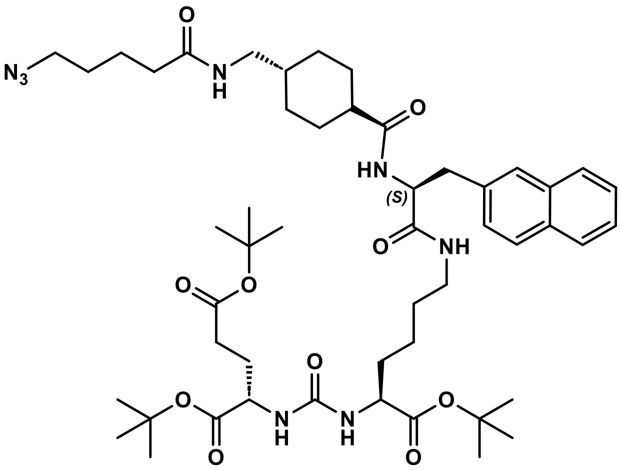

Compound **6** was synthesized as described previously.[2]

Tri-*tert*-butyl (3*S*,10*RS*,14*S*)-1-((1*s*,4*R*)-4-(aminomethyl)cyclohexyl)-3-(naphthalen-2-ylmethyl)-1,4,12-trioxo-2,5,11,13-tetraazahexadecane-10,14,16-tricarboxylate (157 mg, 0.19 mmol, 1 eq.)[2] and 5-azidopentanoic acid (32 mg, 0.23 mmol, 1.2 eq.) were dissolved in DMF (15 mL). To this solution, DIPEA (39 µL, 0.23 mmol, 1.2 eq.), EDC (43 mg, 0.23 mmol, 1.2 eq.) and HOBt (38 mg, 0.23 mmol, 1.2 eq.) were added and the mixture was stirred overnight. DMF was removed *in vacuo* and the residue was taken up in CH_2Cl_2 (20 mL), the organic phase was washed with sat. $NaHCO_3$ (2×10 mL) and brine (10 mL), dried over Na_2SO_4 and the solvent was removed *in vacuo*. The crude product was purified by column chromatography with a gradient of CH_3OH/CH_2Cl_2 from 0/100 → 5/95. The product-containing fraction were combined and the solvent was removed *in vacuo*. Compound **6** (130 mg, 72%) was obtained as oily residue. **^{1}H NMR** ($CDCl_3$): δ=7.71–7.62 (m, 2H, 2×CH 2-naphthyl), 7.57 (d, ^3J=8.1 Hz, 1H, CH 2-naphthyl), 7.45 (t, ^3J=7.2 Hz, 1H, CH 2-naphthyl), 7.38 (t, ^3J=7.4 Hz, 1H, CH 2-naphthyl), 7.22 (broad s, 1H, CH 2-naphthyl), 7.13 (d, ^3J=8.6 Hz, 1H, CH 2-naphthyl), 6.11 (broad s, 1H, N_αH 2-Nal), 5.51 (broad s, 1H, N_εH Lys), 5.15 (broad s, 1H, C_αH 2-Nal), 4.62 (broad s, 1H, C_αH), 4.29 (broad s, 1H, C_αH), 3.59–2.86 (m, 8H, $C_\beta H_2$ 2-Nal, CH_2-N_3, $C_\varepsilon H_2$, CH_2-N), 2.51–2.30 (m, 4H), 2.26–2.12 (m, 4H), 1.97–0.65 (m, 45H), 3×NH are not visible; **^{13}C NMR** ($CDCl_3$): δ=177.21 (CO), 174.93 (CO), 172.53 (3×CO), 157.68 (NHCONH), 134.99, 133.46, 132.32, 128.20/127.98/127.89/127.43/127.11/126.08/125.54 (7×CH 2-naphthyl), 82.62/81.09/80.55 (3× $C_{quart.}$ of *tert*-butyl), 54.74 (C_α 2-Nal), 53.34 (C_α), 52.47 (C_α), 51.29 (C-N_3), 45.58 (C_ε), 44.56 (CH-NH), 40.41 (C_ε), 39.96 (CH_2-N), 39.26 (C_β 2-Nal), 37.14 (CH), 36.08, 33.30, 31.78, 30.25, 30.12, 29.84, 29.58, 29.41, 28.90, 28.54, 28.30/28.26/28.18 (CH_3 signals), 24.81, 23.05, 23.00, 22.11.

Compound 7

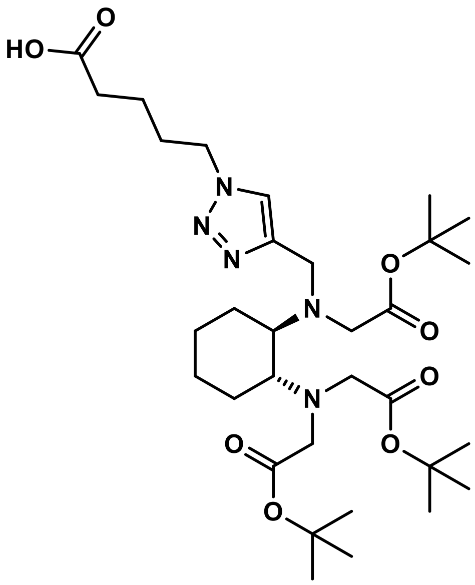

To a solution of **(±)-CHDA-*t*Bu** (20 mg, 40 µmol, 1 eq.) and 5-azidopentanoic acid (11 mg, 80 µmol, 2 eq.) in *tert*-butyl alcohol (750 µL), TBTA (1.3 mg, 2.5 µmol, 0.063 eq.) was added. After brief stirring, 1 M sodium ascorbate (50 µL) and 0.1 M $CuSO_4$ (100 µL) aqueous solutions were added and the gentle mixing was continued at room temperature for 24 h. The reaction mixture was then diluted with ethyl acetate (10 mL) and extracted three times with aqueous 0.05 M EDTA (3 x 5 mL) and then 1 M NH_4OH (3 x 5 mL) solutions to remove copper salts. The organic phase was dried (Na_2SO_4), filtered and evaporated under reduced pressure to afford the crude product, which was used without further purification. Yield*:* 20.1 mg (31 µmol; 77%). **^{1}H NMR** ($CDCl_3$): δ=8.01 (s, 1H, H-5 triazole), 4.41–4.26 (m, 2H, CH_2), 3.97 (d, ^2J=14.6 Hz, 2H, C*H*H), 3.78 (d, ^2J=13.9 Hz, 1H, CH*H*), 3.52–3.20 (m, 6H, 3×CH_2), 2.71–2.53 (m, 2H), 2.47–2.29 (m, 2H), 2.08–1.90 (m, 4H, 2×CH_2), 1.77–1.61 (m, 4H, 2×CH_2), 1.51–1.34 (m, 27H, 9×CH_3), 1.20–1.00 (m, 4H, 2×CH_2); **^{13}C NMR** ($CDCl_3$): δ=172.02 (2×CO), 171.87 (CO), 124.36 (C-5 triazole), 80.93 (3×$C_{quart.}$ *t*Bu), 67.22 (3×C), 63.16, 53.08, 49.98, 45.56, 29.70, 28.27/28.22 (CH_3 signals), 25.93, 25.68, signals for 3×CH_2 and C-4 triazole are not visible; m/z calculated for $C_{32}H_{56}N_5O_8$: 638.41 $[M+H]^+$, found: 638.0.

Compound 8

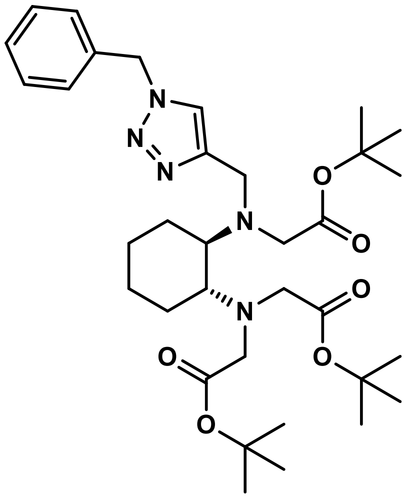

The preparation of compound **8** was performed as described for compound **7** (starting materials: **(±)-CHDA-*t*Bu** and benzyl azide). Yield*:* 640 mg (1.02 mmol; 99%). **^{1}H-NMR** (600 MHz, $CDCl_3$): δ=7.94 (s, 1H, H-5 triazole), 7.34–7.27 (m, 5H, phenyl), 5.52 (d, ^{2}J=14.9 Hz, 1H, C*H*H-phenyl), 5.46 (d, ^{2}J=14.9 Hz, 1H, CH*H*-phenyl), 4.00 (d, ^{2}J=14.3 Hz, 1H, C*H*H-triazole), 3.76 (d, ^{2}J=14.2 Hz, 1H, CH*H*-triazole), 3.48 (d, ^{2}J=16.8 Hz, 2H, 2×C*H*H-N), 3.37 (d, ^{2}J=16.8 Hz, 2H, 2×CH*H*-N), 3.31 (d, ^{2}J=16.9 Hz, 1H, C*H*H-N), 3.25 (d, ^{2}J=16.9 Hz, 1H, CH*H*-N), 2.72–2.66 (m, 1H, CH-N), 2.60–2.55 (m, 1H, CH-N), 2.05–1.91 (m, 2H), 1.75–1.62 (m, 2H), 1.40 (s, 18H, 6×CH_3), 1.36 (s, 9H, 3×CH_3), 1.15–1.06 (m, 3H), signal for 1×C*H*H is not identifiable; **^{13}C-NMR** (600 MHz, $CDCl_3$): δ=171.78 (3×CO), 148.38 (C-4 triazole), 135.47 (C-1 phenyl), 129.04 (2×CH phenyl), 128.50 (C-4 phenyl), 128.14 (2×CH phenyl), 124.33 (C-5 triazole), 80.40 (3×$C_{quart.}$ *t*Bu), 63.40 (CH-N), 62.15 (CH-N), 54.12, 53.25, 53.08, 45.55, 29.07, 28.29/28.21 (CH_3 signals), 27.26, 26.05, 25.85; m/z calculated for $C_{34}H_{54}N_5O_6$: 628.41 $[M+H]^+$, found: 628.4.

Compound 9 (2×TFA)

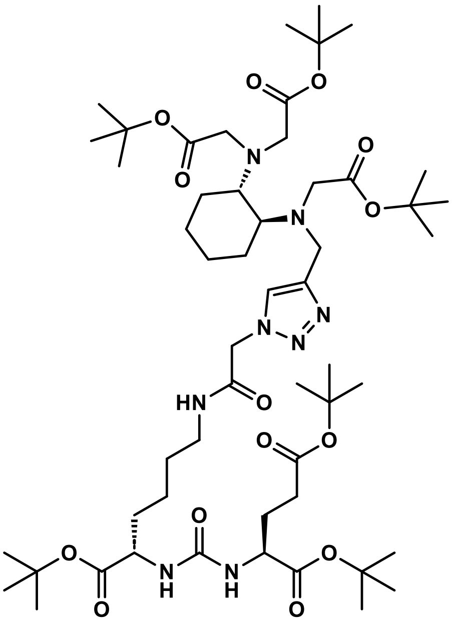

(±)-CHDT-*t*Bu (33.5 mg, 0.068 mmol, 1 eq.) was dissolved in *tert*-butanol (500 µL) and the mixture was added to compound **4** (38.7 mg, 0.068 mmol, 1 eq.). $CuSO_4$ (170 µL, 0.1 M in water) was added to this mixture followed by the addition of sodium ascorbate (85 µL, 1 M in water). The formation of the desired product was followed by UPLC-DAD-MS analysis. After stirring for 15 h, ethyl acetate (10 mL) was added and the organic phase was washed with 0.05 M EDTA in water (3×5 mL) and brine (1×5 mL) and the solvent was removed *in vacuo*. The crude product was purified by RP-HPLC. The product-containing fractions were collected and after lyophilization compound **9** was obtained as white solid (41 mg, 43%). m/z calculated for $C_{53}H_{93}N_8O_{14}$: 1065.68 $[M+H]^+$, found: 1065.5.

Compound 10

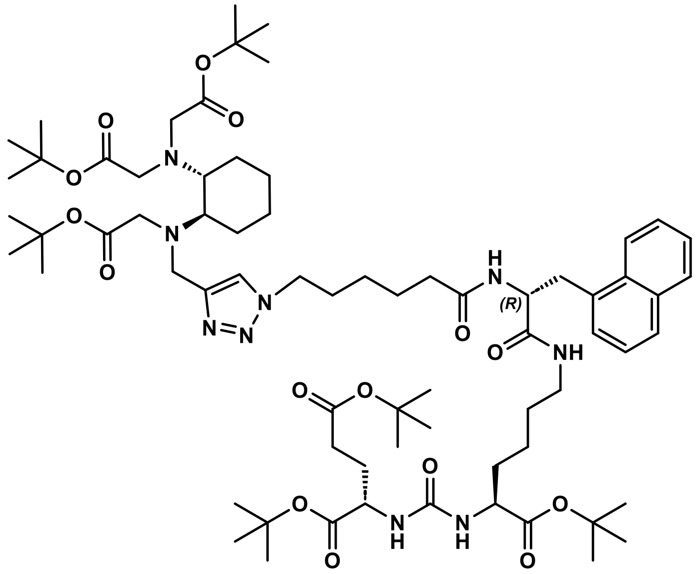

Compound **5** (200 mg, 0.24 mmol, 1 eq.) and **(±)-CHDA-*t*Bu** (132 mg, 0.24 mmol, 1 eq.) were dissolved in H_2O/MeOH (10 mL, 1/1). $CuCl_2$ (20 mg) and sodium ascorbate (30 mg) were added to the reaction mixture. The mixture was stirred at 60°C for 2 h and the reaction followed by thin layer chromatography (TLC). Afterwards, the solvents were removed, the residue was dissolved in CH_2Cl_2 and the organic phase was extracted three times (2×sat. $NaHCO_3$, 1×brine) and then dried over sodium sulfate. The crude product was purified column chromatography (ethyl acetate/CH_3CH_2OH, 90/10) and compound **10** was obtained as oily residue (213 mg, 67%). m/z calculated for $C_{70}H_{112}N_9O_{15}$: 1318.83 $[M+H]^+$, found: 1318.0.

Compound 11

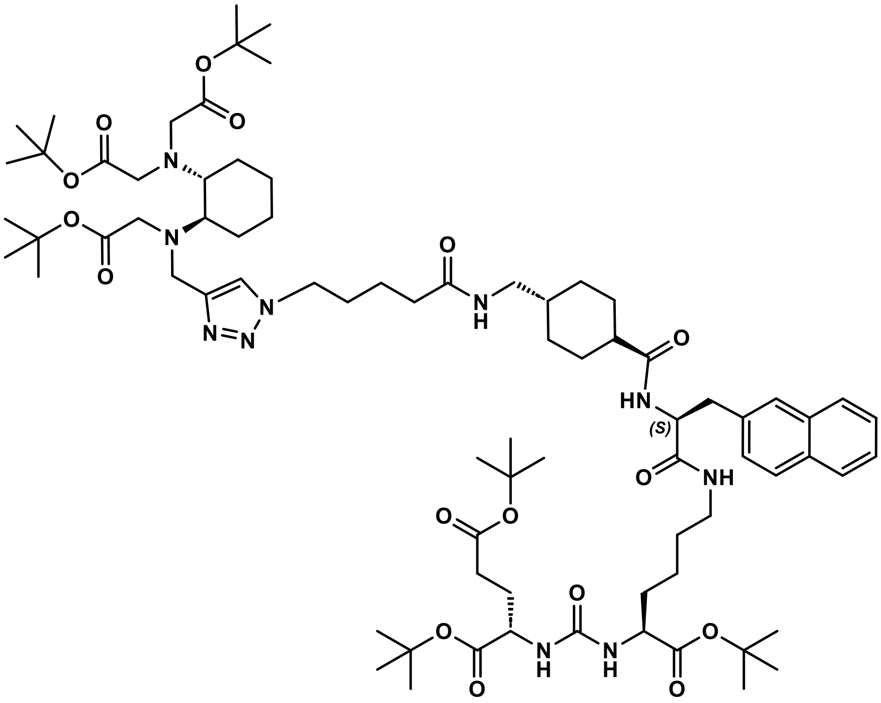

Compound **6** (25 mg, 0.025 mmol) and compound **(±)-CHDA-*t*Bu** (15 mg, 0.03 mmol) were dissolved in 10 mL H_2O/MeOH (1/1). $CuCl_2$ (10 mg) and sodium ascorbate (50 mg) were added to the reaction mixture. The mixture was stirred at 40°C for three hours and the reaction followed by thin layer chromatography (TLC). Afterwards, the solvents were removed, the residue was dissolved in CH_2Cl_2 and the organic phase was extracted five times (1 x H_2O, 2 x metabisulfite, 1 x H_2O) and then dried over sodium sulfate. The crude product was purified by automated flash chromatography (CH_2Cl_2/MeOH 0-15%) and compound **11** was obtained as yellowish oil (10 mg, 28%). m/z calculated for $C_{77}H_{123}N_{10}O_{16}$: 1443.91 $[M+H]^+$, found: 1444.7.

Final compounds

(±)-CHDA-*t*Bu

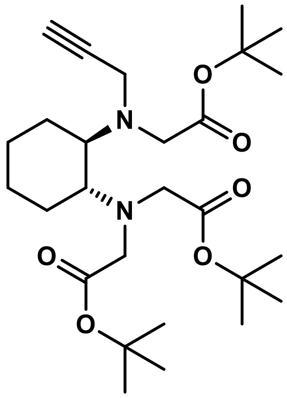

Compound **3** (1.89 g, 4.14 mmol, 1 eq.) was dissolved in CH_3CN (35 mL) under argon atmosphere. DIPEA (3.6 mL, 20.7 mmol, 5 eq.) dissolved in CH_3CN (13.4 mL) and propargyl bromide (0.46 mL, 4.14 mmol, 1 eq.) dissolved in CH_3CN (6.8 mL) were added dropwise in parallel to the solution of compound **3**. The reaction mixture was stirred overnight. The solvent was removed *in vacuo*. The residue was taken up in *tert*-butyl methyl ether and the solution was filtrated. The organic phase was washed with water (3×30 mL), dried over Na_2SO_4 and the solvent was removed *in vacuo*. The crude product was purified by a two times flash chromatography (isocratic $CHCl_3$). The product-containing fraction were combined and the solvent was removed *in vacuo*. Compound **3** (1.19 g, 58%) was obtained as brownish oil. **^{1}H NMR** ($CDCl_3$): δ=3.76–3.57 (m, 2H, CH_2-N), 3.50 (s, 4H, 2×CH_2-N), 3.47–3.33 (m, 2H, CH_2-N), 2.77–2.60 (m, 2H, 2×CH-N), 2.17 (t, ^4J=2.4 Hz, 1H, CH≡C), 2.07–1.97 (m, 2H, CH_2), 1.71–1.63 (m, 2H, CH_2), 1.50–1.43 (m, 27H, 9×CH_3), 1.32–1.04 (m, 4H, 2×CH_2); **^{13}C NMR** ($CDCl_3$): δ=171.90 (2×CO), 171.38 (CO), 82.03 (*C*≡CH), 80.72, 80.45 (2×$C_{quart.}$ *t*Bu), 72.38 (C≡*C*H), 63.05, 62.99, 53.87 (2×C), 52.49, 40.17, 30.08, 28.44, 28.31/28.28 (CH_3 signals), 25.82, 25.75; m/z calculated for $C_{27}H_{47}N_2O_6$: 495.34 $[M+H]^+$, found: 495.0.

CHDT-Pe (×2TFA)

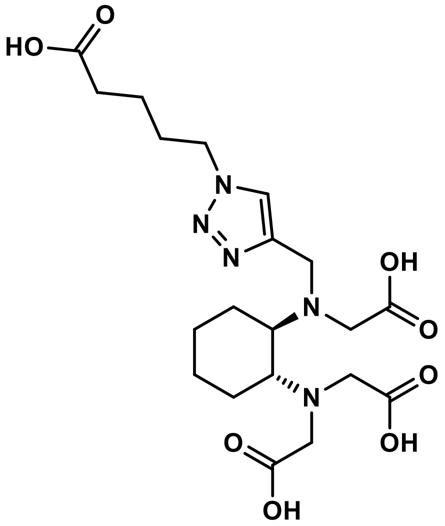

Compound **7** (10 mg, 15 µmol, 1 eq.) was dissolved in a mixture of TFA (450 µL) and water (50 µL) and stirred for 5 h. Then, the reaction mixture was added dropwise to ice-cooled Et_2O (10 mL). The supernatant was decanted and the precipitate was washed twice with cold Et_2O. Yield*:* 5.4 mg (9 µmol; 60%). **^{1}H NMR** (D_2O): δ=8.25 (s, 1H, H-5 triazole), 4.64–4.53 (m, 1H), 4.49 (t, ^3J=7.0 Hz, 3H), 3.91 (d, ^2J=16.9 Hz, 1H), 3.77–3.61 (m, 1H), 3.49–2.94 (m, 4H), 2.41 (t, ^3J=7.4 Hz, 2H), 2.27 (d, ^2J=11.8 Hz, 1H), 2.17–2.05 (m, 1H), 2.03–1.77 (m, 5H), 1.63–1.46 (m, 3H), 1.44–1.22 (m, 4H); **^{13}C NMR** (D_2O): δ=178.09 (CO), 162.85 (q, $^2J_{C,F}$=35.7 Hz, CO TFA), 116.21 (q, $^1J_{C,F}$=291.1 Hz, CF_3 TFA), 64.09, 50.27, 32.81 (2×C), 28.49 (2×C), 24.16, 23.62, 23.49, 20.95 (2×C), signals for 3×CH/CH_2 and C-4,5 triazole are not visible; m/z calculated for $C_{20}H_{32}N_5O_8$: 470.22 $[M+H]^+$, found: 471.0.

CHDT-Bn (×2TFA)

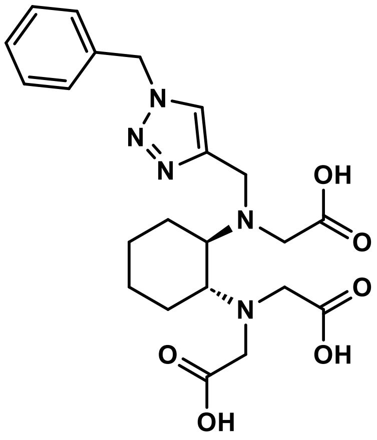

The preparation of **CHDT-Bn** was performed as described for **CHDT-Pe**. Yield*:* 316 mg (0.46 mmol; 82%). **^{1}H NMR** (CD_3CN): δ=8.06 (s, 1H, H-5 triazole), 7.43–7.26 (m, 5H, 5×CH phenyl), 5.64–5.54 (m, 2H, CH_2-phenyl), 4.59–4.38 (m, 2H), 4.15–3.96 (m, 1H), 3.96–3.80 (m, 1H), 3.71–3.01 (m, 5H), 3.00–2.75 (m, 1H), 2.19–1.98 (m, 2H), 1.88–1.67 (m, 2H), 1.52–1.14 (m, 4H); **^{13}C NMR** (CD_3CN): δ=172.63 (CO), 168.19 (CO), 159.67 (q, ^{2}J=38.7 Hz, CO TFA), 136.24 (C-1 phenyl), 129.92 (2×CH phenyl), 129.46 (C-4 phenyl), 128.95 (2×CH phenyl), 128.21 (C-5 triazole), 66.21, 61.11, 55.27, 54.77, 50.31, 49.32, 25.69, 25.07, 24.75, 24.68, signals for 1×CH/CH_2, C-4 triazole and 1×CO are not visible; m/z calculated for $C_{22}H_{30}N_5O_6$: 460.22 $[M+H]^+$, found: 461.0.

CHDT-PSMA-1 (×2TFA)

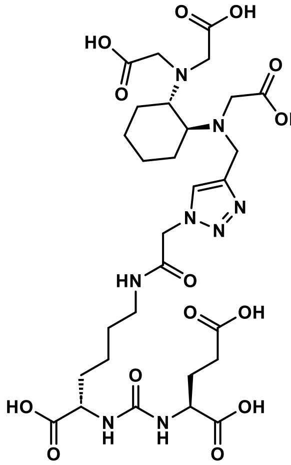

A mixture of TFA/CH_2Cl_2 (9:1, v/v, 500 µL) was slowly added to compound **9** (12.5 mg, 0.009 mmol, 1 eq.) and the resulting mixture was stirred for 18 h. UPLC-DAD-MS analysis confirmed the removal of all *tert*-butyl groups. Ice-cold diethyl ether (15 mL) was added to the reaction mixture and a white precipitate was formed. After 8 h at -20°C in a freezer, the solid was filtered, washed with ice-cold diethyl ether and dried in a desiccator, which afforded **CHDT-PSMA-1** as a white solid (11.6 mg, 60%, purity ≥93% at 214 nm). **^{1}H-NMR** (D_2O): δ=8.24 (s, 1H, triazol), 5.28 (s, 2H, triazol-CH_2-CO), 4.49 (d, ^3J = 13.8 Hz, 1H, triazol-C*H*H-N), 4.30 (dd, *J* =9.1, 5.1 Hz, 1H, C_αH), 4.22 (dd, *J* = 8.8, 4.9 Hz, 1H, C_αH), 3.72 (d, ^3J = 16.9 Hz, 1H, triazol-CH*H*-N), 3.29 (t, J = 6.7 Hz, 4H, 2×CH cyclohexane, CH_2), 2.55 (t, ^3J = 7.3 Hz, 2H, $C_\varepsilon H_2$ lysine), 2.32 – 1.25 (m, 18H, $C_\beta/C_\gamma H_2$ glutamate, $C_\beta/C_\gamma/C_\delta H_2$ lysine, 4×CH_2 cyclohexane), signals for 3×NH and 2×CH_2 are not visible; **^{13}C-NMR** (D_2O): δ=177.23 (2×CO), 177.12 (2×CO), 176.27 (2×CO), 167.32 (CO amide), 159.30 (CO urea), 62.55 (2×CH cyclohexane), 53.10 (C_α), 52.56 (C_α), 52.27 (triazol-*C*H_2-CO), 39.24 (CH_2), 30.44, 30.03 (C_ε lysine), 27.51, 26.16, 24.18, 23.73, 23.51, 22.12, signals for 2×C of triazole and 3×CH_2 are not visible, below 2.4 ppm ^{13}C signals are partially overlapped; m/z calculated for $C_{29}H_{45}N_8O_{14}$: 729.30 $[M+H]^+$, found: 729.2.

CHDT-PSMA-2 (×2TFA)

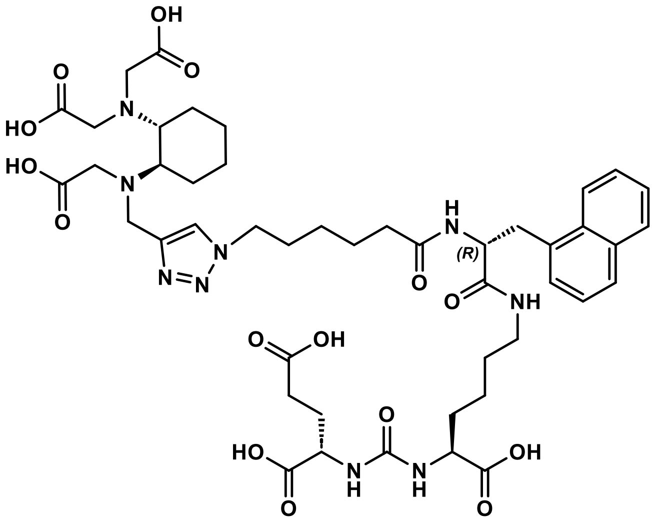

Compound **10** (190 mg, 0.14 mmol) was dissolved CH_2Cl_2/TFA (10 mL, 1:9, v/v) and stirred overnight. The deprotected product was precipitated by the addition of ice-cold diethyl ether and the precipitate then subsequent washed with 50 mL of ice-cold chloroform to yield **CHDT-PSMA-2** (180 mg, 97%, purity ≥94% at 254 nm). m/z calculated for $C_{46}H_{64}N_9O_{15}$: 982.45 $[M+H]^+$, found: 983.0.

CHDT-PSMA-3 (×2TFA)

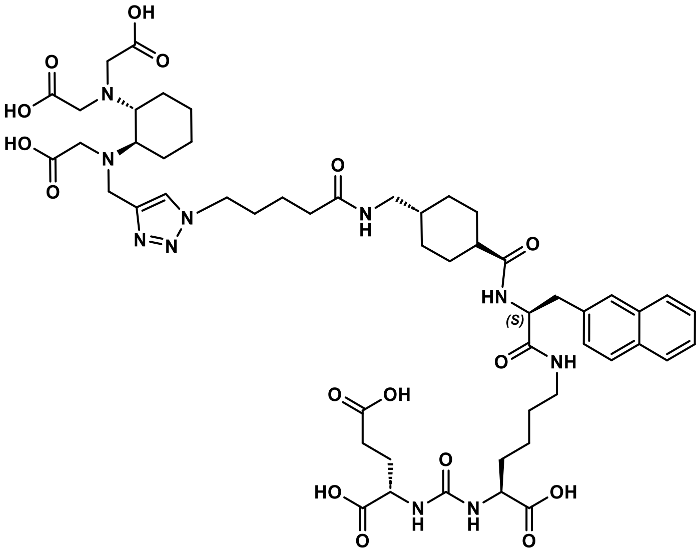

Compound **11** (10 mg, 7 µmol) was dissolved CH_2Cl_2/TFA (2 mL, 1:9, v/v) and stirred overnight. The deprotected product was precipitated by the addition of ice-cold diethyl ether and the precipitate then subsequent washed with 50 mL of ice-cold chloroform to yield **CHDT-PSMA-3** (6.5 mg, 78%, purity ≥94% at 254 nm), m/z calculated for $C_{53}H_{75}N_{10}O_{16}$: 1107.54 $[M+H]^+$, found: 1108.1.

References

1. Xiao, D.; Duan, X.; Gan, Q.; Zhang, X. and Zhang, J. Preparation and biological evaluation of [^{99m}Tc]Tc-CNGU as a PSMA-targeted radiotracer for the imaging of prostate cancer. *Molecules*, **2020**, *25*.
2. Reissig, F.; Bauer, D.; Zarschler, K.; Novy, Z.; Bendova, K.; Ludik, M. C.; Kopka, K.; Pietzsch, H. J.; Petrik, M. and Mamat, C. Towards targeted alpha therapy with Actinium-225: chelators for mild condition radiolabeling and targeting PSMA-A proof of concept study. *Cancers*, **2021**, *13*.
